# Deprotometalation-Iodolysis and Direct Iodination of 1-Arylated 7-Azaindoles: Reactivity Studies and Molecule Properties

**DOI:** 10.3390/molecules26206314

**Published:** 2021-10-19

**Authors:** Mohamed Yacine Ameur Messaoud, Ghenia Bentabed-Ababsa, Ziad Fajloun, Monzer Hamze, Yury S. Halauko, Oleg A. Ivashkevich, Vadim E. Matulis, Thierry Roisnel, Vincent Dorcet, Florence Mongin

**Affiliations:** 1 Institut des Sciences Chimiques de Rennes–UMR 6226, University of Rennes, CNRS, ISCR, 35000 Rennes, France; m.yacin31@yahoo.fr (M.Y.A.M.); thierry.roisnel@univ-rennes1.fr (T.R.); vincent.dorcet@univ-rennes1.fr (V.D.); 2 Laboratoire de Synthèse Organique Appliquée, Faculté des Sciences Exactes et Appliquées, Université d’Oran 1 Ahmed Ben Bella, BP 1524 El M’Naouer, Oran 31000, Algeria; 3 Laboratory of Applied Biotechnology (LBA3B), Azm Center for Research in Biotechnology and Its Applications, EDST, Lebanese University, Tripoli 1300, Lebanon; 4 Faculty of Sciences 3, Campus Michel Slayman, Lebanese University, Tripoli 1352, Lebanon; 5 Laboratoire Microbiologie, Santé et Environnement, Doctoral School of Sciences and Technology, Faculty of Public Health, Lebanese University, Tripoli 1300, Lebanon; mhamze@monzerhamze.com; 6 UNESCO Chair of Belarusian State University, 220030 Minsk, Belarus; 7 Research Institute for Physico-Chemical Problems, Belarusian State University, 220030 Minsk, Belarus; fhp@bsu.by (O.A.I.); matulisvad@bsu.by (V.E.M.)

**Keywords:** 7-azaindole, deprotometalation, iodination, regioselectivity, *N*-arylation

## Abstract

Five protocols were first compared for the copper-catalyzed C-N bond formation between 7-azaindole and aryl/heteroaryl iodides/bromides. The 1-arylated 7-azaindoles thus obtained were subjected to deprotometalation-iodolysis sequences using lithium 2,2,6,6-tetramethylpiperidide as the base and the corresponding zinc diamide as an in situ trap. The reactivity of the substrate was discussed in light of the calculated atomic charges and the p*K*_a_ values. The behavior of the 1-arylated 7-azaindoles in direct iodination was then studied, and the results explained by considering the HOMO orbital coefficients and the atomic charges. Finally, some of the iodides generated, generally original, were involved in the *N*-arylation of indole. While crystallographic data were collected for fifteen of the synthesized compounds, biological properties (antimicrobial, antifungal and antioxidant activity) were evaluated for others.

## 1. Introduction

Due to its similarity with indole and purine, 1*H*-pyrrolo[2,3-*b*] pyridine (7-azaindole) has aroused the interest of the chemical community [1,2,3,4,5,6,7,8], for example, for medicinal applications, as this motif can be found in molecules with of a broad spectrum of bioactivities. Mention may be made, for example, of compounds used to treat diseases involving the abnormal regulation of enzymes.

**Figure 1:**
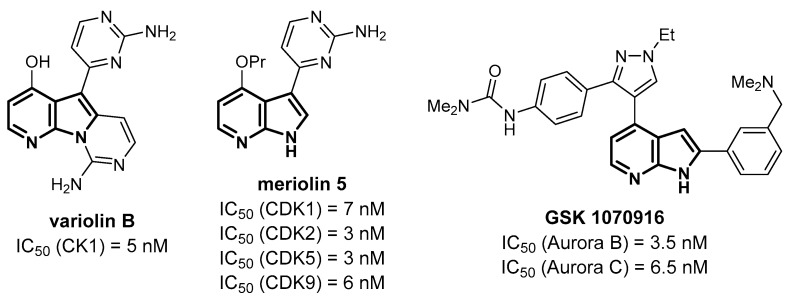
Compounds of biological interest containing a 7-azaindole core and their bioactivities.

Among them, variolin B and certain meriolins are, respectively, good inhibitors of casein and cyclin-dependent kinases and therefore promising for the treatment of brain cancers (Figure 1, left) [9,10]. Also based on a 7-azaindole, GSK 1070916 is a potent inhibitor of Aurora kinases, which are involved in the regulation of mitosis and frequently overexpressed in cancer tumors (Figure 1, right) [11].

7-Azaindole can also be present in the backbone of organic materials, for example exhibiting luminescence properties. Furthermore, due to the nucleophilicity of pyridine nitrogen, 7-azaindole derivatives can also act as ligands for catalysis [12].

While much work has been devoted to the functionalization of 7-azaindole, relatively little of it deals with the functionalization of the azaindole ring of 1-arylated derivatives. Dong and coworkers published studies in 2015–2016 in which tetracycles were formed from 1-aryl-7-azaindoles and diphenylacetylene by a rhodium(III)-catalyzed annulation reactions [13,14]. In 2016, tetracyclic heterocycles were also built by Ge, Li and coworkers from 1-arylated 7-azaindoles by using as a key step the rhodium(III)-catalyzed C-H oxidative olefination of the aryl group [15]. A year later, Mishra, Kim and coworkers converted a 1-arylated 7-azaindole to azaindoloquinoline by rhodium(III)-catalyzed C-H amination followed by intramolecular cyclization [16]. Metal-free functionalization of 1-arylated 7-azaindoles is also possible, as evidenced by the work of Xu, Dong and coworkers; in this case, another type of tetracyclic heterocycle was synthesized by TsOH-induced tandem [3 + 2] cyclization between 7-azaindoles and pyridotriazoles [17].

Our objective in the present study was to develop methods to introduce an iodine atom either at the 2- or at the 3-position of 1-aryl-7-azaindoles and to predict the outcome of these reactions [18,19] using p*K*_a_, atomic charges and HOMO orbital coefficients.

## 2. Results and Discussion

### 2.1. 1-Arylation of 7-Azaindole

Due to our interest in the copper-catalyzed *N*-arylation of azoles with aryl or heteroaryl iodides [20,21,22,23,24,25], access to the required 1-arylated 7-azaindoles was considered in this way.

Various protocols using copper-based catalysts have already been reported to 1-arylate 7-azaindole with aryl or heteroaryl halides [3,5,26]. As part of this study, we decided to compare five sets of conditions already used to *N*-arylate azoles with aryl/heteroaryl iodides or bromides. We chose (i) the simple ‘ligand-free’ copper-catalyzed *N*-arylation of azoles documented by Hu and coworkers (*Method A*: Cu (0.2 equiv), Cs_2_CO_3_ (2 equiv), acetonitrile at reflux) [27]; (ii) the lithium chloride-mediated copper(I) iodide-catalyzed 1-arylation of azaindoles reported by Yum and coworkers (*Method B*: CuI (0.1 equiv), K_2_CO_3_ (3 equiv), LiCl (1 equiv), dimethylformamide (DMF) at 120 °C) [28]; (iii) the copper-diamine method developed by Buchwald and coworkers to *N*-arylate azoles (*Method C*: CuI (5 mol%), K_3_PO_4_ (2 equiv), *N*,*N’*-dimethylethylenediamine (DMEDA; 0.1 equiv), DMF at reflux) [29]; (iv) the protocol Teo and coworkers used to *N*-arylate azoles, including 7-azaindole, with iodopyridines (*Method D*: Cu_2_O (0.1 equiv), Cs_2_CO_3_ (2 equiv), dimethylsulfoxide (DMSO) at 110 °C) [30]; and (v) the ‘ligand-free’ procedure Yum and coworkers employed to *N*-arylate carbazole under microwave irradiation (*Method E*: CuI (0.1 equiv), Cs_2_CO_3_ (1 equiv), DMF, MWI at 350 W) [31].

The results are shown in Table 1. The reactions performed from iodobenzene (entries 1–5) and 4-iodoanisole (entries 6–10) were more efficiently carried out with methods using a ligand (DMEDA for *Method C*, or DMSO for *Method D*). Therefore, we applied them to 1-arylate 7-azaindole with 1-chloro-4-iodobenzene (entry 11), 1-fluoro-4-iodobenzene (entry 12), 1-iodo-4-(trifluoromethyl)benzene (entries 13 and 14), 1-iodo-3,5-dimethylbenzene (entry 15), 2-iodothiophene (entries 16 and 17), 3-iodopyridine (entries 18 and 19), 2-bromopyridine (entries 20 and 21) and 4-bromopyridine (entry 22). *Method D* worked well with all tested iodides but was less effective with bromides. *Method C* appeared to be a higher-yielding protocol, whether for the iodides tested or for 2-bromopyridine; this is not surprising since this method has been shown to be effective for substrates known to be reluctant to *N*-arylation such as iodoferrocenes [32,33].

**Scheme 1:**
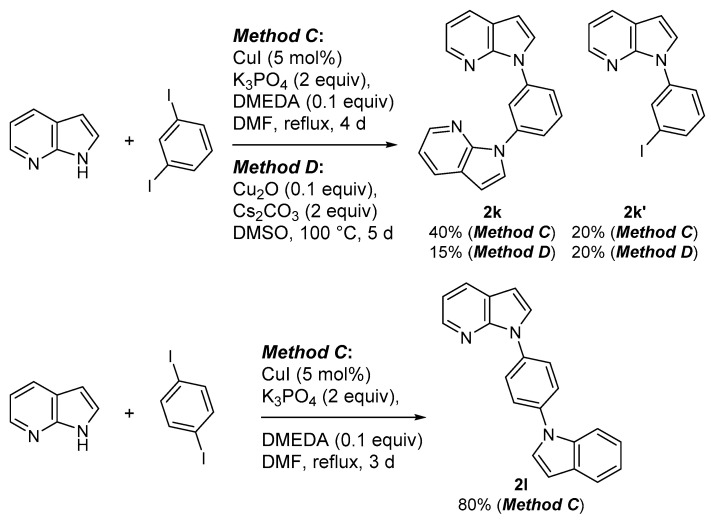
1-Arylation reactions between 7-azaindole and 1,3- or 1,4-diodobenzene.

Concerning the double *N*-arylation reaction between 7-azaindole and 1,3-diiodobenzene, *Method C* also provided the expected product **2k** with a higher yield (40%) than *Method D* (15%). However, despite long reaction times of 4–5 days, the product **2k′** resulting from a single *N*-arylation is still present (isolated in 20% yield in both cases) (Scheme 1, top). By contrast, when *Method C* was applied to the reaction between 7-azaindole and 1,4-diiodobenzene, the expected product **2l** was this time obtained with a high 80% yield (Scheme 1, bottom).

**Figure 2:**
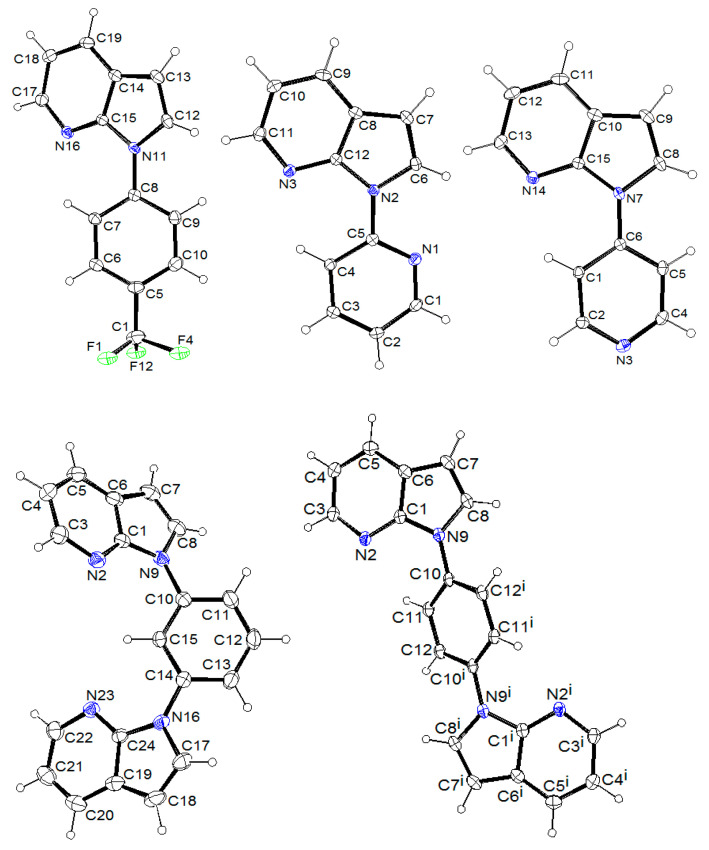
ORTEP diagrams (30% probability) of **1e**, **1i**, **1j**, **2k** and **2l**.

The compounds **1e**, **1i**, **1j**, **2k** and **2l** were unambiguously identified by X-ray diffraction (Figure 2). Due to steric hindrance, their two or three rings have not been shown to be coplanar. While the torsion angles were at most 25° in the case of **1e** (25.1°), **1i** (2.3/10.9°), **1j** (14.2°) and **2l** (25.0° and 25.0°), a higher value was observed for **2k** (22.7° and 35.8°). This could explain why lower yields were noticed for **2k** compared to **2l**.

**Figure 3:**
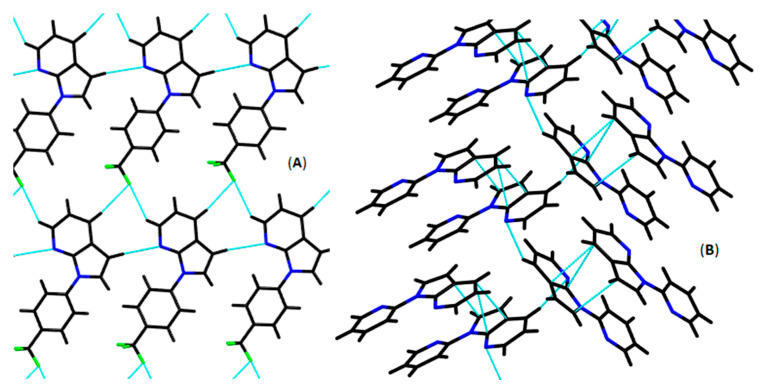
Short-contact networks observed for **1e** (**A**) and **1i** (**B**).

The X-ray diffraction data recorded for the compounds **1e** and **1i** deserve some additional comments. In the case of **1e**, short intermolecular contacts were observed at the solid state between the pyridine nitrogen of azaindole and the hydrogen at C3 (2.644 Å), causing a linear chain, while a fluorine of the trifluoromethyl group is close to the hydrogens of two different azaindoles of another chain, one at C4 (2.651 Å) and the other at C6 (2.625 Å) (Figure 3A). In the case of **1i**, the molecules are arranged in pairs of two parallel azaindoles (separated by about 3.3 Å); these pairs are connected by short contacts between the pyridine nitrogen of the azaindole of one pair and the hydrogen at C3 of the azaindole of another pair (2.609 Å) (Figure 3B). Overall, the XRD geometries are very close to those predicted by DFT calculations (see Supplementary Materials), including the most stable rotamer form.

### 2.2. Deprotometalation-Iodolysis of 1-Arylated 7-Azaindoles

Deprotolithiation-trapping sequences at the 2-position of 7-azaindoles have largely been developed after protection of the NH [3]. In 1997, Mérour and coworkers reported the first studies on the topic from the 1-phenylsulfonyl derivative; the key deprotolithiation step was carried out by using lithium diisopropylamide (2 equiv) in tetrahydrofuran (THF) at −25 °C for 0.5 h and was evidenced by subsequent trapping with various electrophiles [34]. However, probably because the sulfonamide function also activates the phenyl group [35], a second deprotonation at the phenyl ring was noticed with electrophiles such as chlorotrimethylsilane and chlorotrimethylstannane which are known for their greater compatibility with hindered lithium amides. In 2007, Kondo and coworkers identified mesityllithium as an alternative base to the lithium amide for similar substrates [36].

One way to deprotometalate the 2-position without protection/deprotection steps is to form a carbamate in situ. In 1999, Curtis and coworkers extended this approach, first developed in the indole series by Katritzky and coworkers [37], to 7-azaindole. They successively treated 7-azaindole with *n*-butyllithium and carbon dioxide before performing C2-deprotolithiation with *tert*-butyllithium in THF at −78 °C, subsequent electrophilic trapping (CO_2_) and acidic treatment; under these conditions, the carboxylic acid was obtained with a correct yield [38].

The 7-azaindoles 1-substituted by methyl, diethylaminomethyl and methoxymethyl groups can also be functionalized at their 2-position after deprotolithiation using *tert*-butyllithium in THF (addition of the base at −78 °C before warming to 0 °C). This was evidenced in 2008 by O’Shea, Tacke and coworkers who successfully employed 6-(dimethylamino)fulvene as an electrophile [39]. It is worth noting that 2-(trimethylsilyl)ethoxymethyl can also be employed as a protecting group to easily deprotonate the adjacent site [40,41]. As might be expected, when tri(isopropyl)silyl is present on 7-azaindole N1, the 5-membered ring is protected from deprotometalation [42].

In the absence of a substituent at N1, it is possible to reroute the reaction on the pyridine ring by benefiting from an anionic shielding in situ, thanks to an efficient directing group (such as *N*,*N*-diethylcarboxamide or *N*,*N*-diethylsulfonamide) at the 3- and possibly 4-position. This was demonstrated by Snieckus and coworkers in 2012 [43]. The same group more recently identified *N*,*N*-diisopropylcarboxamide as a directing group for the introduction of different substituents at C2 after deprotolithiation using lithium diisopropylamide (2 equiv) in THF at −78 °C. An elegant dance of directed metalation-groups was also developed by the same authors to successively functionalize the 6 (directing group onto the N7 nitrogen) and 2 (directing group onto the N1 nitrogen) positions [44].

To our knowledge, the only 1-aryl-7-azaindole already involved in deprotolithiation-trapping is 1-(2-pyridyl)-7-azaindole. Wang and coworkers introduced a deuterium or a methyl group at its 2-position by reaction with lithium diisopropylamide (2.5 equiv) in diethyl ether at −78 °C for 40 min and subsequent trapping with heavy water or iodomethane, respectively [45]. Our goal in this part is to evaluate the scope of the deprotometalation of 1-aryl-7-azaindoles by using as substrates the compounds **1** described above. We used a common electrophile in order to easily compare the reactivities of our substrates, and we chose iodine since it is possible to take advantage of this heavy halogen to carry out postfunctionalizations such as Suzuki-Miyaura [3,46], Sonogashira [3] or even Heck [3] couplings.

**Figure 4:**
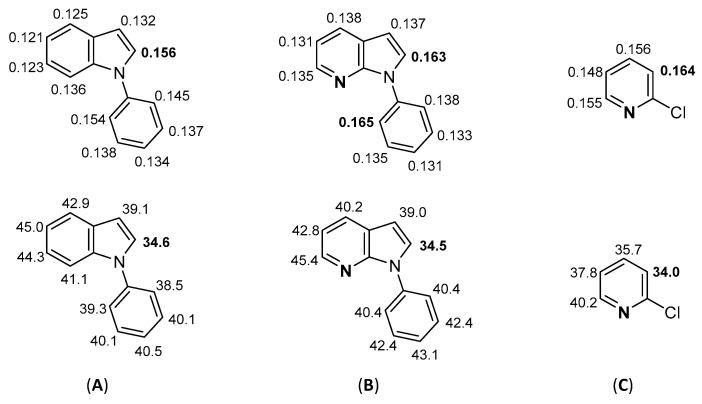
Calculated H charges (top) and p*K*_a_ values (bottom) of 1-phenylindole [52] (**A**), 1-phenyl-7-azaindole (**1a**; **B**) and 2-chloropyridine [53] (**C**).

Previous results from the group showed that 1-phenylindole could be efficiently deprotonated at C2 by using a base prepared from ZnCl_2_∙TMEDA (TMEDA = *N*,*N*,*N*′,*N*′-tetramethylethylenediamine) and LiTMP (TMP = 2,2,6,6-tetramethylpiperidino) in a 1:3 ratio [47,48,49]. Indeed, after treatment of the substrate by this formed 1:1 LiTMP-Zn(TMP)_2_ mixture of amides [47,50,51] in THF at room temperature (rt) and addition of iodine to intercept the heteroarylmetal obtained, 2-iodo-1-phenylindole was isolated with a yield of 92% [52]. Under these conditions, it is accepted that LiTMP deprotolithiates the heterocycle and that the heteroaryllithium is then trapped by a zinc species through transmetalation [47,50,51]. The calculated hydrogen atomic charges (H charges) and C-H p*K*_a_ values [52] of 1-phenylindole (Figure 4A) are in good agreement with the observed results, with both the most positive hydrogen and the most stabilized lithiated compound at C2.

To our knowledge, the acidic properties of 7-azaindoles have not yet been investigated. The most related experimental study is that of Fraser and coworkers [54], who determined the p*K*_a_ values in THF of five-membered heteroaromatic C-H acids, including *N*-methylindole. In 2014–2015, we also computed the p*K*_a_ values of *N*-arylated pyrroles and indoles [52] as well as 1-arylated benzotriazoles and indazoles [55] by using DFT.

In general (except for C5 and C6 which are reversed), the p*K*_a_ distribution pattern of 1-aryl-7-azaindoles resembles that of 1-arylindoles [52]. In the case of 1-phenyl-7-azaindole (**1a**), the most positively charged hydrogens are located at phenyl C2′ (opposite to the pyridine nitrogen in bold) and azaindole C2 (next to the pyrrole nitrogen) while the most stabilized lithiated derivative is also at C2 (Figure 4B). It is interesting to know which of the lithiated intermediates is intercepted by Zn(TMP)_2_ during such a LiTMP-mediated deprotonation.

For this purpose, **1a** was treated at rt for 2 h by the basic combination prepared in situ in THF from ZnCl_2_∙TMEDA (1 equiv) and LiTMP (3 equiv), and iodine was added to quench the organometallic intermediate. Under these conditions, the 2-iodinated derivative **3a** was isolated with a yield of 75% (Table 2, entry 1). This can result either from direct deprotolithiation at C2 or from deprotonation at another position (directed by the pyridine nitrogen) followed by isomerization, before transmetalation to a zinc species. This situation could parallel that of 2-chloropyridine for which the kinetic lithiated product is at C6 (due to the presence of a neighboring coordinating pyridine nitrogen) and the thermodynamic product at C3 (both highest H charge and lower p*K*_a_ value [53]) [56] (Figure 4C).

**Figure 5:**
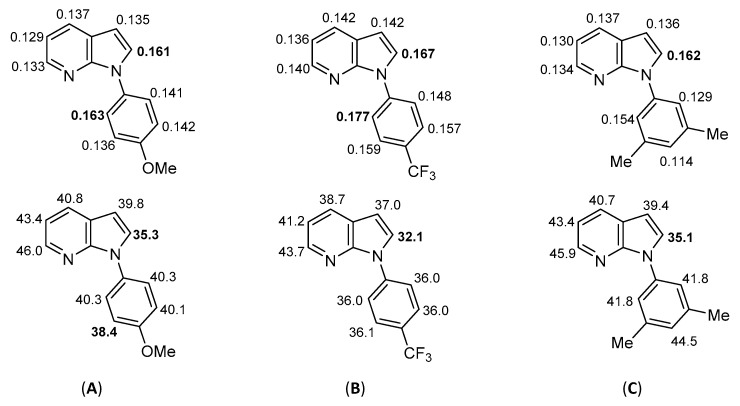
Calculated H charges (top) and p*K*_a_ values (bottom) of 1-(4-methoxyphenyl)-7-azaindole (**1b**; **A**), 1-(4-(trifluoromethyl)phenyl)-7-azaindole (**1e**; **B**) and 1-(3,5-dimethylphenyl)-7-azaindole (**1f**; **C**).

When a substituent such as OMe and especially CF_3_ is present at the 4-position of the phenyl, the hydrogens at C2′ still compete with those at C2 to be the most positively charged, but this time the p*K*_a_ values are lowered at the remaining free sites of the phenyl (Figure 5A,B). Consequently, other aryllithiums could be formed and intercepted by Zn(TMP)_2_ before isomerization to 2-(7-azaindolyl)lithium. It is therefore no surprise that inseparable mixtures coming from deprotonations at C2 and at the 4-methoxyphenyl were obtained from **1b** under these conditions (not shown). In the case of **1e**, both the 2-iodinated derivative **3e** and the 2,3′-diiodinated derivative **3e′** were obtained (in yields of 10 and 20%, respectively) from such a complex mixture (Table 2, entry 2); this could result from a second deprotonation at the phenyl of the 2-(7-azaindolyl)zinc species [57]. By contrast, from 1-(3,5-dimethylphenyl)-7-azaindole (**1f**), no competitive reaction on the phenyl ring took place, as expected from the calculated p*K*_a_ values (Figure 5C); the only 2-iodinated derivative **3f** was isolated with a yield of 40% (Table 2, entry 3).

**Figure 6:**
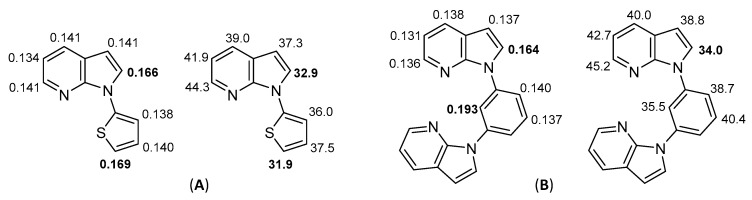
Calculated H charges and p*K*_a_ values of 1-(2-thienyl)-7-azaindole (**1g**; **A**) and 1,1′-(1,3-phenylene)bis(7-azaindole) (**2k**; **B**).

As indicated by the corresponding p*K*_a_ values, 2-thienylmetals are easier to obtain due to their greater stability than, for example, phenylmetals (Figure 6A). Therefore, when subjected to our lithium–zinc basic combination prior to iodolysis, **1g** did not lead to the 2-iodinated product. Instead, 1-(5-iodo-2-thienyl)-7-azaindole (**3g**; isolated in 30% yield) and the diiodinated derivative **3g′** (40% yield) were formed (Table 2, entry 4). Again, due to similar p*K*_a_ values (Figure 6B) or a proximity effect [57], a mixture of the three products **3k**, **3k′** and **3k″** was obtained when **2k** was processed in the same reaction conditions (Table 2, entry 5).

Thus, by calculating the p*K*_a_ values of these 1-aryl-7-azaindoles, it is quite easy to predict whether the reaction leads to a main iodinated derivative or whether mixtures are expected.

**Figure 7:**
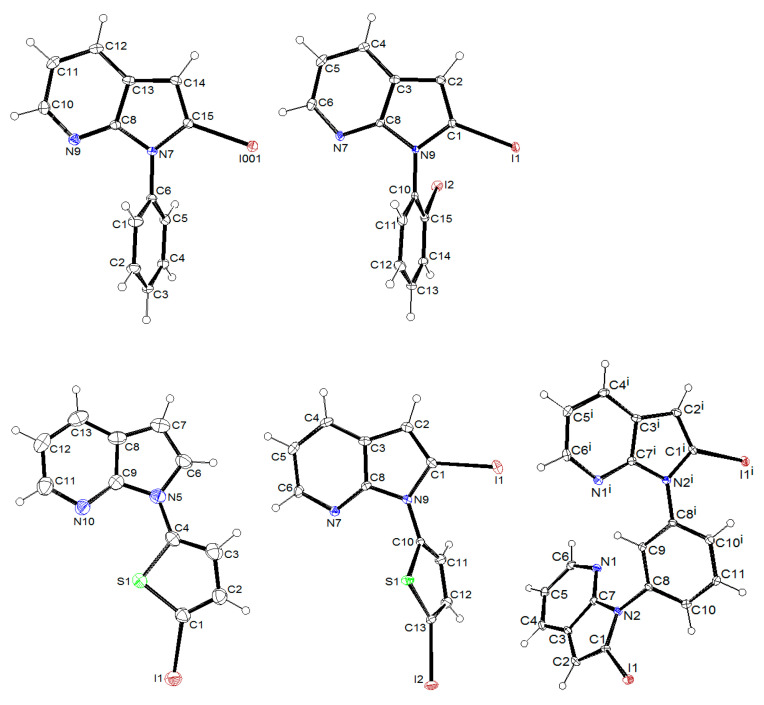
ORTEP diagrams (30% probability) of **3a**, **3a′**, **3g**, **3g′** and **3k′**.

**Figure 8:**
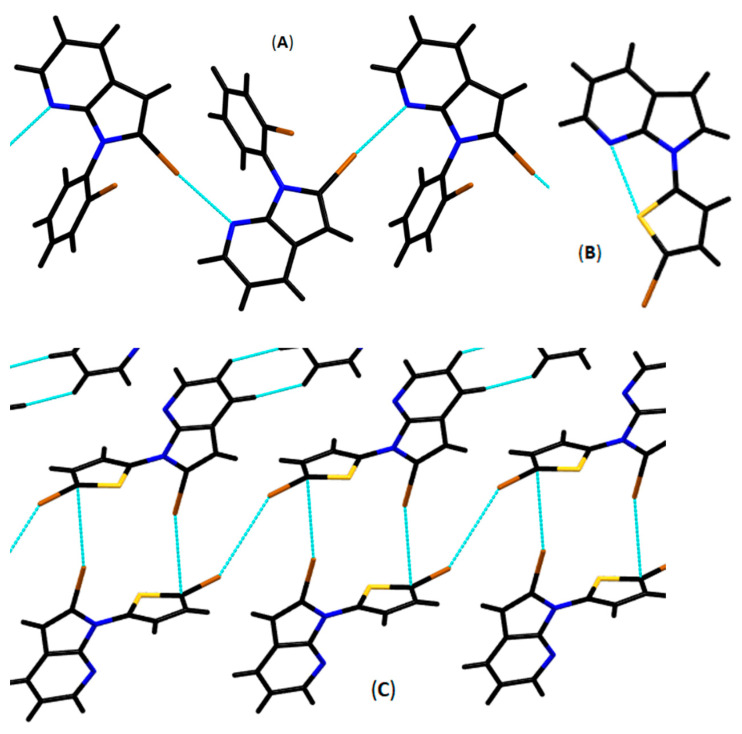
Halogen and chalcogen bond networks observed for **3a′** (**A**) and **3g** (**B**), respectively; short contact involving iodine atoms for **3g′** (**C**).

Among the X-ray diffraction data collected to unambiguously assign a structure to the isolated products (Figure 7), a few elements are noticeable. Halogen bonds [58] between pyridine nitrogen and iodine at C2 (2.932 Å) have been identified at the solid state in the case of **3a′** whereas they do not exist for the monoiodide **3a** (Figure 8A). Moreover, an intramolecular chalcogen bond [59] between pyridine nitrogen and thiophene sulfur (2.962 Å) exists for **3g** (Figure 8B). Finally, short contacts were observed in the case of **3g′**, between thiophene iodines (3.948 Å), as well as between iodine at C2 and thiophene iodine-bearing carbon (3.633 Å) (Figure 8C).

### 2.3. Direct Iodination of 1-Arylated 7-Azaindoles

The incorporation of iodine atoms at the 3-position of 7-azaindole can be used to introduce different aryl or heteroaryl groups [60,61,62,63,64], as well as other functions [8,65]. Our objective in this part is to predict the outcome of this aromatic electrophilic substitution (S_E_Ar) in the case of 1-arylated 7-azaindoles. Indeed, if 7-azaindole can be easily iodinated at its 3-position in DMF containing iodine after treatment by potassium hydroxide [43], the behavior of 1-aryl-7-azaindoles has only been the subject of very few studies.

**Figure 9:**
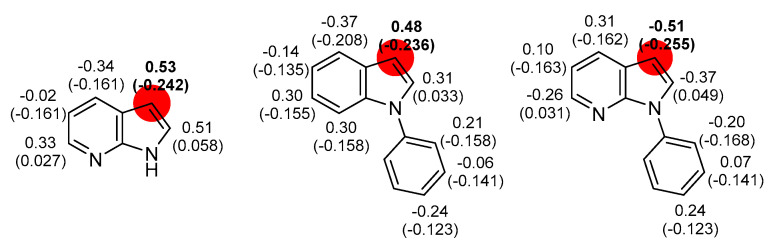
Calculated C charges (in brackets) and HOMO coefficients obtained by using the HuLiS calculator [73] for 7-azaindole, 1-phenylindole and 1-phenyl-7-azaindole (**1a**).

Liu, Xu and coworkers showed in 2014, during the rhodium-catalyzed chlorination of 7-azaindoles, that 3-iodo-1-phenyl-7-azaindole could be obtained by reacting **1a** with *N*-iodosuccinimide (NIS; 1 equiv) and potassium hydroxide (3 equiv) in acetonitrile at rt for 11 h [66]. Inspired by their protocol, we replaced NIS with iodine and isolated **1a** with a yield of 65% (Table 3, entry 1). The present reaction takes place at the carbon site possessing the most negative atomic charge (C charge; Figure 9). Another way to rationalize the regioselectivity of S_E_Ar reactions [67,68] is to use Fukui’s concept (an aromatic compound reacts with an electrophile at its carbon having the highest orbital coefficient of HOMO in absolute value) [69] and thus to calculate the HOMO coefficients by applying Hückel’s theory [70,71,72] (Figure 9). In the present case, both approaches converge toward a S_E_Ar at C3.

**Figure 10:**
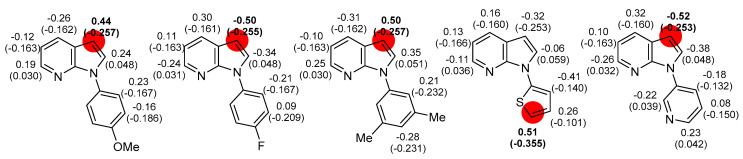
Calculated C charges (in brackets) and HOMO coefficients (obtained by using the HuLiS calculator [73]) for 1-(4-methoxyphenyl)-7-azaindole (**1b**), 1-(4-fluorophenyl)-7-azaindole (**1d**), 1-(3,5-dimethylphenyl)-7-azaindole (**1f**), 1-(2-thienyl)-7-azaindole (**1g**) and 1-(3-pyridyl)-7-azaindole (**1h**).

Next, we determined the charges and HOMO coefficients for the 1-arylated 7-azaindoles to attempt a prediction of the experimental results. For the 7-azaindoles bearing a substituted phenyl group **1b**–**1f**, all the maximum HOMO coefficients and the most negative C charges were found at C3 (Figure 10). Experimentally, the 3-iodinated derivatives **4b**, **4c**, **4d** and **4f** were indeed the only products formed, as expected; they were isolated in yields ranging from 40% to 62% (Table 3, entries 2, 3, 4 and 7). It is interesting to note that the yield of the 3-iodinated product can be slightly improved by carrying out the reaction at 40 °C with an excess of iodine, as for example noticed in **1e** (Table 3, entries 5 and 6).

Since thiophene is also a five-membered heteroaromatic prone to S_E_Ar, it was interesting to consider the iodination of 1-(2-thienyl)-7-azaindole (**1g**). Indeed, the calculations carried out as before showed a maximum HOMO coefficient and a most negative C charge at the 5-position of the thienyl ring (Figure 10). Experimentally, we obtained the 5-iodinated derivative **3g** (Table 3, entry 8), which is the compound already formed by deprotometalation-iodolysis (Table 2, entry 4). With regard to 1-pyridyl-7-azaindoles **1h**-**1j**, direct iodination at C3 is expected (Figure 10). As assumed, by carrying out the reaction from the 3- and 2-pyridyl substrates, we observed the formation of the 3-iodinated derivatives **4h** and **4i** as the only reaction products (Table 3, entries 9–12). Therefore, it appears that HOMO coefficients and carbon atomic charges can be used to predict the regioselectivity of S_E_Ar iodination reactions.

**Figure 11:**
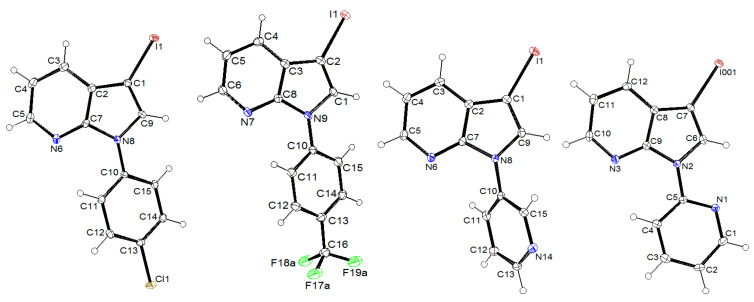
ORTEP diagrams (30% probability) of **4c**, **4e**, **4h** and **4i**.

**Figure 12:**
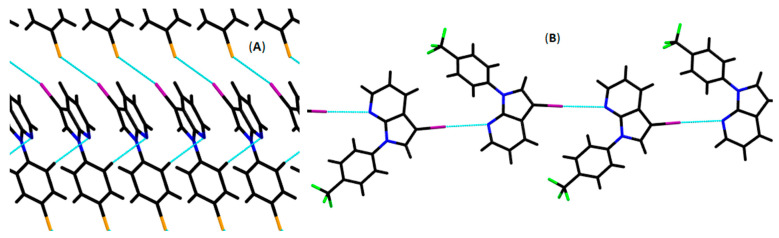

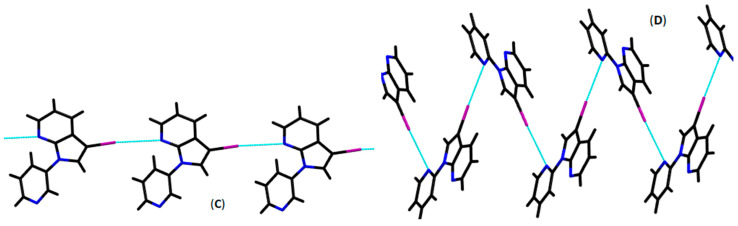
Short-contact network observed for **4c** (**A**); halogen bond networks observed for **4e** (**B**), **4h** (**C**) and **4i** (**D**).

Throughout this study, the regioselectivity was established by NMR and confirmed for the products **4c**, **4e**, **4h** and **4i** by X-ray diffraction (Figure 11). For **4c**, short intermolecular contacts were observed at the solid state between the nitrogen of the azaindole pyridine and the hydrogen at C2′ (2.734 Å), at the origin of a linear chain, while these chains are linked by short chlorine-iodine contacts (3.569 Å) (Figure 12A). The molecular networks of **4e**, **4h** and **4i** all exhibit intermolecular halogen bonds [58] in which the iodine atoms are connected to the pyridine nitrogens. For **4e** (Figure 12B) and **4h** (Figure 12C), these weak interactions bind the heavy halogen and the nitrogen of the azaindole pyridine and thus create linear chains (with iodine-nitrogen distances alternating between 3.137 and 3.158 Å for the first and 3.497 Å for the second). In the case of **4i**, the iodine is instead linked to the 2-pyridyl attached to the azaindole core, this time establishing a zig-zag chain (iodine-nitrogen distance of 3.283 Å) (Figure 12D).

### 2.4. N-Arylation of Indole by a Few of the Prepared Iodides

Because the *N*-arylation of indole with such iodinated 7-azaindoles can lead to original molecules with properties for potential applications [74,75,76], we selected the iodides **4e** and **4i** as well as the diiodide **3g′** for this purpose (Table 4). As already generally observed in Table 1, Method C has proved to be superior to Method D; in fact, while the trifluoromethylated product **5e** was only isolated with a yield of 30% (entry 1), that containing pyridine **5i** was obtained with a yield greater than 50% (entry 2).

Copper-catalyzed double *N*-arylation reactions are often difficult to perform due to competitive deiodination under the conditions required for second coupling [77]. However, despite a complex reaction mixture, the expected biscoupled product **6g′** was here isolated with a moderate yield of 20% (entry 3).

**Figure 13:**
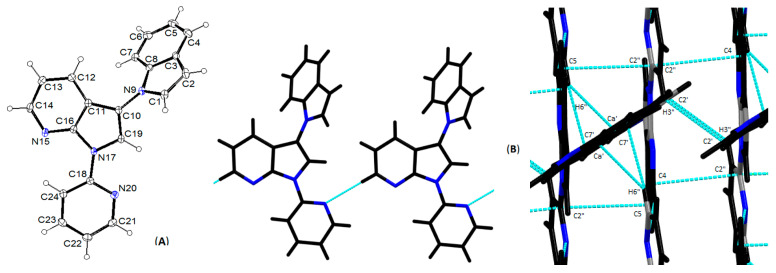
ORTEP diagrams (30% probability) of **5i** (**A**); short-contact networks observed for **5i** (**B**).

In the case of **5i**, crystals suitable for X-ray diffraction (Figure 13A) made it possible to detect several short intermolecular contacts at the solid state. First, 2-pyridyl nitrogens are close to azaindole H6 (2.638 Å), the origin of linear chains. The 2-pyridyl ring is almost coplanar with the azaindole backbone (6.37°), with its nitrogen positioned outward, while the indole ring makes a 50.6° twist angle, presumably to reduce the steric hindrance between the two bicyclic cores. These linear chains are in parallel plans successively separated by 3.368 Å (azaindole C4-pyridine C2″; see Table 4, entry 2 for numbering) and 3.399 Å (azaindole C5-pyridine C2″). Finally, short distances can be found between pyridine H6″ and both indole C7′ (2.795 Å) and Ca′ (2.878 Å), and between pyridine H3″ and indole C2′ (2.866 Å) (Figure 13B).

### 2.5. Biological Evaluation

As 7-azaindole is present in bioactive molecules, some of the compounds synthesized have been screened for their biological properties [77,78,79]. The compounds **1a**–**c**, **1e**, **1f**, **1h**-**j**, **2k**, **2l**, **3a**, **3g**, **3g′** and **5i** were evaluated for their antimicrobial activity against bacteria and for their antifungal activity (Table 5). No clear effect on microbial growth of strains of *E. coli*, *P. aeruginosa* and *S. aureus* was detected. For **1i** and **1j**, an effect on the growth of *E. faecium* was noticed. However, the most significant growth inhibitions were found for *L. monocytogenes* (compound **1j**) and *C. dubliniensis* (compounds **1a**, **1b**, **1h** and **1i**).

The antioxidant properties of a few selected compounds were finally evaluated. As shown in Table 6, the compounds **1b**, **1c**, **1f**, **1i**, **1j** and **5i** have about 50% activity. The hemolytic activity of **1b**, **1f**, **1i** and **1j**, which were found active against *Candida dubliniensis* or *Listeria monocytogenes*, was also evaluated and found to be less than 15% at a concentration of 5 μg/μL. This low toxicity on human red blood cells, for compounds exhibiting an antimicrobial effect, demonstrates a specific antibacterial or antifungal effect and thus highlights a possible therapeutic interest. Concerning **1a**, **1h** and **3a**, all active against *Candida dubliniensis*, the hemolytic activity tested at a concentration of 10 μg/μL was found to be approximately 22%, 65% and 32%, respectively (data not shown).

## 3. Materials and Methods

### 3.1. General Information

Column chromatography separations were achieved on silica gel (40–63 μm). Melting points were measured on a Kofler apparatus. InfraRed (IR) spectra were taken on an ATR Spectrum 100 spectrometer (Perkin-Elmer, Waltham, MA, USA) and the main absorption wavenumbers are given in cm^−1^. ^1^H and ^13^C nuclear magnetic resonance (NMR) spectra were recorded on an Avance III spectrometer (291 K) at 300 and 75 MHz, respectively (Bruker, Billevica, MA, USA). ^1^H chemical shifts (δ) are given in ppm relative to the solvent residual peak, and ^13^C chemical shifts are relative to the central peak of the solvent signal [80]. ZnCl_2_∙TMEDA was prepared as reported previously [81].

### 3.2. Crystallography

The samples were studied with monochromatized Mo-Kα radiation (λ = 0.71073 Å). The X-ray diffraction data were collected at the temperature given in the product description by using either APEXII Bruker AXS diffractometer (graphite monochromator; compounds **1e**, **2l**, **3g**, **3g′**, **3k′**, **4e**, **4h**, **4i** and **5i**), or D8 VENTURE Bruker AXS diffractometer equipped with a (CMOS) PHOTON 100 detector (multilayer monochromator; compounds **1e**, **1i**, **1j**, **2k**, **3a**, **3a′** and **4c**). For the compounds **1e**, **1i**, **1j**, **2l**, **3a**, **3g′**, **1e** and **4c**, the structure was solved by direct methods using the SIR97 program [82] and then refined with full-matrix least-square methods based on F2 (SHELXL-97) [83] with the aid of the WINGX [84] program. All nonhydrogen atoms were refined with anisotropic atomic displacement parameters. H atoms were finally included in their calculated positions. For the compounds **2k**, **3a′**, **3g**, **3k′**, **4e**, **4h**, **4i** and **5i**, the structure was solved by dual-space algorithm using the SHELXT program [85] and then refined with full-matrix least-square methods based on F2 (SHELXL-2014) [86]. All nonhydrogen atoms were refined with anisotropic atomic displacement parameters. H atoms were finally included in their calculated positions. The molecular diagrams were generated by ORTEP-3 (version 2.02, University of Glasgow, Glasgow, UK) [87].

### 3.3. Computational Details

The DFT calculations were performed by using GAUSSIAN 09 package [88]. The B3LYP hybrid functional was employed. All optimized geometries were obtained by using the 6–31G(d) basis set without any symmetry constraints. The vibrational frequencies were calculated at the same level of theory in order to characterize stationary points and calculate zero-point vibrational energies (ZPVE) and thermal corrections. The total energy of species was found by using the 6–311 + G(d,p) basis set. Further, the gas-phase Gibbs energies (*G*^0^_298_) were calculated by using Equation (1), as follows:

*G*^0^_298_ = *E* + ZPVE + *H*^0^_0→298_ − *TS*^0^_298_ (1)

The gas-phase acidity Δ*G*_acid_ was defined as the Gibbs energy of deprotonation of the corresponding substrate R–H (R–H(g) → R^−^(g) + H^+^(g)):

Δ*G*_acid_ = *G*^0^_298_(R^−^) + *G*^0^_298_(H^+^) − *G*^0^_298_(R-H) (2)

The solvent effect was simulated within the polarized continuum model (PCM) with the default parameters for THF [89]. The PCM energies were calculated at the B3LYP/6–311 + G(d,p) level by using geometries optimized for isolated structures.

The following homodesmic reaction was composed for the p*K*_a_ values calculation:

R-H(s) + Het^−^(s) → R^−^(s) + Het-H(s) (3)

where Het–H is *N*-methylindole. The latter was chosen as reference compound due to its structural similarity and since its p*K*_a_(THF) = 38.1 reported by Fraser et al. [54] was expected to be close to the substrates under consideration. Consequently, the Gibbs energy of the homodesmic reaction (Δ*G*_r,s_) and the p*K*_a_ value are related by the following equation:

(4) $$pK_{a}=38.1+\frac{1}{2.303}\cdot \frac{\Delta G_{r,s}}{RT}$$

The atomic charges were calculated by using Mulliken population analysis. MO coefficients were generated by using the HuLiS calculator [73].

### 3.4. 1-Arylation of 7-Azaindole

#### 3.4.1. General Procedure 1 Using Copper

This was adapted from a reported protocol [27]. A mixture of 7-azaindole (0.18 g, 1.5 mmol), aryl iodide (1.0 mmol) or diiodide (0.50 mmol), Cu (13 mg, 0.20 mmol), Cs_2_CO_3_ (0.65 g, 2.0 mmol) in acetonitrile (1 mL) was heated at reflux under Ar (the reaction time is given in the product description). The reaction mixture was cooled to rt before addition of EtOAc (20 mL) and filtration. The solvent was removed under reduced pressure, and the crude was purified by chromatography over silica gel (the eluent is given in the product description).

#### 3.4.2. General Procedure 2 Using Copper(I) Iodide without Ligand

This was adapted from a reported protocol [28]. A mixture of 7-azaindole (0.12 g, 1.0 mmol), aryl iodide (1.1 mmol), CuI (19 mg, 0.10 mmol), K_2_CO_3_ (0.41 g, 3.0 mmol) and LiCl (42 mg, 1.0 mmol) in DMF (1 mL) was heated at 120 °C for 24 h under Ar. The reaction mixture was cooled to rt before addition of an aqueous saturated solution of NH_4_Cl (20 mL). Extraction with EtOAc (3 × 20 mL), drying over MgSO_4_, removal of the solvent under reduced pressure, and purification of the crude over silica gel (the eluent is given in the product description) gave the product.

#### 3.4.3. General Procedure 3 Using Copper(I) Iodide with Ligand

This was adapted from reported protocols [29]. A mixture of 7-azaindole (0.12 g, 1.0 mmol), aryl iodide (1.2 mmol) or diiodide (0.60 mmol), CuI (9.5 mg, 50 μmol), K_3_PO_4_ (0.42 g, 2.0 mmol) and DMEDA (11 μL, 0.10 mmol) in DMF (1 mL) was degassed and heated at reflux under Ar (the reaction time is given in the product description). The reaction mixture was cooled to rt. The residue was taken with EtOAc (20 mL) and filtrated over celite. Removal of the solvent under reduced pressure and purification of the crude over silica gel (the eluent is given in the product description) gave the product.

#### 3.4.4. General Procedure 4 Using Copper(I) Oxide

This was adapted from a reported protocol [30]. A mixture of 7-azaindole (0.24 g, 2.0 mmol), aryl iodide (1.0 mmol) or diiodide (0.50 mmol), Cu_2_O (14 mg, 0.10 mmol) and Cs_2_CO_3_ (0.65 g, 2.0 mmol) in DMSO (1 mL) was heated at 110 °C under Ar (the reaction time is given in the product description). The reaction mixture was cooled to rt. The residue was taken with EtOAc (20 mL) and filtrated over celite. Removal of the solvent under reduced pressure and purification of the crude over silica gel (the eluent is given in the product description) gave the product.

#### 3.4.5. General Procedure 5 Using Copper(I) Iodide and Microwaves

This was adapted from a reported protocol [31]. A mixture of 7-azaindole (0.12 g, 1.0 mmol), aryl iodide (1.1 mmol), CuI (19 mg, 0.10 mmol) and Cs_2_CO_3_ (0.33 g, 1.0 mmol) in DMF (1 mL) was heated in a Whirpool M571 domestic microwave oven at 350 W (the reaction time is given in the product description). The reaction mixture was cooled to rt before addition of an aqueous saturated solution of NH_4_Cl (20 mL). Extraction with EtOAc (3 × 20 mL), drying over MgSO_4_, removal of the solvent under reduced pressure, and purification of the crude over silica gel (the eluent is given in the product description) gave the product.

#### 3.4.6. 1-Phenyl-1*H*-pyrrolo[2,3-*b*]pyridine or 1-Phenyl-7-azaindole (**1a**)

The general procedure 1 (reaction time: 29 h), 2 (24 h), 3 (30 h), 4 (24 h) and 5 (15 min) using iodobenzene (0.11, 0.12, 0.13, 0.11 and 0.12 mL, respectively) afforded (eluent: hexane-EtOAc 90:10) the title product in 20%, 15%, 75%, 75% and 18% yield, respectively, as a yellow oil. IR: 689, 705, 719, 751, 769, 796, 893, 954, 1029, 1072, 1111, 1146, 1179, 1210, 1235, 1269, 1288, 1322, 1360, 1421, 1457, 1476, 1496, 1513, 1590, 1731, 1863, 2582, 3047. ^1^H-NMR (CDCl_3_): 6.63 (d, 1H, *J* = 3,6 Hz, H3), 7.15 (dd, 1H, *J* = 7.8 and 4.7 Hz, H5), 7.35 (tt, 1H, *J* = 7.4 and 1.2 Hz, H4′), 7.50–7.57 (m, 3H, H2 and Ph), 7.77–7.81 (m, 2H, Ph), 7.98 (dd, 1H, *J* = 7.8 and 1.6 Hz, H4), 8.43 (dd, 1H, *J* = 4.7 and 1.6 Hz, H6). ^13^C-NMR (CDCl_3_): 101.8 (CH, C3), 116.9 (CH, C5), 121.8 (C, Cb), 124.2 (2CH, C2′ and C6′), 126.5 (CH, C2 or C4′), 128.1 (CH, C2 or C4′), 129.3 (CH, C4), 129.6 (2CH, C3′ and C5′), 138.7 (C, C1′), 143.8 (CH, C6), 147.7 (C, Ca). These data are as reported [90].

#### 3.4.7. 1-(4-Methoxyphenyl)-7-azaindole (**1b**)

The general procedure 1 (reaction time: 6 d), 2 (24 h), 3 (6 d), 4 (24 h) and 5 (25 min) using 4-iodoanisole (0.23, 0.26, 0.28, 0.23 and 0.26 g, respectively) afforded (eluent: petroleum ether-EtOAc 70:30) the title product in 0%, 50%, 54%, 60% and 35% yield, respectively, as a yellow solid. Mp 56–58 °C, IR: 670, 717, 764, 772, 796, 828, 894, 957, 1031, 1069, 1108, 1146, 1180, 1212, 1244, 1271, 1298, 1324, 1357, 1422, 1441, 1463, 1478, 1515, 1569, 1393, 1731, 1875, 2046, 2835, 2956, 3048. ^1^H-NMR (CDCl_3_): 3.81 (s, 3H, OMe), 6.58 (d, 1H, *J* = 3.6 Hz, H3), 7.03–7.06 (m, 2H, H3′ and H5′), 7.10 (dd, 1H, *J* = 7.8 et 4.7 Hz, H5), 7.42 (d, 1H, *J* = 3.6 Hz, H2), 7.59–7.62 (m, 2H, H2′ and H6′), 7.94 (dd, 1H, *J* = 7.8 et 1.6 Hz, H4), 8.40 (dd, 1H, *J* = 4.7 and 1.6 Hz, H6). ^13^C-NMR (CDCl_3_): 55.4 (CH_3_, OMe), 100.9 (CH, C3), 114.4 (2CH, C3′ and C5′), 116.3 (CH, C5), 121.1 (C, Cb), 125.4 (2CH, C2′ and C6′), 128.1 (CH, C2), 128.9 (CH, C4), 131.4 (C, C1′), 143.4 (CH, C6), 147.4 (C, Ca), 157.9 (C, C4′). The NMR data are as reported [90].

#### 3.4.8. 1-(4-Chlorophenyl)-7-azaindole (**1c**)

The general procedure 4 (24 h) using 1-chloro-4-iodobenzene (0.24 g) afforded (eluent: heptane-EtOAc 80:20) the title product in 65% yield as a white solid. Mp 92 °C, IR: 716, 769, 793, 821, 894, 954, 1095, 1148, 1210, 1237, 1267, 1279, 1326, 1360, 1421, 1497, 1521, 1592, 1724, 1891, 3054, 3075, 3113. ^1^H-NMR (CDCl_3_): 6.63 (d, 1H, *J* = 3.7 Hz, H3), 7.14 (dd, 1H, *J* = 7.8 and 4.7 Hz, H5), 7.46 (d, *J* = 3.8 Hz, 1H, H2), 7.46–7.50 (m, 2H, Ph), 7.71–7.76 (m, 2H, Ph), 7.96 (dd, 1H, *J* = 7.8 and 1.6 Hz, H4), 8.38 (dd, 1H, *J* = 4.7 and 1.6 Hz, H6). ^13^C-NMR (75 MHz, CDCl_3_) δ 102.2 (CH, C3), 117.0 (CH, C5), 121.7 (C, Cb), 125.0 (2CH, Ph), 127.4 (CH, C2), 129.3 (CH, C4), 129.5 (2CH, Ph), 131.8 (C, C1′ or C4′), 137.1 (C, C1′ or C4′), 143.8 (CH, C6), 147.5 (C, Ca). These data are as reported [91].

#### 3.4.9. 1-(4-Fluorophenyl)-7-azaindole (**1d**)

The general procedure 4 (24 h) using 1-fluoro-4-iodobenzene (0.12 mL) afforded (eluent: petroleum ether-EtOAc 90:10) the title product in 80% yield as a greenish oil. IR: 672, 717, 765, 772, 796, 816, 832, 894, 956, 1014, 1040, 1069, 1096, 1111, 1146, 1158, 1219, 1269, 1287, 1325, 1358, 1424, 1507, 1515, 1574, 1592, 1724, 1883, 3050. ^1^H-NMR (CDCl_3_): 6.62 (d, 1H, *J* = 3.6 Hz, H3), 7.13 (dd, 1H, *J* = 7.8 and 4.7 Hz, H5), 7.17–7.25 (m, 2H, Ph), 7.44 (d, 1H, *J* = 3.6 Hz, H2), 7.67–7.74 (m, 2H, Ph), 7.97 (dd, 1H, *J* = 7.8 and 1.6 Hz, H4), 8.38 (dd, 1H, *J* = 4.7 and 1.6 Hz, H6). ^13^C-NMR (75 MHz, CDCl_3_): 101.8 (CH, C3), 116.2 (d, 2CH, *J* = 22.8 Hz, C3′ and C5′), 116.8 (CH, C5), 121.5 (C, Cb), 125.8 (d, 2CH, *J* = 8.3 Hz, C2′ and C6′), 127.8 (CH, C2), 129.2 (CH, C4), 134.6 (d, C, *J* = 3.0 Hz, C1′), 143.7 (CH, C6), 147.6 (C, Ca), 161.0 (d, C, *J* = 246 Hz, C4′, C-F). These data are as reported [66].

#### 3.4.10. 1-(4-(Trifluoromethyl)phenyl)-7-azaindole (**1e**)

The general procedure 3 (reaction time: 24 h) and 4 (24 h) using 1-iodo-4-(trifluoromethyl)benzene (0.18 and 0.15 mL, respectively) afforded (eluent: petroleum ether-EtOAc 90:10) the title product in 95% and 65% yield, respectively, as a yellow solid. Mp 66–68 °C, IR: 706, 727, 764, 771, 796, 832, 895, 960, 1018, 1043, 1065, 1080, 1109, 1166, 1209, 1240, 1267, 1322, 1361, 1425, 1478, 1529, 1572, 1595, 1617, 1889, 3069. ^1^H-NMR (CDCl_3_): 6.68 (d, 1H, *J* = 3.7 Hz, H3), 7.18 (dd, 1H, *J* = 7.8 and 4.7 Hz, H5), 7.54 (d, 1H, *J* = 3.7 Hz, H2), 7.77 (d, 2H, *J* = 8.4 Hz, Ph), 7.96–8.01 (m, 3H, H4 and Ph), 8.39 (dd, 1H, *J* = 4.7 and 1.5 Hz, H6). ^13^C-NMR (CDCl_3_): 103.0 (CH, C3), 117.4 (CH, C5), 122.0 (C, Cb), 123.4 (2CH, C2′ and C6′), 124.2 (q, C, *J* = 272 Hz, CF_3_), 126.7 (q, 2CH, *J* = 3.7 Hz, C3′ and C5′), 127.1 (CH, C2), 127.9 (q, C, *J* = 33 Hz, C4′), 129.5 (CH, C4), 141.5 (C, C1′), 143.9 (CH, C6), 147.6 (C, Ca). The NMR data are close to those reported [92]. *Crystal data for* **1e**. C_14_H_9_F_3_N_2_, *M* = 262.23, *T* = 150(2) K, monoclinic, *P* 2_1_/*c*, *a* = 13.0804(9), *b* = 6.9738(5), *c* = 13.7561(8) Å, *β* = 111.263(2)°, *V* = 1169.41(13) Å^3^, *Z* = 4, *d* = 1.489 g cm^−3^, *μ* = 0.123 mm^−1^. A final refinement on *F*^2^ with 2648 unique intensities and 203 parameters converged at ω*R*(*F*^2^) = 0.1172 (*R*(*F*) = 0.0464) for 2105 observed reflections with *I* > 2σ(*I*). CCDC 2109688. *Other crystal data for* **1e**. C_14_H_9_F_3_N_2_, *M* = 262.23, *T* = 150(2) K, monoclinic, *P* 2_1_/*c*, *a* = 13.154(5), *b* = 7.248(3), *c* = 13.796(5) Å, *β* = 117.791(12)°, *V* = 1163.7(7) Å^3^, *Z* = 4, *d* = 1.497 g cm^−3^, *μ* = 0.124 mm^−1^. A final refinement on *F*^2^ with 2660 unique intensities and 182 parameters converged at ω*R*(*F*^2^) = 0.1833 (*R*(*F*) = 0.0916) for 1477 observed reflections with *I* > 2σ(*I*). CCDC 2109687.

#### 3.4.11. 1-(3,5-Dimethylphenyl)-7-azaindole (**1f**)

The general procedure 3 (reaction time: 24 h) using 1-iodo-3,5-dimethylbenzene (0.17 mL) afforded (eluent: petroleum ether-EtOAc 90:10) the title product in 97% yield as a yellow oil. IR: 689, 717, 763, 772, 795, 814, 844, 893, 1037, 1083, 1138, 1205, 1261, 1281, 1330, 1359, 1414, 1479, 1512, 1591, 1611, 2917, 3014. ^1^H-NMR (CDCl_3_): 2.42 (d, 6H, *J* = 0.5 Hz, Me), 6.60 (d, 1H, *J* = 3.6 Hz, H3), 7.00 (br s, 1H, H4′), 7.12 (dd, 1H, *J* = 7.8 and 4.7 Hz, H5), 7.36 (br s, 2H, H2′ and H6′), 7.48 (d, 1H, *J* = 3.6 Hz, H2), 7.96 (dd, 1H, *J* = 7.8 and 1.6 Hz, H4), 8.40 (dd, 1H, *J* = 4.7 and 1.6 Hz, H6). ^13^C-NMR (CDCl_3_): 21.5 (2CH_3_, Me), 101.2 (CH, C3), 116.5 (CH, C5), 121.5 (C, Cb), 122.2 (2CH, C2′ and C6′), 128.3 (2CH, C2 and C4′), 129.0 (CH, C4), 138.4 (C, C1′), 139.1 (2C, C3′ and C5′), 143.6 (CH, C6), 147.7 (C, Ca). These data are close to those reported [93].

#### 3.4.12. 1-(2-Thienyl)-7-azaindole (**1g**)

The general procedure 2 (reaction time: 24 h) and 3 (48 h) using 2-iodothiophene (0.12 and 0.13 mL, respectively) afforded (eluent: heptane-EtOAc 90:10) the title product in 37% and 92% yield, respectively, as a yellow oil. ^1^H-NMR (CDCl_3_): 6.60 (d, 1H, *J* = 3.7 Hz, H3), 7.03 (dd, 1H, *J* = 5.5 and 3.7 Hz, H4′), 7.12–7.16 (m, 2H, H5 and H5′), 7.28 (dd, 1H, *J* = 3.7 and 1.4 Hz, H3′), 7.49 (d, 1H, *J* = 3.7 Hz, H2), 7.93 (dd, 1H, *J* = 7.8 and 1.6 Hz, H4), 8.43 (dd, 1H, *J* = 4.7 and 1.6 Hz, H6). ^13^C-NMR (CDCl_3_): 102.3 (CH, C3), 117.1 (CH, C5 or C3′), 117.8 (CH, C5 or C3′), 120.6 (CH), 121.4 (C, Cb), 125.7 (CH), 128.2 (CH), 129.2 (CH, C4), 139.8 (C, C2′), 143.9 (CH, C6), 147.4 (C, Ca). The NMR data are as reported [74].

#### 3.4.13. 1-(3-Pyridyl)-7-azaindole (**1h**)

The general procedure 3 (reaction time: 48 h) and 4 (24 h) using 3-iodopyridine (0.25 and 0.205 g, respectively) afforded (eluent: heptane-EtOAc 70:30) the title product in 80% yield as a white solid. Mp 70–72 °C, IR: 705, 721, 766, 774, 799, 894, 955, 1023, 1043, 1075, 1112, 1150, 1190, 1212, 1243, 1272, 1329, 1365, 1419, 1438, 1488, 1517, 1584, 1897, 2160, 3046, 3429. ^1^H-NMR (CDCl_3_): 6.65 (d, 1H, *J* = 3.7 Hz, H3), 7.14 (dd, 1H, *J* = 7.9 and 4.7 Hz, H5), 7.43 (ddd, 1H, *J* = 8.3, 4.8 and 0.6 Hz, H5′), 7.50 (d, 1H, *J* = 3.7 Hz, H2), 7.95 (dd, 1H, *J* = 7.9 and 1.6 Hz, H4), 8.26 (ddd, 1H, *J* = 8.3, 2.6 and 1.5 Hz, H4′), 8.35 (dd, 1H, *J* = 4.7 and 1.6 Hz, H6), 8.55 (dd, 1H, *J* = 4.7 and 1.4 Hz, H6′), 8.98 (d, 1H, *J* = 2.4 Hz, H2′). ^13^C-NMR (CDCl_3_): 102.8 (CH, C3), 117.3 (CH, C5), 121.7 (C, Cb), 123.8 (CH, C5′), 126.9 (CH, C2), 129.4 (CH, C4), 131.0 (CH, C4′), 135.2 (C, C3′), 143.8 (CH, C6), 144.6 (CH, C2′ or C6′), 147.1 (CH, C2′ or C6′), 147.5 (C, Ca). The NMR data are as reported [94].

#### 3.4.14. 1-(2-Pyridyl)-7-azaindole (**1i**)

The general procedure 3 (reaction time: 48 h) and 4 (24 h) using 2-bromopyridine (110 and 95 μL, respectively) afforded, after recrystallization from heptane, the title product in 75% and 35% yield, respectively, as a white solid. Mp 66–68 °C, IR: 723, 765, 776, 799, 894, 969, 1050, 1071, 1141, 1208, 1238, 1264, 1303, 1322, 1367, 1417, 1440, 1468, 1477, 1518, 1587, 3014, 3054, 3107, 3151. ^1^H-NMR (CDCl_3_): 6.64 (d, 1H, *J* = 3.9 Hz, H3), 7.15 (ddd, 1H, *J* = 7.0, 4.9 and 1.0 Hz, H5′), 7.16 (dd, 1H, *J* = 7.8 and 4.8 Hz, H5), 7.86 (ddd, 1H, *J* = 8.4, 7.4 and 2.0 Hz, H4′), 7.95 (dd, 1H, *J* = 7.8 and 1.6 Hz, H4), 8.38 (d, 1H, *J* = 3.9 Hz, H2), 8.40 (dd, 1H, *J* = 4.8 and 1.6 Hz, H6), 8.49 (ddd, 1H, *J* = 4.8, 1.8 and 0.7 Hz, H6′), 8.92 (d, 1H, *J* = 8.4 Hz, H3′). ^13^C-NMR (CDCl_3_): 102.7 (CH, C3), 115.9 (CH, C3′), 117.3 (CH, C5), 120.5 (CH, C5′), 123.5 (C, Cb), 126.6 (CH, C2), 129.2 (CH, C4), 138.4 (CH, C4′), 143.2 (CH, C6), 147.7 (C, Ca), 148.4 (CH, C6′), 150.9 (C, C2′). The NMR data are as reported [95]. *Crystal data for* **1i**. C_12_H_9_N_3_, *M* = 195.22, *T* = 150(2) K, monoclinic, *P* 2_1_/*c*, *a* = 13.861(2), *b* = 8.2717(10), *c* = 16.721(3) Å, *β* = 104.205(5)°, *V* = 1858.5(5) Å^3^, *Z* = 8, *d* = 1.395 g cm^−3^, *μ* = 0.087 mm^−1^. A final refinement on *F*^2^ with 4242 unique intensities and 271 parameters converged at ω*R*(*F*^2^) = 0.1142 (*R*(*F*) = 0.0611) for 2520 observed reflections with *I* > 2σ(*I*). CCDC 2109689.

#### 3.4.15. 1-(4-Pyridyl)-7-azaindole (**1j**)

The general procedure 4 (reaction time: 24 h) using 4-bromopyridine (0.16 g) afforded (eluent: petroleum heptane-EtOAc-Et_3_N 79:20:1) the title product in 35% yield as a yellow solid. Mp 90 °C, IR: 709, 765, 775, 798, 821, 893, 994, 1112, 1151, 1199, 1222, 1246, 1268, 1331, 1366, 1420, 1474, 1505, 1521, 1587, 2853, 2925, 3033, 3105. ^1^H-NMR (300 MHz, CDCl_3_) δ 6.70 (d, 1H, *J* = 3.8 Hz, H3), 7.19 (dd, 1H, *J* = 7.8 and 4.7 Hz, H5), 7.62 (d, 1H, *J* = 3.8 Hz, H2), 7.96 (dd, 1H, *J* = 7.8 and 1.6 Hz, H4), 8.01 (d, 2H, *J* = 6.2 Hz, H3′ and H5′), 8.41 (dd, 1H, *J* = 4.7 and 1.6 Hz, H6), 8.70 (br s, 2H, H2′ and H6′). ^13^C-NMR (75 MHz, CDCl_3_) δ 104.3 (CH, C3), 116.2 (2CH, C3′ and C5′), 117.9 (CH, C5), 122.7 (C, Cb), 125.7 (CH, C2), 129.6 (CH, C4), 144.0 (CH, C6), 145.8 (C, C4′), 148.0 (C, Ca), 150.6 (2CH, C2′ and C6′). The NMR data are as reported [30]. *Crystal data for* **1j**. C_12_H_9_N_3_, *M* = 195.22, *T* = 150(2) K, orthorhombic, *P* 2_1_ 2_1_ 2_1_, *a* = 6.8590(4), *b* = 10.3076(6), *c* = 13.2404(6) Å, *V* = 936.09(9) Å^3^, *Z* = 4, *d* = 1.385 g cm^−3^, *μ* = 0.086 mm^−1^. A final refinement on *F*^2^ with 2133 unique intensities and 137 parameters converged at ω*R*(*F*^2^) = 0.0774 (*R*(*F*) = 0.0315) for 2018 observed reflections with *I* > 2σ(*I*). CCDC 2109690.

#### 3.4.16. 1,1′-(1,3-Phenylene)bis(7-azaindole) (**2k**)

The general procedure 3 (reaction time: 4 d) and 4 (5 d) using 1,3-diiodobenzene (0.20 and 0.16 g, respectively) afforded (eluent: heptane-EtOAc 90:10) the title product in 40 and 15% yield, respectively, as a white solid. Mp 110 °C, IR: 688, 719, 750, 774, 796, 874, 894, 1111, 1136, 1155, 1210, 1224, 1247, 1273, 1290, 1316, 1331, 1354, 1418, 1467, 1483, 1494, 1514, 1591, 1608, 1728, 2930, 3015, 3046, 3100. ^1^H-NMR (CDCl_3_): 6.63 (d, 2H, *J* = 3.7 Hz, H3), 7.13 (dd, 2H, *J* = 7.8 and 4.7 Hz, H5), 7.59 (d, 2H, *J* = 3.7 Hz, H2), 7.64 (dd, 1H, *J* = 8.8 and 7.3 Hz, H5′), 7.76–7.79 (m, 2H, H4′ and H6′), 7.96 (dd, 2H, *J* = 7.8 and 1.6 Hz, H4), 8.33 (t, 1H, *J* = 2.0 Hz, H2′), 8.40 (dd, 2H, *J* = 4.7 and 1.6 Hz, H6). ^13^C-NMR (CDCl_3_): 102.0 (2CH, C3), 116.9 (2CH, C5), 119.2 (CH), 121.3 (2CH, C4′ and C6′), 121.8 (2C, Cb), 127.7 (2CH, C2), 129.1 (2CH, C4), 130.1 (CH), 139.4 (2C, C1′ and C3′), 143.6 (2CH, C6), 147.6 (2C, Ca). These data are as reported [96]. *Crystal data for* **2k**. C_20_H_14_N_4_, *M* = 310.35, *T* = 295 K, orthorhombic, *P* 2_1_ 2_1_ 2_1_, *a* = 7.4379(5), *b* = 9.7143(7), *c* = 21.0654(12) Å, *V* = 1522.06(17) Å^3^, *Z* = 4, *d* = 1.354 g cm^−3^, *μ* = 0.083 mm^−1^. A final refinement on *F*^2^ with 3431 unique intensities and 217 parameters converged at ω*R*(*F*^2^) = 0.1108 (*R*(*F*) = 0.0447) for 2976 observed reflections with *I* > 2σ(*I*). CCDC 2109691.

#### 3.4.17. 1-(3-Iodophenyl)-7-azaindole (**2k′**)

It was obtained in the above reaction in 20% yield by using the general procedure 3 or 4, as a yellow oil. IR: 658, 682, 717, 730, 772, 796, 870, 893, 963, 994, 1040, 1060, 1075, 1091, 1111, 1148, 1170, 1209, 1235, 1262, 1278, 1323, 1354, 1414, 1431, 1479, 1514, 1583, 1660, 1727, 1860, 1925, 2415, 2474, 2563, 2853, 2924, 3017, 3053, 3099. ^1^H-NMR (CDCl_3_): 6.61 (d, 1H, *J* = 3.7 Hz, H3), 7.13 (dd, 1H, *J* = 7.8 and 4.7 Hz, H5), 7.20 (t, 1H, *J* = 8.0 Hz, H5′), 7.44 (d, 1H, *J* = 3.7 Hz, H2), 7.64 (ddd, 1H, *J* = 7.9, 1.5 and 1.0 Hz, H4′ or H6′), 7.81 (ddd, 1H, *J* = 8.1, 2.1 and 0.9 Hz, H4′ or H6′), 7.94 (dd, 1H, *J* = 7.8 and 1.6 Hz, H4), 8.15 (t, 1H, *J* = 1.9 Hz, H2′), 8.39 (dd, 1H, *J* = 4.7 and 1.6 Hz, H6). ^13^C-NMR (CDCl_3_): 94.3 (C, C3′, C-I), 102.3 (CH, C3), 117.0 (CH, C5), 121.6 (C, Cb), 123.0 (CH), 127.3 (CH), 129.2 (CH, C4), 130.7 (CH), 132.4 (CH), 135.1 (CH), 139.5 (C, C1′), 143.7 (CH, C6), 147.4 (C, Ca). Anal. Calc. for C_13_H_9_IN_2_ (320.13): C 48.77, H 2.83, N 8.75. Found: C 48.46, H 3.11, N 8.83.

#### 3.4.18. 1,1′-(1,4-Phenylene)bis(7-azaindole) (**2l**)

In Ref. [97], The general procedure 1 (reaction time: 5 d) and 3 (reaction time: 3 d) using 1,4-diiodobenzene (0.16 and 0.20 g, respectively) afforded (eluent: petroleum ether-EtOAc 90:10) the title product in 0% and 80% yield, respectively, as a beige solid. Mp 194 °C, IR: 660, 715, 725, 762, 772, 794, 834, 893, 929, 953, 1045, 1072, 1114, 1131, 1155, 1200, 1240, 1264, 1290, 1324, 1341, 1358, 1416, 1474, 1520, 1573, 1594, 1727, 1890, 2570, 3049, 3078, 3102. ^1^H-NMR (300 MHz, CDCl_3_) δ 6.67 (d, 2H, *J* = 3.6 Hz, H3), 7.16 (dd, 2H, *J* = 7.8 and 4.7 Hz, H5), 7.56 (d, 2H, *J* = 3.6 Hz, H2), 7.92 (s, 4H, Ph), 8.00 (dd, 2H, *J* = 7.8 and 1.6 Hz, H4), 8.40 (dd, 2H, *J* = 4.7 and 1.6 Hz, H6). ^13^C-NMR (75 MHz, CDCl_3_) δ 102.0 (2CH, C3), 117.0 (2CH, C5), 121.7 (2C, Cb), 125.1 (4CH, Ph), 127.9 (2CH, C2), 129.3 (2CH, C4), 136.6 (2C, C1′), 143.8 (2CH, C6), 147.7 (2C, Ca). *Crystal data for* **2l**. C_20_H_14_N_4_, *M* = 310.35, *T* = 150(2) K, orthorhombic, *P c a b*, *a* = 8.9954(6), *b* = 9.9118(5), *c* = 16.4444(11) Å, *V* = 1466.19(16) Å^3^, *Z* = 4, *d* = 1.406 g cm^−3^, *μ* = 0.087 mm^−1^. A final refinement on *F*^2^ with 1683 unique intensities and 109 parameters converged at ω*R*(*F*^2^) = 0.0995 (*R*(*F*) = 0.0467) for 1192 observed reflections with *I* > 2σ(*I*). CCDC 2109692.

### 3.5. Deprotometalation-Iodolysis of the Different 1-Arylated 7-Azaindoles

#### 3.5.1. General Procedure 6

This was adapted from a reported protocol [52]. The base was prepared from LiTMP [in situ generated by adding *n*-BuLi (about 1.6 M hexanes solution, 3.0 mmol) to a stirred, cooled (0 °C) solution of 2,2,6,6-tetramethylpiperidine (0.50 mL, 3.0 mmol) in THF (5 mL)] and ZnCl_2_·TMEDA (0.26 g, 1.0 mmol), which was added 5 min later. The mixture was stirred for 15 min at 0 °C before introduction of the substrate (1.0 mmol) at 0–10 °C. After 2 h at rt, iodolysis was performed by adding a solution of I_2_ (0.76 g, 3.0 mmol) in THF (8 mL). The mixture was stirred overnight before addition of an aqueous saturated solution of Na_2_S_2_O_3_ (10 mL) and extraction with EtOAc (3 × 40 mL). The combined organic layers were dried over MgSO_4_ and concentrated under reduced pressure. Purification by chromatography on silica gel (the eluent is given in the product description) led to the iodinated derivative.

#### 3.5.2. 2-Iodo-1-phenyl-7-azaindole (**3a**)

The general procedure 6 from 1-phenyl-7-azaindole (**1a**; 0.19 g) afforded (eluent: heptane-Et_2_O 70:30) the title product in 75% yield as a white solid. Mp 120 °C, IR: 693, 755, 801, 967, 1109, 1268, 1307, 1339, 1413, 1451, 1498, 1567, 1589, 3054. ^1^H-NMR (CDCl_3_): 6.95 (s, 1H, H3), 7.07 (dd, 1H, *J* = 7.8 and 4.8 Hz, H5), 7.38–7.42 (m, 2H, Ph), 7.40–7.60 (m, 3H, Ph), 7.88 (dd, 1H, *J* = 7.8 and 1.6 Hz, H4), 8.23 (dd, 1H, *J* = 4.8 and 1.6 Hz, H6). ^13^C-NMR (CDCl_3_): 86.0 (C, C2, C-I), 111.9 (CH, C3), 116.9 (CH, C5), 122.2 (C, Cb), 127.4 (CH, C4′), 128.9 (CH, C4), 129.3 (2CH, Ph), 129.4 (2CH, Ph), 137.7 (C, C1′), 143.9 (CH, C6), 149.8 (C, Ca). *Crystal data for* **3a**. C_13_H_9_IN_2_, *M* = 320.12, *T* = 150(2) K, monoclinic, *P* 2_1_/*c*, *a* = 10.4501(4), *b* = 5.6326(3), *c* = 19.4738(8) Å, *β* = 94.859(2)°, *V* = 1142.13(9) Å^3^, *Z* = 4, *d* = 1.862 g cm^−3^, *μ* = 2.775 mm^−1^. A final refinement on *F*^2^ with 2626 unique intensities and 145 parameters converged at ω*R*(*F*^2^) = 0.0453 (*R*(*F*) = 0.0232) for 2269 observed reflections with *I* > 2σ(*I*). CCDC 2109693.

#### 3.5.3. 2-Iodo-1-(2-iodophenyl)-7-azaindole (**3a′**)

It was obtained in the above reaction in 5% yield as a yellow solid. IR: 694, 728, 758, 783, 801, 906, 1109, 1267, 1306, 1339, 1358, 1407, 1456, 1496, 1573, 1588, 1604, 2224, 2926, 3058. ^1^H-NMR (CDCl_3_): 6.99 (s, 1H, H3), 7.08 (dd, 1H, *J* = 7.9 and 4.8 Hz, H5), 7.26 (td, 1H, *J* = 7.7 and 1.7 Hz, H4′ or H5′), 7.37 (dd, 1H, *J* = 7.8 and 1.6 Hz, H3′ or H6′), 7.56 (td, 1H, *J* = 7.6 and 1.4 Hz, H4′ or H5′), 7.90 (dd, 1H, *J* = 7.8 and 1.6 Hz, H4), 8.03 (dd, 1H, *J* = 8.0 and 1.3 Hz, H3′ or H6′), 8.24 (dd, 1H, *J* = 4.8 and 1.6 Hz, H6). ^13^C-NMR (CDCl_3_): 85.8 (C, C2, C-I), 101.1 (C, C2′, C-I), 111.8 (CH, C3), 117.0 (CH, C5), 122.3 (C, Cb), 127.6 (CH), 129.4 (CH, C4), 131.2 (CH), 131.4 (CH), 140.0 (CH, C3′), 141.0 (C, C1′), 144.2 (CH, C6), 149.6 (C, Ca). *Crystal data for* **3a′**. C_13_H_8_I_2_N_2_, *M* = 446.01, *T* = 150(2) K, monoclinic, *P* 2_1_/*n*, *a* = 7.2949(12), *b* = 14.380(2), *c* = 12.247(2) Å, *β* = 91.159(8)°, *V* = 1284.4(4) Å^3^, *Z* = 4, *d* = 2.307 g cm^−3^, *μ* = 4.875 mm^−1^. A final refinement on *F*^2^ with 2940 unique intensities and 154 parameters converged at ω*R*(*F*^2^) = 0.0667 (*R*(*F*) = 0.0293) for 2603 observed reflections with *I* > 2σ(*I*). CCDC 2109694.

#### 3.5.4. 2-Iodo-1-(4-(trifluoromethyl)phenyl)-7-azaindole (**3e**)

The general procedure 6 from 1-(4-(trifluoromethyl)phenyl)-7-azaindole (**1e**; 0.22 g) afforded (eluent: petroleum ether-EtOAc 90:10) the title product in 10% yield as a white solid. Mp 88–90 °C. IR: 709, 759, 772, 798, 845, 909, 967, 1019, 1064, 1103, 1122, 1164,1214, 1262, 1320, 1353, 1411, 1456, 1493, 1521, 1570, 1590, 1615, 2963. ^1^H-NMR (CDCl_3_): 7.00 (s, 1H, H3), 7.10 (dd, 1H, *J* = 7.9 and 4.8 Hz, H5), 7.59 (d, 2H, *J* = 8.2 Hz, Ph), 7.83 (d, 2H, *J* = 8.3 Hz, Ph), 7.90 (dd, 1H, *J* = 7.9 and 1.6 Hz, H4), 8.23 (dd, 1H, *J* = 4.8 and 1.6 Hz, H6). ^13^C-NMR (CDCl_3_): 84.5 (C, C2, C-I), 113.2 (CH, C3), 117.5 (CH, C5), 122.2 (C, Cb), 124.0 (q, C, *J* = 272 Hz, CF_3_), 126.5 (q, 2CH, *J* = 3.7 Hz, C3′ and C5′), 127.8 (CH, C4), 129.7 (2CH, C2′ and C6′), 130.7 (q, C, *J* = 32.8 Hz, C4′), 140.6 (C, C1′), 144.1 (CH, C6), 149.7 (C, Ca). Anal. Calc. for C_14_H_8_F_3_IN_2_ (388.13): C 43.32, H 2.08, N 7.22. Found: C 43.58, H 2.15, N 7.12.

#### 3.5.5. 2-Iodo-1-(3-iodo-4-(trifluoromethyl)phenyl)-7-azaindole (**3e′**)

It was obtained in the above reaction in 20% yield as a greenish oil. IR: 715, 759, 803, 840, 893, 969, 1075, 1133, 1172, 1268, 1318, 1418, 1457, 1498, 1590. ^1^H-NMR (CDCl_3_): 7.01 (s, 1H, H3), 7.11 (dd, 1H, *J* = 7.9 and 4.8 Hz, H5), 7.48 (dd, 1H, *J* = 8.2 and 0.4 Hz, H6′), 7.82 (ddd, 1H, *J* = 8.2, 2.0 and 0.6 Hz, H5′), 7.92 (dd, 1H, *J* = 7.9 and 1.6 Hz, H4), 8.23 (dd, 1H, *J* = 4.8 and 1.6 Hz, H6), 8.27 (d, 1H, *J* = 1.4 Hz, H3′). ^13^C-NMR (CDCl_3_): 84.5 (C, C2, C-I), 101.2 (C, C2′, C-I), 112.6 (CH, C3), 117.4 (CH, C5), 122.4 (C, Cb), 122.7 (q, C, *J* = 273 Hz, CF_3_), 126.5 (q, CH, *J* = 3.5 Hz, C5′), 127.8 (CH, C4), 131.8 (CH, C6′), 133.0 (q, C, *J* = 33.3 Hz, C4′), 137.0 (q, CH, *J* = 3.7 Hz, C3′), 144.3 (CH, C6), 144.4 (C, d, *J* = 1.2 Hz, C1′), 149.6 (C, Ca). Anal. Calc. for C_14_H_7_F_3_I_2_N_2_ (514.03): C 32.71, H 1.37, N 5.45. Found: C 32.83, H 1.25, N 5.20.

#### 3.5.6. 1-(3,5-Dimethylphenyl)-2-iodo-7-azaindole (**3f**)

The general procedure 6 from 1-(3,4-dimethylphenyl)-7-azaindole (**1f**; 0.22 g) afforded (eluent: petroleum ether-EtOAc 90:10) the title product in 40% yield as a greenish oil. IR: 695, 770, 801, 852, 1038, 1109, 1133, 1175, 1273, 1310, 1339, 1380, 1408, 1457, 1490, 1569, 1588, 2922. ^1^H-NMR (CDCl_3_): 2.41 (d, 6H, *J* = 0.5 Hz, Me), 6.92 (s, 1H, H3), 6.99 (br s, 2H, H2′ and H6′), 7.04 (dd, 1H, *J* = 7.8 and 4.8 Hz, H5), 7.14 (br s, 1H, H4′), 7.86 (dd, 1H, *J* = 7.8 and 1.6 Hz, H4), 8.23 (dd, 1H, *J* = 4.7 and 1.6 Hz, H6). ^13^C-NMR (CDCl_3_): 21.5 (2CH_3_, Me), 86.5 (C, C2, C-I), 111.5 (CH, C3), 116.7 (CH, C5), 122.2 (C, Cb), 127.1 (2CH, C2′ and C6′), 127.3 (CH, C4 or C4′), 130.9 (CH, C4 or C4′), 137.6 (C, C1′), 139.0 (2C, C3′ and C5′), 143.9 (CH, C6), 149.9 (C, Ca). Anal. Calc. for C_15_H_13_IN_2_ (348.18): C 51.74, H 3.76, N 8.05. Found: C 51.54, H 4.17, N 8.12.

#### 3.5.7. 1-(5-Iodo-2-thienyl)-7-azaindole (**3g**)

The general procedure 6 from 1-(2-thienyl)-7-azaindole (**1g**; 0.20 g) afforded (eluent: heptane-EtOAc 90:10) the title product in 30% yield as a white solid. Mp 86 °C. IR: 676, 713, 765, 793, 893, 942, 1052, 1133, 1192, 1235, 1270, 1304, 1328, 1354, 1416, 1454, 1473, 1510, 1547, 1577, 1593, 2922, 3052, 3071. ^1^H-NMR (CDCl_3_): 6.59 (d, 1H, *J* = 3.7 Hz, H3), 6.86 (d, 1H, *J* = 3.9 Hz, H3′ or H4′), 7.14 (dd, 1H, *J* = 7.7 and 4.6 Hz, H5), 7.17 (d, 1H, *J* = 3.9 Hz, H3′ or H4′), 7.43 (d, 1H, *J* = 3.7 Hz, H2), 7.91 (dd, 1H, *J* = 7.8 and 1.4 Hz, H4), 8.40 (dd, 1H, *J* = 4.6 and 1.3 Hz, H6). ^13^C-NMR (CDCl_3_): 68.4 (C, C5′, C-I), 102.9 (CH, C3), 117.3 (CH, C5 or C3′), 117.6 (CH, C5 or C3′), 121.2 (C, Cb), 127.1 (CH, C2), 129.3 (CH, C4), 134.9 (CH, C4′), 143.8 (CH, C6), 144.2 (C, C2′), 146.9 (C, Ca). *Crystal data for* **3g**. C_11_H_7_IN_2_S, *M* = 326.15, *T* = 295(2) K, orthorhombic, *P* 2_1_ 2_1_ 2_1_, *a* = 4.1711(7), *b* = 11.5731(17), *c* = 22.327(3) Å, *V* = 1077.8(3) Å^3^, *Z* = 4, *d* = 2.010 g cm^−3^, *μ* = 3.129 mm^−1^. A final refinement on *F*^2^ with 2450 unique intensities and 137 parameters converged at ω*R*(*F*^2^) = 0.1127 (*R*(*F*) = 0.0498) for 1939 observed reflections with *I* > 2σ(*I*). CCDC 2109695.

#### 3.5.8. 2-Iodo-1-(5-iodo-2-thienyl)-7-azaindole (**3g′**)

It was obtained in the above reaction in 40% yield as a yellow solid. Mp 152–154 °C. IR: 675, 735, 759, 790, 802, 897, 914, 946, 1044, 1108, 1176, 1208, 1260, 1292, 1309, 1337, 1400, 1434, 1456, 1493, 1550,1573, 1590, 3059. ^1^H-NMR (CDCl_3_): 6.83 (d, 1H, *J* = 3.9 Hz, H3′ or H4′), 6.95 (s, 1H, H3), 7.10 (dd, 1H, *J* = 7.8 and 4.8 Hz, H5), 7.34 (d, 1H, *J* = 3.9 Hz, H3′ or H4′), 7.86 (dd, 1H, *J* = 7.8 and 1.6 Hz, H4), 8.27 (dd, 1H, *J* = 4.8 and 1.6 Hz, H6). ^13^C-NMR (CDCl_3_): 74.5 (C, C5′, C-I), 87.7 (C, C2, C-I), 112.8 (CH, C3), 117.4 (CH, C5), 122.1 (C, Cb), 127.5 (CH, C3′), 129.4 (CH, C4), 135.5 (CH, C4′), 142.3 (C, C2′), 144.0 (CH, C6), 150.1 (C, Ca). *Crystal data for* **3g′**. C_11_H_6_I_2_N_2_S, *M* = 452.04, *T* = 150(2) K, triclinic, *P* -1, *a* = 5.4889(3), *b* = 10.8313(6), *c* = 10.9361(6) Å, α = 84.984(3), β = 77.554(3), γ = 78.838(3)°, *V* = 622.17(6) Å^3^, *Z* = 2, *d* = 2.413 g cm^−3^, *μ* = 5.195 mm^−1^. A final refinement on *F*^2^ with 2822 unique intensities and 145 parameters converged at ω*R*(*F*^2^) = 0.0529 (*R*(*F*) = 0.0225) for 2610 observed reflections with *I* > 2σ(*I*). CCDC 2109696.

#### 3.5.9. 2-Iodo-1-(3-(7-aza-1-indolyl)phenyl)-7-azaindole (**3k**)

The general procedure 6 from 1,1′-(1,3-phenylene)bis(7-azaindole) (**2k**; 0.31 g) afforded (eluent: heptane-EtOAc 70:30) the title product in about 20% yield. It was identified by NMR: ^1^H-NMR (CDCl_3_): 6.64 (d, *J* = 3.7 Hz, 1H, H3′), 6.98 (s, 1H, H3), 7.08 (dd, 1H, *J* = 7.9 and 4.8 Hz, H5), 7.14 (dd, 1H, *J* = 7.8 and 4.7 Hz, H5′), 7.39 (ddd, 1H, *J* = 7.9, 2.0 and 1.0 Hz, H4″ or H6″), 7.61 (d, 1H, *J* = 3.7 Hz, H2′), 7.71 (t, 1H, *J* = 8.1 Hz, H5″), 7.85 (t, 1H, *J* = 2.0 Hz, H2″), 7.89 (dd, 1H *J* = 7.9 and 1.6 Hz, H4), 7.96 (dd, 1H, *J* = 7.8 and 1.6 Hz, H4′), 8.13 (ddd, 1H, *J* = 8.2, 2.1 and 1.0 Hz, H4″ or H6″), 8.24 (dd, 1H, *J* = 4.7 and 1.6 Hz, H6), 8.38 (dd, 1H, *J* = 4.7 and 1.6 Hz, H6′). Anal. Calc. for C_20_H_13_IN_4_ (436.26): C 55.06, H 3.00, N 12.84. Found: C 55.17, H 3.08, N 12.33.

#### 3.5.10. 1,1′-(1,3-Phenylene)bis(2-iodo-7-azaindole) (**3k′**)

It was obtained in the above reaction in 20% yield as a beige solid. Mp 182 °C. IR: 693, 727, 757, 782, 801, 905, 958, 1005, 1040, 1084, 1108, 1173, 1208, 1266, 1306, 1338, 1357, 1406, 1455, 1495, 1572, 1588, 1604, 1725, 1859, 2223, 2551, 2602, 2853, 2926, 3057, 3120, 3420, 3742, 3868. ^1^H-NMR (CDCl_3_): 6.95 (s, 2H, H3), 7.07 (dd, 2H, *J* = 7.8 and 4.8 Hz, H5), 7.49 (t, 1H, *J* = 1.9 Hz, H2′), 7.59–7.62 (m, 2H, H4′ and H6′), 7.75 (dd, 1H, *J* = 8.6 and 7.2 Hz, H5′), 7.86 (dd, 2H, *J* = 7.8 and 1.6 Hz, H4), 8.23 (dd, 2H, *J* = 4.7 and 1.6 Hz, H6). ^13^C-NMR (CDCl_3_): 85.5 (2C, C2, C-I), 112.7 (2CH, C3), 117.3 (2CH, C5), 122.4 (2C, Cb), 127.5 (2CH, C4′ and C6′), 129.5 (2CH, C4), 129.7 (CH, C2′ or C5′), 130.3 (CH, C2′ or C5′), 138.4 (2C, C1′), 144.0 (2CH, C6), 150.0 (2C, Ca). *Crystal data for* **3k′**. C_20_H_12_I_2_N_4_, *M* = 562.14, *T* = 150(2) K, monoclinic, *C* 2/*c*, *a* = 14.5928(9), *b* = 16.9523(10), *c* = 8.4985(6) Å, *β* = 123.070(3)°, *V* = 1761.8(2) Å^3^, *Z* = 4, *d* = 2.119 g cm^−3^, *μ* = 3.582 mm^−1^. A final refinement on *F*^2^ with 2019 unique intensities and 119 parameters converged at ω*R*(*F*^2^) = 0.0639 (*R*(*F*) = 0.0281) for 1797 observed reflections with *I* > 2σ(*I*). CCDC 2109697.

#### 3.5.11. 1,1′-(1-Iodo-2,4-phenylene)bis(2-iodo-7-azaindole (**3k″**)

It was obtained in the above reaction in 14% yield as a white solid. Mp 132 °C. IR: 679, 757, 820, 1006, 1025, 1053, 1270, 1410, 1478, 1621, 2123, 2251, 3465. ^1^H-NMR ((CD_3_)_2_SO): 7.09 (s, 2H, H3), 7.15 (dt, 2H, *J* = 7.8 and 4.7 Hz, H5), 7.47 (dd, 1H, *J* = 8.4 and 2.4 Hz, H5′), 7.52 (d, 1H, *J* = 2.4 Hz, H3′), 8.00 (dt, 2H, *J* = 7.8 and 1.5 Hz, H4), 8.14 (dd, 1H, *J* = 4.7 and 1.6 Hz, H6), 8.17 (dd, 1H, *J* = 4.7 and 1.6 Hz, H6), 8.26 (d, 1H, *J* = 8.4 Hz, H6′). ^13^C-NMR ((CD_3_)_2_SO): 88.0 (C, C2, C-I), 88.7 (C, C2, C-I), 101.9 (C, C1′, C-I), 111.2 (CH, C3), 112.3 (CH, C3), 116.9 (CH, C5), 117.3 (CH, C5), 121.8 (C, Cb), 121.8 (C, Cb), 127.4 (CH, C3′ or C5′), 127.6 (CH, C3′ or C5′), 131.6 (CH, C4), 131.8 (CH, C4), 137.9 (C, C4′), 139.3 (CH, C6′), 141.3 (C, C2′), 143.2 (CH, C6), 143.3 (CH, C6), 149.1 (C, Ca), 149.2 (C, Ca). Anal. Calc. for C_20_H_11_I_3_N_4_ (688.05): C 34.91, H 1.61, N 8.14. Found: C 34.66, H 1.79, N 8.13.

### 3.6. Direct Iodination of the Different 1-Arylated 7-Azaindoles

#### 3.6.1. General Procedure 7

This was inspired from a reported protocol [66]. A mixture of I_2_ (0.28 g, 1.1 mmol), KOH (0.17 g, 3.0 mmol) and 7-azaindole (1.0 mmol) in acetonitrile (5 mL) was stirred for 12 h at rt. An aqueous saturated solution of Na_2_S_2_O_3_ (10 mL) was then added before extraction with EtOAc (3 × 40 mL). The combined organic layers were dried over MgSO_4_ and concentrated under reduced pressure. Purification by chromatography on silica gel (the eluent is given in the product description) led to the iodinated derivative.

#### 3.6.2. General Procedure 8

This was inspired from a reported protocol [66]. A mixture of I_2_ (0.51 g, 2.0 mmol), KOH (0.17 g, 3.0 mmol) and 7-azaindole (1.0 mmol) in acetonitrile (5 mL) was stirred at 40 °C for 14 h. An aqueous saturated solution of Na_2_S_2_O_3_ (10 mL) was then added before extraction with EtOAc (3 × 40 mL). The combined organic layers were dried over MgSO_4_ and concentrated under reduced pressure. Purification by chromatography on silica gel (the eluent is given in the product description) led to the iodinated derivative.

#### 3.6.3. 3-Iodo-1-phenyl-7-azaindole (**4a**)

The general procedure 7 from 1-phenyl-7-azaindole (**1a**; 0.19 g) afforded (eluent: heptane-EtOAc 90:10) the title product in 65% yield as a yellow oil. IR: 690, 727, 753, 763, 790, 924, 979, 1039, 1074, 1110, 1143, 1221, 1270, 1313, 1348, 1409, 1455, 1479, 1497, 1510, 1561, 1591, 3043. ^1^H-NMR (CDCl_3_): 7.21 (dd, 1H, *J* = 7.9 and 4.7 Hz, H5), 7.35 (tt, 1H, *J* = 7.4 and 1.2 Hz, H4′), 7.48–7.55 (m, 2H, Ph), 7.61 (s, 1H, H2), 7.69–7.73 (m, 2H, Ph), 7.78 (dd, 1H, *J* = 7.9 and 1.6 Hz, H4), 8.39 (dd, 1H, *J* = 4.7 and 1.5 Hz, H6). ^13^C-NMR (CDCl_3_): 56.6 (C, C3, C-I), 117.6 (CH, C5), 124.0 (2CH, C2′ and C6′), 124.0 (C, Cb), 126.9 (CH, C4′), 129.5 (2CH, C3′ and C5′), 129.6 (CH, C4), 131.9 (CH, C2), 137.7 (C, C1′), 144.8 (CH, C6), 147.1 (C, Ca). These data are as reported [66].

#### 3.6.4. 3-Iodo-1-(4-methoxyphenyl)-7-azaindole (**4b**)

The general procedure 7 from 1-(4-methoxyphenyl)-7-azaindole (**1b**; 0.22 g) afforded (eluent: heptane-EtOAc 90:10) the title product in 40% yield as a white solid. Mp 58 °C, IR: 700, 764, 799, 830, 924, 979, 1035, 1109, 1144, 1180,1220, 1247, 1272, 1299, 1319, 1348, 1413, 1440, 1462, 1478, 1514, 1560, 1591, 2834, 2931, 3004, 3044. ^1^H-NMR (CDCl_3_): 3.85 (s, 3H, OMe), 7.00–7.05 (m, 2H, H3′ and H5′), 7.18 (dd, 1H, *J* = 7.9 and 4.7 Hz, H5), 7.54 (s, 1H, H2), 7.53–7.58 (m, 2H, H2′ and H6′), 7.77 (dd, 1H, *J* = 7.9 and 1.6 Hz, H4), 8.36 (dd, 1H, *J* = 4.7 and 1.5 Hz, H6). ^13^C-NMR (CDCl_3_): 55.7 (CH_3_, OMe), 55.7 (C, C3, C-I), 114.7 (2CH, C3′ and C5′), 117.4 (CH, C5), 123.7 (C, Cb), 125.8 (2CH, C2′ and C6′), 129.5 (CH, C4), 130.8 (C, C1′), 132.3 (CH, C2), 144.7 (CH, C6), 147.3 (C, Ca), 158.6 (C, C4′). Anal. Calc. for C_14_H_11_IN_2_O (350.16): C 48.02, H 3.17, N 8.00. Found: C 48.08, H 3.47, N 7.76.

#### 3.6.5. 1-(4-Chlorophenyl)-3-iodo-7-azaindole (**4c**)

The general procedure 8 from 1-(4-chlorophenyl)-7-azaindole (**1c**; 0.23 g) afforded (eluent: heptane-Et_2_O 80:20) the title product in 40% yield as a white solid. Mp 110 °C, IR: 673, 705, 747, 762, 792, 827, 924, 978, 1014, 1039, 1092, 1110, 1144, 1191, 1221, 1266, 1278, 1313, 1338, 1351, 1406, 1490, 1509, 1560, 1590, 1722, 1889, 2418, 2628, 2855, 2924, 3014, 3125, 3096, 3044. ^1^H-NMR (CDCl_3_): 7.21 (dd, 1H, *J* = 7.9 and 4.7 Hz, H5), 7.45–7.50 (m, 2H, Ph), 7.57 (s, 1H, H2), 7.65–7.69 (m, 2H, Ph), 7.77 (dd, 1H, *J* = 7.9 and 1.6 Hz, H4), 8.37 (dd, 1H, *J* = 4.7 and 1.5 Hz, H6). ^13^C-NMR (CDCl_3_). 55.2 (C, C3, C-I), 117.9 (CH, C5), 124.1 (C, Cb), 125.0 (2CH, Ph), 129.6 (2CH, Ph), 129.8 (CH, C4), 131.4 (CH, C2), 132.4 (C, C1′ or C4′), 136.3 (C, C1′ or C4′), 144.9 (CH, C6), 147.1 (C, Ca). *Crystal data for* **4c**. C_13_H_8_ClIN_2_, *M* = 354.56, *T* = 150(2) K, monoclinic, *C*, *a* = 4.0519(5), *b* = 25.673(3), *c* = 11.5697(15) Å, *β* = 93.736(4)°, *V* = 1201.0(3) Å^3^, *Z* = 4, *d* = 1.961 g cm^−3^, *μ* = 2.865 mm^−1^. A final refinement on *F*^2^ with 2645 unique intensities and 154 parameters converged at ω*R*(*F*^2^) = 0.0591 (*R*(*F*) = 0.0237) for 2622 observed reflections with *I* > 2σ(*I*). CCDC 2109698.

#### 3.6.6. 1-(4-Fluorophenyl)-3-iodo-7-azaindole (**4d**)

The general procedure 7 from 1-(4-fluorophenyl)-7-azaindole (**1d**; 0.21 g) afforded (eluent: heptane-EtOAc 90:10) the title product in 52% yield as a white solid. Mp 98 °C, IR: 702, 764, 794, 818, 834, 925, 980, 1111, 1158, 1215, 1269, 1319, 1413, 1479, 1514, 1561, 1592, 2923. ^1^H-NMR (CDCl_3_): 7.18–7.25 (m, 3H, H5 and Ph), 7.63–7.70 (m, 2H, Ph), 7.57 (s, 1H, H2), 7.80 (dd, 1H, *J* = 7.9 and 1.6 Hz, H4), 8.38 (dd, 1H, *J* = 4.7 and 1.5 Hz, H6). ^13^C-NMR (CDCl_3_): 56.6 (C, C3, C-I), 116.5 (d, 2CH, *J* = 23 Hz, C3′ and C5′), 117.8 (CH, C5), 124.0 (C, Cb), 126.0 (d, 2CH, *J* = 8.4 Hz, C2′ and C6′), 129.8 (CH, C4), 131.9 (CH, C2), 133.9 (d, C, *J* = 3.0 Hz, C1′), 145.0 (CH, C6), 147.3 (C, Ca), 161.4 (d, C, *J* = 247 Hz, C4′, C-F). Anal. Calc. for C_13_H_8_FIN_2_ (338.12): C 46.18, H 2.38, N 8.29. Found: C 46.32, H 2.12, N 8.11.

#### 3.6.7. 3-Iodo-1-(4-(trifluoromethyl)phenyl)-7-azaindole (**4e**)

The general procedure 7 and 8 from 1-(4-(trifluoromethyl)phenyl)-7-azaindole (**1e**; 0.26 g) afforded (eluent: heptane-EtOAc 90:10) the title product in 33 and 45% yield, respectively, as a white solid. Mp 78–80 °C, IR: 454, 762, 794, 841, 924, 982, 1016, 1068, 1111, 1124, 1168, 1224, 1269, 1319, 1411, 1475, 1527, 1567, 1596, 1617, 1729, 2856, 2925, 2956, 3052, 3133. ^1^H-NMR (CDCl_3_): 7.23 (dd, 1H, *J* = 7.9 and 4.7 Hz, H5), 7.63 (s, 1H, H2), 7.76 (d, 2H, *J* = 8.5 Hz, Ph), 7.78 (dd, 1H, *J* = 7.9 and 1.6 Hz, H4), 7.91 (d, 2H, *J* = 8.4 Hz, Ph), 8.39 (dd, 1H, *J* = 4.7 and 1.5 Hz, H6). ^13^C-NMR (CDCl_3_): 58.3 (C, C3, C-I), 118.2 (CH, C5), 123.4 (2CH, C2′ and C6′), 124.0 (d, C, *J* = 272 Hz, CF_3_), 124.4 (C, Cb), 126.7 (q, 2CH, *J* = 3.8 Hz, C3′ and C5′), 128.4 (d, C, *J* = 32.8 Hz, C4′), 130.0 (CH, C4), 131.0 (CH, C2), 140.6 (C, C1′), 145.0 (CH, C6), 147.1 (C, Ca). *Crystal data for* **4e**. C_14_H_8_F_3_IN_2_, *M* = 388.12, *T* = 150(2) K, monoclinic, *P* 2_1_/*n*, *a* = 19.7915(9), *b* = 7.5240(4), *c* = 19.8815(11) Å, *β* = 117.242(2)°, *V* = 2632.2(2) Å^3^, *Z* = 8, *d* = 1.959 g cm^−3^, *μ* = 2.459 mm^−1^. A final refinement on *F*^2^ with 6006 unique intensities and 357 parameters converged at ω*R*(*F*^2^) = 0.0809 (*R*(*F*) = 0.0391) for 4927 observed reflections with *I* > 2σ(*I*). CCDC 2109699.

#### 3.6.8. 3-Iodo-1-(3,5-dimethylphenyl)-7-azaindole (**4f**)

The general procedure 7 from 1-(3,5-dimethylphenyl)-7-azaindole (**1f**; 0.22 g) afforded (eluent: hexane-CHCl_3_ 60:40) the title product in 62% yield as a white solid. Mp 80 °C, IR: 664, 689, 762, 789, 824, 845, 891, 934, 965, 998, 1037, 1056, 1080, 1109, 1137, 1188, 1210, 1258, 1281, 1314, 1322, 1340, 1361, 1378, 1404, 1433, 1465, 1491, 1508, 1562, 1590, 1611, 1727, 1918, 2853, 2919, 2952, 3010, 3042, 3123, 3629. ^1^H-NMR (CDCl_3_): 2.40 (d, 6H, *J* = 0.5 Hz, Me), 7.01 (br s, 1H, H4′), 7.20 (dd, 1H, *J* = 7.9 and 4.7 Hz, H5), 7.29 (br s, 2H, H2′ and H6′), 7.58 (s, 1H, H2), 7.78 (dd, 1H, *J* = 7.9 and 1.6 Hz, H4), 8.38 (dd, 1H, *J* = 4.7 and 1.5 Hz, H6). ^13^C-NMR (CDCl_3_): 21.5 (2CH_3_, Me), 56.0 (C, C3, C-I), 117.5 (CH, C5), 122.3 (2CH, C2′ and C6′), 124.0 (C, Cb), 128.9 (CH, C4′), 129.6 (CH, C4), 132.4 (CH, C2), 137.7 (C, C1′), 139.3 (2C, C3′ and C5′), 144.8 (CH, C6), 147.4 (C, Ca). Anal. Calc. for C_15_H_13_IN_2_ (348.19): C 51.74, H 3.76, N 8.05. Found: C 51.43, H 3.44, N 7.88.

#### 3.6.9. 1-(5-Iodo-2-thienyl)-7-azaindole (**3g**)

The general procedure 7 from 1-(2-thienyl)-7-azaindole (**1g**; 0.20 g) afforded (eluent: hexane-CHCl_3_ 70:30) the title product in 35% yield as a white solid. Its analyses were found identical to those reported in Section 3.4.7.

#### 3.6.10. 3-Iodo-1-(3-pyridyl)-7-azaindole (**4h**)

The general procedure 7 (but at 40 °C) and 8 (but for 10 h) from 1-(3-pyridyl)-7-azaindole (**1h**; 0.20 g) afforded (eluent: heptane-EtOAc 80:20) the title product in 36% and 45% yield, respectively, as a yellow solid. Mp 114 °C, IR: 703, 739, 763, 796, 924, 978, 1023, 1045, 1110, 1145, 1186, 1208, 1227, 1271, 1311, 1320, 1353, 1406, 1431, 1484, 1510, 1560, 1583, 1718, 1899, 2139, 2853, 2922, 3043, 3356, 3650. ^1^H-NMR (CDCl_3_): 7.23 (dd, 1H, *J* = 7.9 and 4.7 Hz, H5), 7.45 (dd, 1H, *J* = 8.2 and 4.8 Hz, H5′), 7.63 (s, 1H, H2), 7.78 (dd, 1H, *J* = 7.9 and 1.5 Hz, H4), 8.20 (ddd, 1H, *J* = 8.2, 2.5 and 1.5 Hz, H4′), 8.37 (dd, 1H, *J* = 4.7 and 1.5 Hz, H6), 8.59 (dd, 1H, *J* = 4.7 and 1.1 Hz, H6′), 8.97 (d, 1H, *J* = 2.3 Hz, H2′). ^13^C-NMR (CDCl_3_): 58.0 (C, C3, C-I), 118.1 (CH, C5), 123.9 (CH, C5′), 124.2 (C, Cb), 130.0 (CH, C4), 130.8 (CH, C2), 131.1 (CH, C4′), 134.5 (C, C3′), 144.6 (CH, C6), 145.1 (CH, C2′ or C6′), 147.1 (C, Ca), 147.7 (CH, C2′ or C6′). *Crystal data for* **4h**. C_12_H_8_IN_3_, *M* = 321.11, *T* = 150(2) K, triclinic, *P* −1, *a* = 3.9819(2), *b* = 8.8450(5), *c* = 16.4714(9) Å, *α* = 74.841(2), *β* = 83.779(2), *γ* = 78.265(2)°, *V* = 547.30(5) Å^3^, *Z* = 2, *d* = 1.949 g cm^−3^, *μ* = 2.899 mm^−1^. A final refinement on *F*^2^ with 2497 unique intensities and 145 parameters converged at ω*R*(*F*^2^) = 0.0453 (*R*(*F*) = 0.0195) for 2375 observed reflections with *I* > 2σ(*I*). CCDC 2109700.

#### 3.6.11. 3-Iodo-1-(2-pyridyl)-7-azaindole (**4i**)

The general procedure 7 and 8 from 1-(2-pyridyl)-7-azaindole (**1i**; 0.20 g) afforded (eluent: CHCl_3_-hexane 60:40) the title product in 17% and 51% yield, respectively, as a yellow solid. Mp 108 °C, IR: 739, 764, 775, 823, 884, 929, 962, 983, 1000, 1050, 1079, 1096, 1109, 1133, 1151, 1188, 1219, 1262, 1302, 1352, 1362, 1404, 1436, 1466, 1478, 1511, 1562, 1587, 1611, 1724, 1860, 1919, 2626, 2850, 2920, 2953, 3011, 3058, 3107, 3145, 3628, 3735. ^1^H-NMR (CDCl_3_): 7.15 (ddd, 1H, *J* = 7.3, 4.9 and 0.8 Hz, H5′), 7.22 (dd, 1H, *J* = 7.9 and 4.8 Hz, H5), 7.75 (dd, 1H, *J* = 7.9 and 1.6 Hz, H4), 7.84 (ddd, 1H, *J* = 8.4, 7.4 and 1.9 Hz, H4′), 8.39 (dd, 1H, *J* = 4.7 and 1.4 Hz, H6), 8.45 (dd, 1H, *J* = 4.8 and 1.1 Hz, H6′), 8.52 (s, 1H, H2), 8.84 (d, 1H, *J* = 8.4 Hz, H3′). ^13^C-NMR (CDCl_3_): 58.9 (C, C3, C-I), 115.6 (CH, C3′), 118.0 (CH, C5), 120.9 (CH, C5′), 125.6 (C, Cb), 129.7 (CH, C4), 130.8 (CH, C2), 138.4 (CH, C4′), 144.3 (CH, C6), 147.0 (C, Ca), 148.3 (CH, C6′), 150.1 (C, C2′). *Crystal data for* **4i**. C_12_H_8_IN_3_, *M* = 321.11, *T* = 150(2) K, monoclinic, *P* 2_1_/*n*, *a* = 7.7719(12), *b* = 8.1656(12), *c* = 17.328(2) Å, *β* = 90.723(5)°, *V* = 1099.6(3) Å^3^, *Z* = 4, *d* = 1.940 g cm^−3^, *μ* = 2.885 mm^−1^. A final refinement on *F*^2^ with 2518 unique intensities and 145 parameters converged at ω*R*(*F*^2^) = 0.0647 (*R*(*F*) = 0.0285) for 2275 observed reflections with *I* > 2σ(*I*). CCDC 2109701.

### 3.7. N-Arylation of Azoles by Using 1-Arylated 3-Iodo-7-azaindoles

#### 3.7.1. 3-(1-Indolyl)-1-(4-(trifluoromethyl)phenyl)-7-azaindole (**5e**)

A mixture of 3-iodo-1-(4-(trifluoromethyl)phenyl)-7-azaindole (**4e**; 0.39 g, 1.0 mmol), indole (0.12 g, 1.0 mmol), Cu_2_O (7 mg, 50 μmol) and Cs_2_CO_3_ (0.65 g, 2.0 mmol) in DMSO (1 mL) was heated at 110 °C under argon for 24 h. The reaction mixture was cooled to rt. The residue was taken with EtOAc (20 mL) and filtrated over celite. Removal of the solvent under reduced pressure and purification of the crude over silica gel (eluent: heptane-EtOAc 80:20) afforded the title product in 30% yield as a colorless oil. IR: 424, 447, 561, 593, 621, 655, 664, 712, 726, 746, 758, 766, 801, 842, 881, 904, 934, 949, 962, 1016, 1066, 1105, 1129, 1162, 1188, 1210, 1240, 1268, 1322, 1349, 1365, 1389, 1420, 1455, 1472, 1482, 1521, 1565, 1582, 1595, 1614, 1724, 2852, 2923, 2955, 3049. ^1^H-NMR (CDCl_3_): 6.57 (dd, 1H, *J* = 3.4 and 0.8 Hz, H3′), 6.75 (s, 1H, H2), 6.92 (d, 1H, *J* = 3.4 Hz, H2′), 7.19 (dd, 1H, *J* = 5.6 and 1.4 Hz), 7.22 (dd, 1H, *J* = 5.9 and 1.4 Hz), 7.21 (dd, 1H, *J* = 7.8 and 4.8 Hz, H5), 7.35 (d, 2H, *J* = 8.3 Hz, Ph), 7.41 (d, 1H, *J* = 8.0 Hz), 7.56 (d, 2H, *J* = 8.4 Hz, Ph), 7.62–7.65 (m, 1H), 8.04 (dd, 1H, *J* = 7.8 and 1.6 Hz, H4), 8.41 (dd, 1H, *J* = 4.8 and 1.6 Hz, H6). ^13^C-NMR (CDCl_3_): 98.1 (CH), 102.8 (C), 105.3 (CH), 110.7 (CH), 118.3 (CH, C5), 120.1 (C), 121.3 (CH), 121.4 (CH), 123.4 (CH), 126.5 (q, 2CH, *J* = 3.5 Hz, C3” and C5”), 126.8 (2CH, C2” and C6”), 128.4 (CH, C4), 128.7 (C), 129.2 (CH), 134.4 (C), 137.5 (C), 138.3 (C), 144.5 (CH, C6); C4” and CF_3_ not seen. Anal. Calc. for C_22_H_14_F_3_N_3_ (377.37): C 70.02, H 3.74, N 11.14. Found: C 70.15, H 3.69, N 11.15.

#### 3.7.2. General Procedure 9 Using Copper(I) Iodide with Ligand

A mixture of the iodinated (1.0 mmol) or diiodinated (0.50 mmol) 7-azaindole, indole (0.14 g, 1.2 mmol), CuI (9.5 mg, 50 μmol), DMEDA (11 μL, 0.10 mmol) and K_3_PO_4_ (0.42 g, 2.0 mmol) in DMF (1 mL) was degassed and heated at reflux under argon (the reaction time is given in the product description). The reaction mixture was cooled to rt. The residue was taken with EtOAc (20 mL) and filtrated over celite. Removal of the solvent under reduced pressure and purification of the crude over silica gel (the eluent is given in the product description) gave the product.

#### 3.7.3. 3-(1-Indolyl)-1-(2-pyridyl)-7-azaindole (**5i**)

The general procedure 9 (reaction time: 24 h) from 3-iodo-1-(2-pyridyl)-7-azaindole (**4i**; 0.32 g) afforded (eluent: CH_2_Cl_2_-hexane 70:30) the title product in 50% yield as a white solid. Mp 136 °C, IR (ATR): 739, 762, 774, 907, 968, 997, 1010, 1052, 1134, 1213, 1230, 1267, 1303, 1294, 1340, 1423, 1440, 1469, 1513, 1575, 1590, 1606, 3022, 3052, 3110, 3133. ^1^H-NMR (CDCl_3_): 6.81 (dd, 1H, *J* = 3.2 and 0.9 Hz), 7.15–7.20 (m, 2H), 7.25–7.33 (m, 2H), 7.44 (d, 1H, *J* = 3.2 Hz), 7.51–7.54 (m, 1H), 7.80–7.91 (m, 3H), 8.52 (ddd, 2H, *J* = 7.3, 4.9 and 1.7 Hz), 8.67 (s, 1H), 9.07 (d, 1H, *J* = 8.4 Hz, H3″). ^13^C-NMR (CDCl_3_): 103.4 (CH), 110.8 (CH), 116.0 (CH, C3″), 117.4 (CH, C5), 117.5 (C), 119.0 (C), 120.4 (CH), 120.4 (CH), 120.6 (CH), 121.1 (CH), 122.4 (CH), 127.5 (C, Cb), 128.8 (CH), 128.9 (CH), 137.0 (C), 138.3 (CH, C4″), 144.3 (CH, C6), 145.9 (C, Ca), 148.2 (CH, C6″), 150.2 (C, C2″). *Crystal data for* **5i**. C_20_H_14_N_4_, *M* = 310.35, *T* = 150(2) K, triclinic, *P*-1, *a* = 7.0573(10), *b* = 8.3875(11), *c* = 13.563(2) Å, *α* = 87.146(5), *β* = 75.381(5), *γ* = 72.673(5)°, *V* = 741.31(19) Å^3^, *Z* = 2, *d* = 1.390 g cm^−3^, *μ* = 0.086 mm^−1^. A final refinement on *F*^2^ with 3361 unique intensities and 217 parameters converged at ω*R*(*F*^2^) = 0.0994 (*R*(*F*) = 0.0389) for 2635 observed reflections with *I* > 2σ(*I*). CCDC 2109702.

#### 3.7.4. 2-(1-Indolyl)-1-(5-(1-indolyl)-2-thienyl)-7-azaindole (**6g′**)

The general procedure 9 (reaction time: 24 h) from 2-iodo-1-(5-iodo-2-thienyl)-1*H*-pyrrolo[2,3-*b*]pyridine (**3g′**; 0.23 g) afforded (eluent: petroleum ether-EtOAc 80:20) the title product in 20% yield as a yellow oil. IR: 422, 659, 714, 737, 761, 801, 881, 902, 929, 965, 1012, 1043, 1071, 1106, 1127, 1207, 1266, 1303, 1316, 1336, 1347, 1387, 1412, 1450, 1474, 1504, 1517, 1568, 1594, 1713, 2923, 2960, 3050. ^1^H-NMR (CDCl_3_): 6.61 (dd, 1H, *J* = 3.3 and 0.9 Hz), 6.69 (dd, 1H, *J* = 3.3 and 0.9 Hz), 6.77 (s, 1H), 6.77 (d, 1H, *J* = 3.6 Hz), 6.80 (d, 1H, *J* = 4.0 Hz), 7.15–7.28 (m, 6H), 7.29 (dd, 1H, *J* = 7.9 and 4.9 Hz), 7.39 (t, 1H, *J* = 8.2 Hz), 7.62 (dd, 1H, *J* = 6.5 and 1.2 Hz), 7.68 (dd, 1H, *J* = 6.4 and 2.0 Hz), 8.04 (dd, 1H, *J* = 7.9 and 1.6 Hz), 8.51 (dd, 1H, *J* = 4.8 and 1.6 Hz). ^13^C-NMR (CDCl_3_): 98.7 (CH), 104.5 (CH), 105.2 (CH), 110.7 (CH), 110.7 (CH), 118.4 (CH), 118.9 (CH), 120.0 (C), 121.1 (CH), 121.1 (CH), 121.2 (CH), 121.3 (CH), 123.0 (CH), 123.2 (CH), 123.4 (CH), 128.7 (C), 128.8 (CH), 129.1 (CH), 129.1 (C), 129.3 (CH), 130.7 (C), 135.2 (C), 137.0 (C), 138.0 (C), 139.8 (C), 144.8 (CH, C6), 147.6 (C, Ca). Anal. Calc. for C_27_H_18_N_4_S (430.53): C 75.33, H 4.21, N 13.01. Found: C 75.11, H 4.46, N 12.65.

### 3.8. Evaluation of the Biological Properties

The antibacterial, antifungal and antioxidant activity was determined as described previously [77].

## 4. Conclusions

Our goal in the present paper was to rationalize the conversion of 1-aryl-7-azaindoles into either the corresponding 2-iodo derivatives (by deprotometalation-iodolysis) or the corresponding 3-iodo derivatives (by direct iodination). This could be achieved by calculating either the p*K*_a_ values or the HOMO orbital coefficients, respectively. The atomic charges also allowed the regioselectivity of these reactions to be predicted. Thus, the obtained iodides were converted into derivatives with promising biological properties.

## Figures and Tables

**Table 1:**
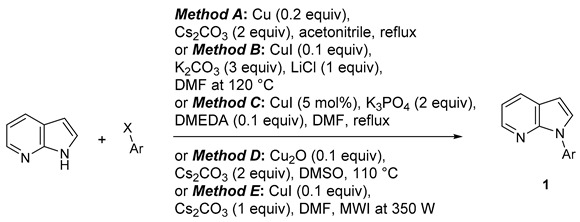
1-Arylation of 7-azaindole by using methods reported for the *N*-arylation of azoles.

Entry	Ar-X	*Method* (Reaction Time)	Product (E), Yield (%) ^1^
1 2 3 4 5	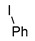	*A* (29 h) *B* (24 h) *C* (30 h) *D* (24 h) *E* (15 min)	**1a**, 20 **1a**, 15 **1a**, 75 **1a**, 75 **1a**, 18
6 7 8 9 10	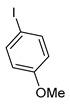	*A* (6 d) *B* (24 h) *C* (6 d) *D* (24 h) *E* (25 min)	**1b**, 0 **1b**, 50 **1b**, 54 **1b**, 60 **1b**, 35
11	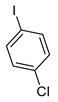	*D* (24 h)	**1c**, 65
12	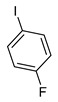	*D* (24 h)	**1d**, 80
13 14	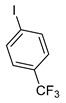	*C* (24 h) *D* (24 h)	**1e**, 95 **1e**, 65
15	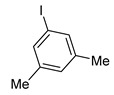	*C* (24 h)	**1f**, 97
16 17	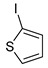	*B* (24 h) *C* (48 h)	**1g**, 37 **1g**, 92
18 19	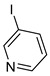	*C* (48 h) *D* (24 h)	**1h**, 80 **1h**, 80
20 21	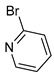	*C* (48 h) *D* (24 h)	**1i**, 75 **1i**, 35
22	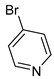	*D* (24 h)	**1j**, 35

^1^ After purification (see experimental part); when low yields were recorded, starting materials were in general recovered.

**Table 2:**
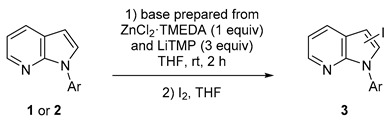
Deprotometalation of the 1-arylated 7-azaindoles followed by iodolysis.

Entry	Substrate		Product(s) (E), Yield(s) (%) ^1^	
1	** 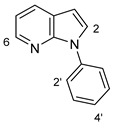 **	**1a**	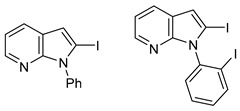	**3a**, 75; **3a′**, 5
2	** 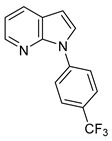 **	**1e**	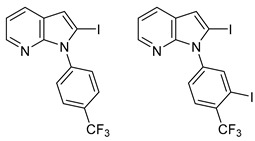	**3e**, 10; **3e′**, 20 ^2^
3	** 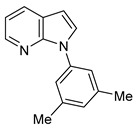 **	**1f**	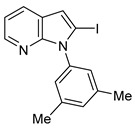	**3f**, 40 ^3^
4	** 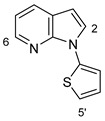 **	**1g**	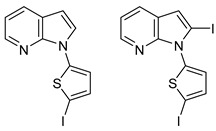	**3g**, 30; **3g′**, 40
5	** 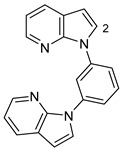 **	**2k**	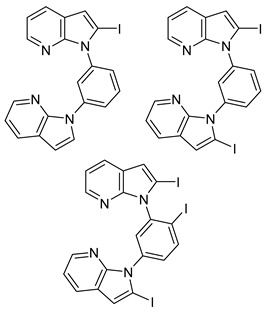	**3k**, 20; **3k′**, 20; ^2^ **3k″**, 14 ^2^

^1^ After purification (see experimental part). ^2^ Low yields were obtained, due to difficult separation. ^3^ The rest was **1f**.

**Table 3:**
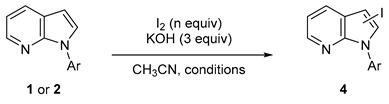
Direct iodination of the 1-arylated 7-azaindoles.

Entry	Substrate		n Equiv	Conditions	Product (E), Yield (%) ^1^	
**1**	** 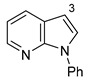 **	**1a**	1.1	rt, 12 h	** 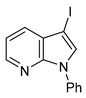 **	**4a**, 65
2	** 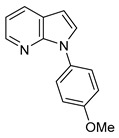 **	**1b**	1.1	rt, 12 h	** 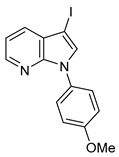 **	**4b**, 40
3	** 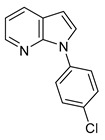 **	**1c**	2	40 °C, 14 h	** 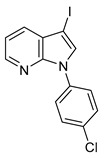 **	**4c**, 40
4	** 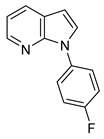 **	**1d**	1.1	rt, 12 h	** 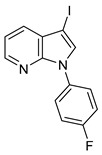 **	**4d**, 52
5 6	** 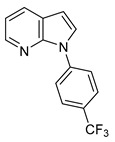 **	**1e**	1.1 2	rt, 12 h 40 °C, 14 h	** 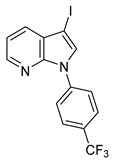 **	**4e**, 33 **4e**, 45
7	** 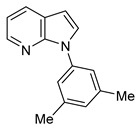 **	**1f**	1.1	rt, 12 h	** 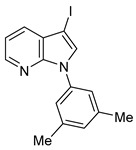 **	**4f**, 62
8	** 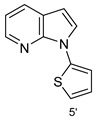 **	**1g**	1.1	rt, 12 h	** 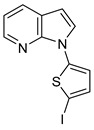 **	**3g, 35**
9 10	** 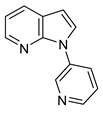 **	**1h**	1.1 2	40 °C, 12 h 40 °C, 10 h	** 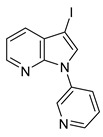 **	**4h**, 36 **4h**, 45
11 12	** 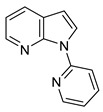 **	**1i**	1.1 2	rt, 12 h 40 °C, 14 h	** 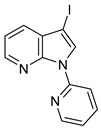 **	**4i**, 17 **4i**, 51

^1^ After purification (see experimental part); when low yields were recorded, starting materials were in general recovered.

**Table 4:**
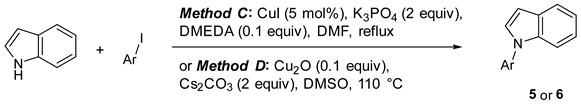
*N*-arylation of indole by a few of the prepared iodides.

Entry	Ar-I (n Equiv)			*Method* (Time)	Product (E), Yield (%) ^1^	
**1**	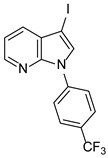	**4e**	(1)	*D* (24 h)	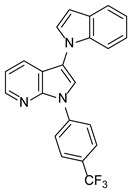	**5e**, 30 ^2^
2	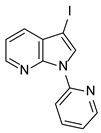	**4i**	(1)	*C* (24 h)	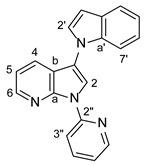	**5i**, 50 ^2^
3	** 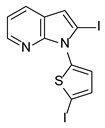 **	**3g′**	(0.5)	*C* (24 h)	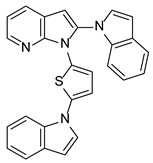	**6g′**, 20 ^3^

^1^ After purification (see experimental part). ^2^ The rest was mainly recovered starting material. ^3^ A low yield was obtained, due to difficult separation.

**Table 5:** Antimicrobial and antifungal activity ^1^ of some of the synthesized compounds.

Compound	Amount dissolved in DMSO	*Escherichia* *coli*	*Pseudomonas* *aeruginosa*	*Staphylococcus* *aureus*	*Enterococcus* *faecium*	*Listeria* *monocytogenes*	*Candida* *dubliniensis*	*Candida* *albicans*
**1a**	500 μg ^2^	6	5	8	5	±	**23**	-
**1b**	250 μg ^3^	0	0	0	11	0	**18**	-
**1c**	150 μg ^4^	0	0	0	0	0	-	0
**1e**	500 μg ^2^	±	0	0	0	0	0	-
**1f**	200 μg ^3^	0	0	0	11	0	13	-
**1h**	500 μg ^2^	10	8	11	0	10	**27**	-
**1i**	250 μg ^3^	0	0	±	13	0	**16**	-
**1j**	250 μg ^3^	0	0	0	15	**22**	10	-
**2k**	150 μg ^4^	0	0	0	0	0	-	0
**2l**	500 μg ^2^	0	0	0	0	0	0	-
**3a**	500 μg ^2^	0	0	0	0	0	13	-
**3g**	500 μg ^2^	±	0	0	0	0	0	-
**3g′**	500 μg ^2^	±	±	0	0	0	0	-
**5i**	500 μg ^2^	0	0	0	0	0	0	-
Reference compound		28 ^5^	28 ^5^	18 ^6^	24 ^6^	30 ^7^	10 ^8^	-

^1^ The diameters of zones of inhibition are given in mm. ^2^ 10 μL/well. ^3^ 50 μL/well. ^4^ 30 μL/well. ^5^ Ceftazidim (30 μg). ^6^ Vancomycin (30 μg). ^7^ Amoxicillin (25 μg). ^8^ Nystatin (416 UI).

**Table 6:** Antioxidant activity of some of the synthesized compounds.

Compound	RSA (%) ^1^ at t = 0 min	RSA (%) ^1^ at t = 30 min
**1b**	52	54
**1c**	98	58
**1f**	42	48
**1i**	44	46
**1j**	55	58
**5i**	51	53

^1^ Percentage of the radical scavenger activity (RSA) at a concentration of 5 μg/μL in DMSO at rt.

## Data Availability

All data are included in this paper.

## Supplementary Materials

The following data are available online. The NMR data of the compounds **2k**, **2k′**, **3a**, **3a′**, **3e–g**, **3g′**, **3k**, **3k′**, **3k”**, **4b–f**, **4h**, **4i**, **5e**, **5i** and **6g′**; the calculated values of the Gibbs energies Δ*G*_acid_ [kJ·mol^−1^] for deprotonation, the cartesian coordinates of molecular geometry for the most stable rotamer form optimized at the B3LYP/6-31G(d) level of theory in the .xyz format.

## Sample Availability

Samples of the compounds **1c**, **1e**, **1h**, **1j**, **2k** and **5i** are available from the authors. All iodides should be prepared just before use due to moderate stability.
