# Supplementary material for: Deprotometalation-Iodolysis and Direct Iodination of 1-Arylated 7-Azaindoles: Reactivity Studies and Molecule Properties
Source: Molecules. 2021 Oct 19;26(20):6314. doi: 10.3390/molecules26206314 (PMC8537530; doi:10.3390/molecules26206314)

Supplementary Materials

Deprotometalation-iodolysis and direct iodination of 1-arylated 7-azaindoles: Reactivity studies and molecule properties

Mohamed Yacine Ameur Messaoud [1,2], Ghenia Bentabed-Ababsa [2,*], Ziad Fajloun [3,4,*], Monzer Hamze [5], Yury S. Halauko [6,*], Oleg A. Ivashkevich [7], Vadim E. Matulis [7], Thierry Roisnel [1], Vincent Dorcet [1] and Florence Mongin [1,*]

[1] *Univ Rennes, CNRS, ISCR (Institut des Sciences Chimiques de Rennes) - UMR 6226, F-35000 Rennes, France*

[2] *Laboratoire de Synthèse Organique Appliquée, Faculté des Sciences Exactes et Appliquées, Université d'Oran 1 Ahmed Ben Bella, BP 1524 El M'Naouer, 31000 Oran, Algeria*

[3] *Laboratory of Applied Biotechnology (LBA3B), Azm Center for Research in Biotechnology and its Applications, EDST, Lebanese University, 1300 Tripoli, Lebanon*

[4] *Faculty of Sciences 3, Lebanese University, Campus Michel Slayman 1352 Ras Maska – Tripoli, Lebanon*

[5] *Laboratoire Microbiologie, Santé et Environnement (LMSE), Doctoral School of Sciences and Technology, Faculty of Public Health, Lebanese University, 1300 Tripoli, Lebanon*

[6] *UNESCO Chair of Belarusian State University, Minsk 220030, Belarus*

[7] *Research Institute for Physico-Chemical Problems of Belarusian State University, Minsk 220030, Belarus*

bentabedg@gmail.com (G. Bentabed-Ababsa); ziad.fajloun@ul.edu.lb (Z. Fajloun);

hys@tut.by (Y. S. Halauko); florence.mongin@univ-rennes1.fr (F. Mongin)

NMR data of compounds **2k**, **2k'**, **3a**, **3a'**, **3e-g**, **3g'**, **3k**, **3k'**, **3k"**, **4b-f**, **4h**, **4i**, **5e**, **5i** and **6g'**	p. SM2
Calculated values of the Gibbs energies ΔG_{acid} for deprotonation	p. SM33
Cartesian coordinates of molecular geometry for the most stable rotamer form optimized at the B3LYP/6-31G(d) level of theory	see separate file in the .xyz format

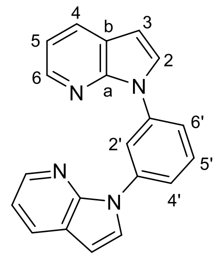

1,1'-(1,3-Phenylene)bis(1*H*-pyrrolo[2,3-*b*]pyridine) (2k)

^{1}H NMR (300 MHz, $CDCl_3$)

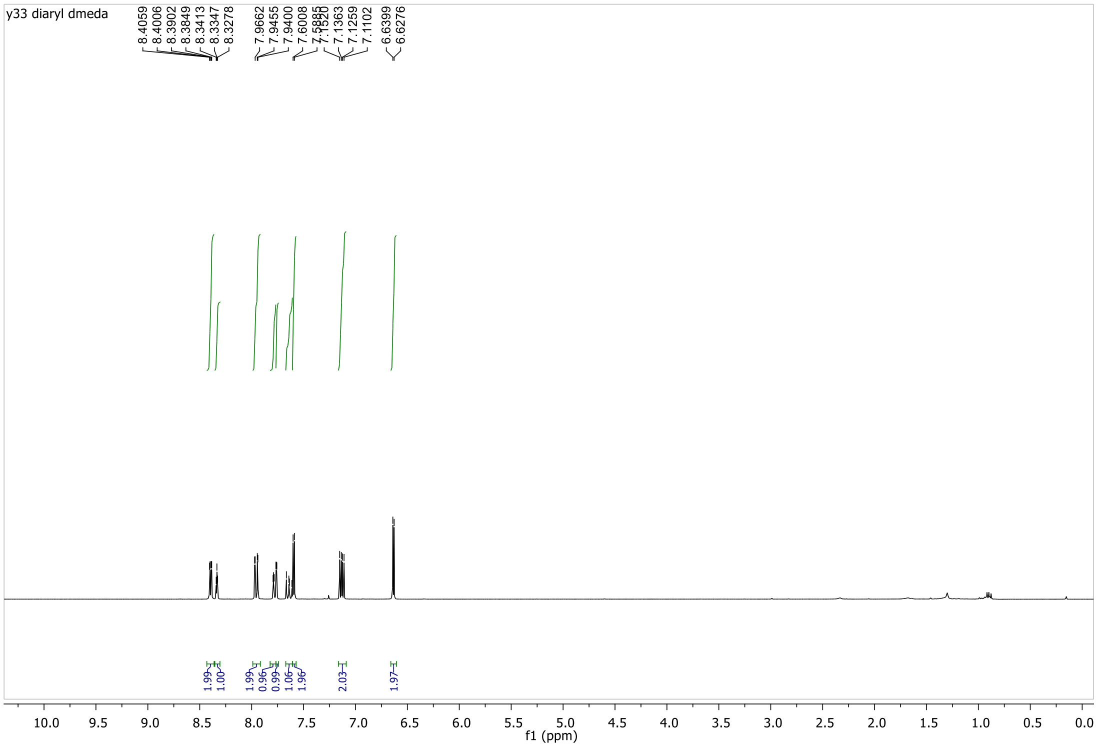

^{13}C NMR (75 MHz, $CDCl_3$)

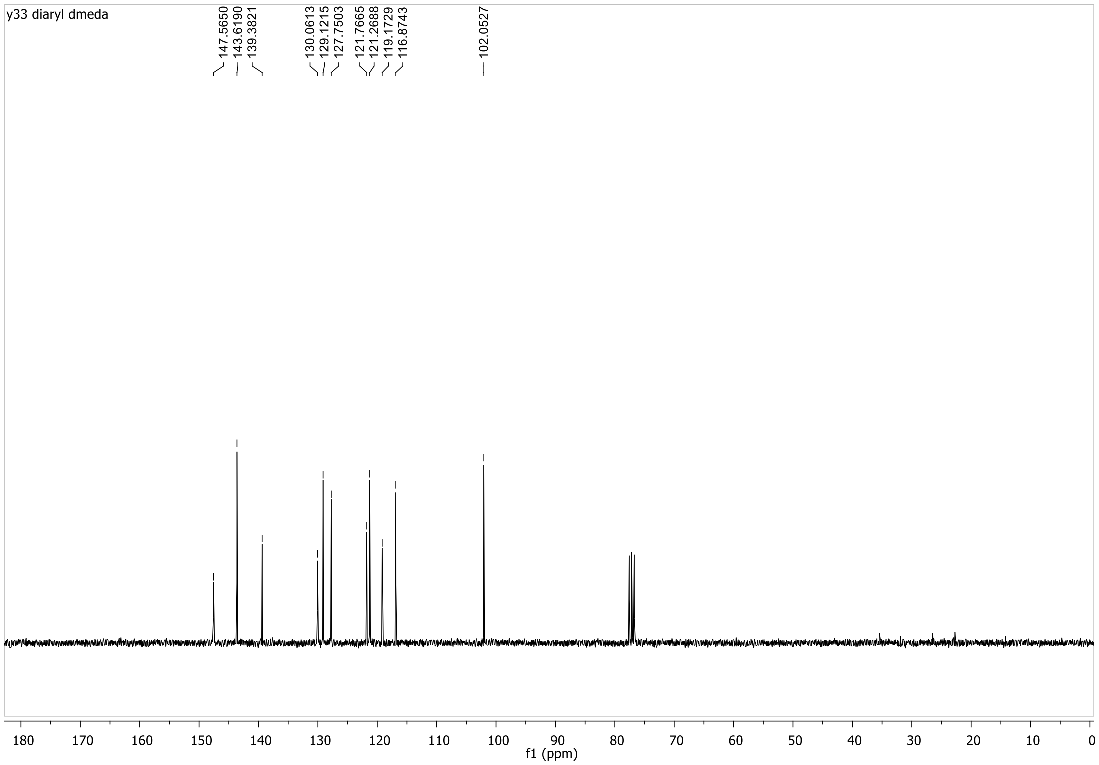

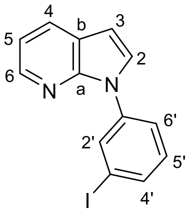

1-(3-Iodophenyl)-1*H*-pyrrolo[2,3-*b*]pyridine (2k')

^{1}H NMR (300 MHz, $CDCl_3$)

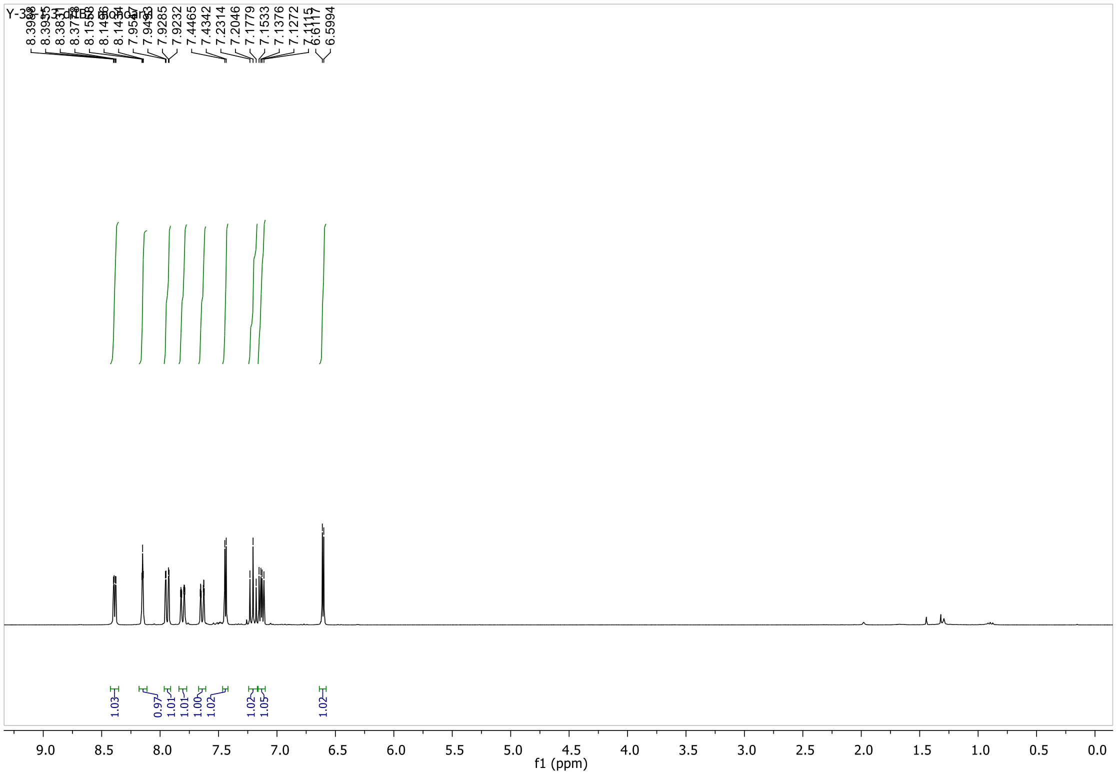

^{13}C NMR (75 MHz, $CDCl_3$)

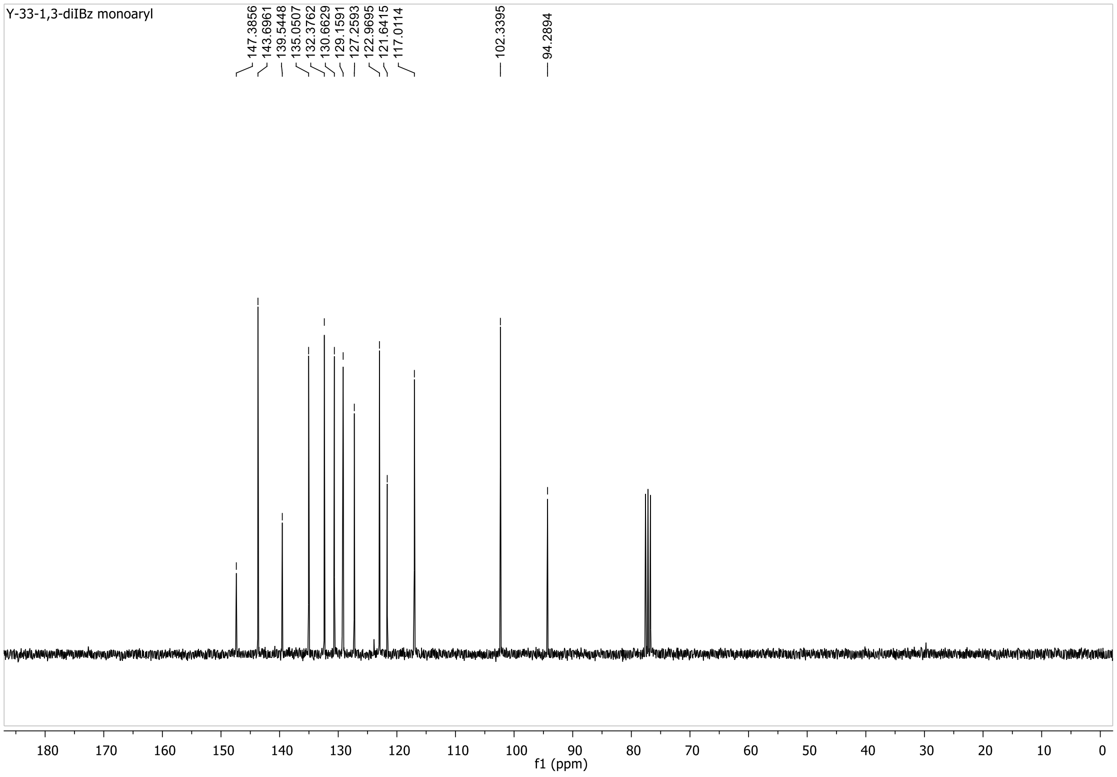

2-Iodo-1-phenyl-1*H*-pyrrolo[2,3-*b*]pyridine (3a)

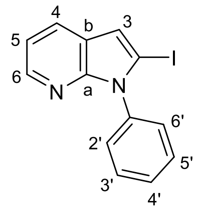

^{1}H NMR (300 MHz, $CDCl_3$)

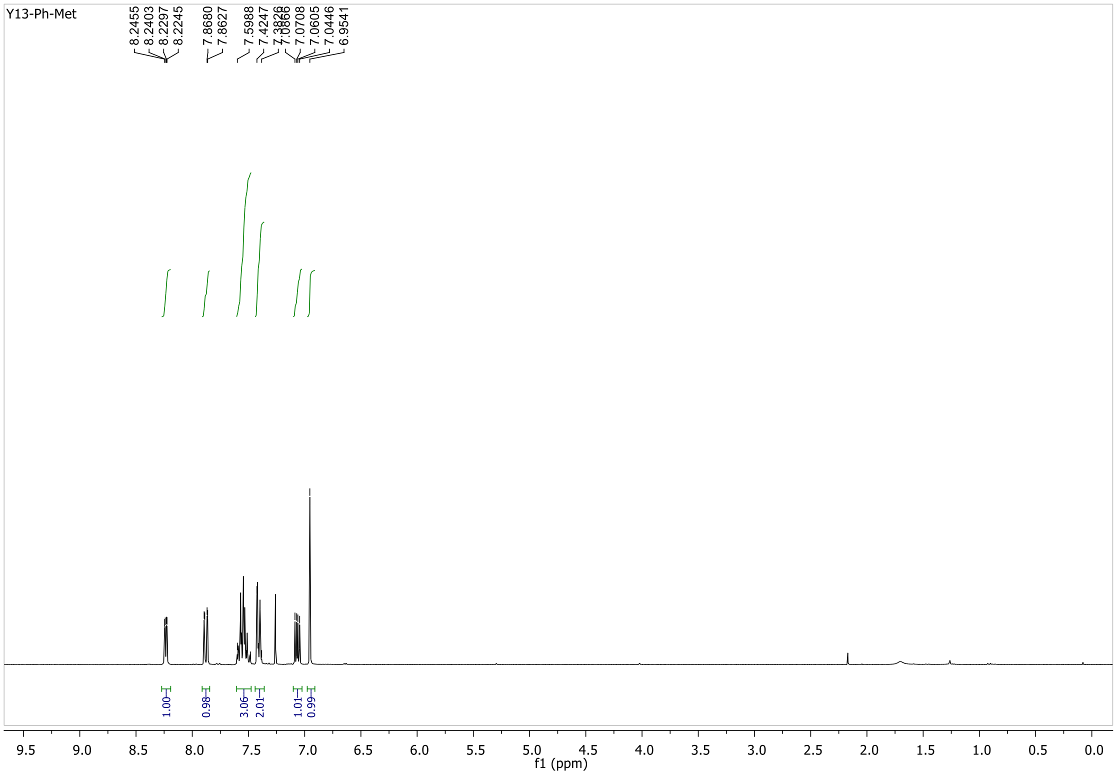

^{13}C NMR (75 MHz, $CDCl_3$)

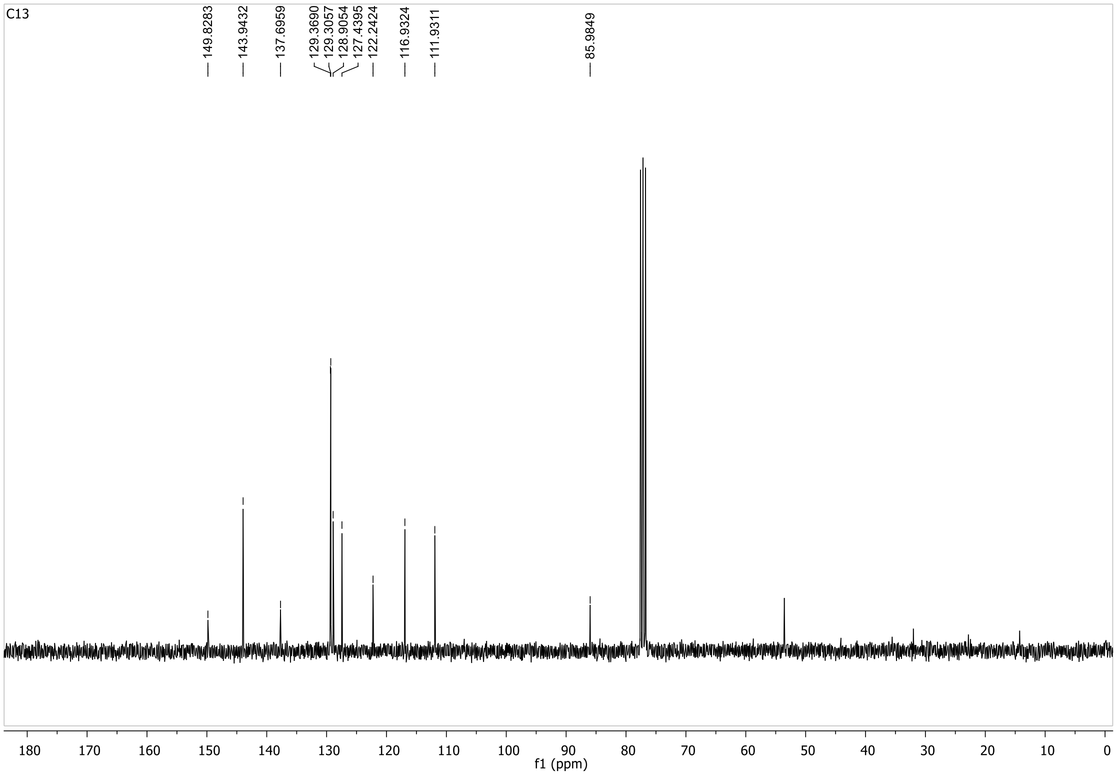

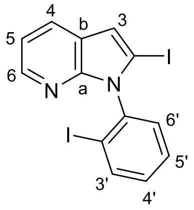

2-Iodo-1-(2-iodophenyl)-1*H*-pyrrolo[2,3-*b*]pyridine (3a')

^{1}H NMR (300 MHz, $CDCl_3$)

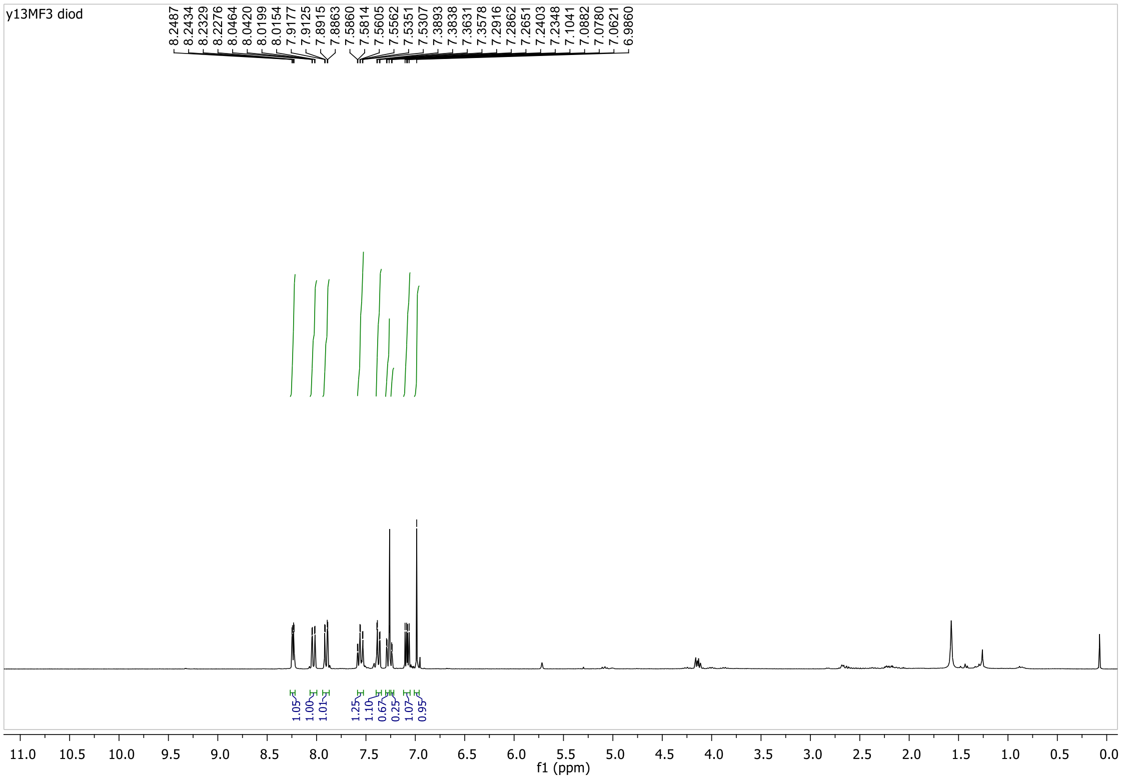

^{13}C NMR (75 MHz, $CDCl_3$)

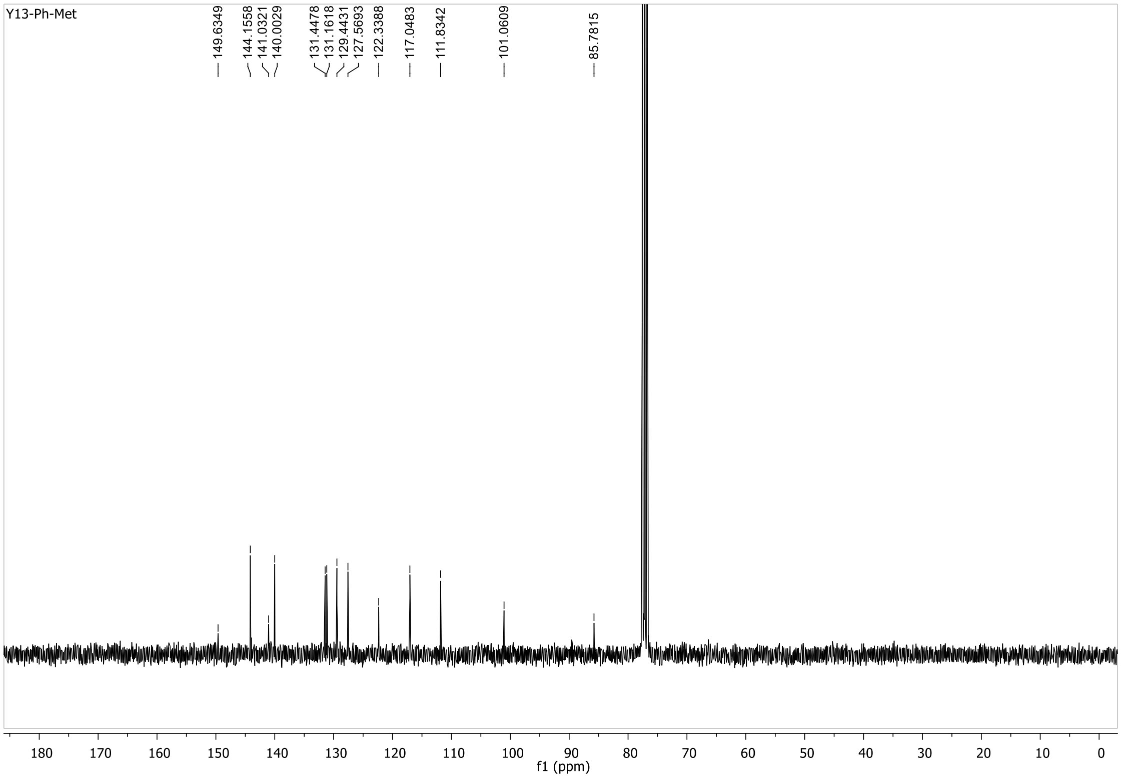

2-Iodo-1-(4-(trifluoromethyl)phenyl)-1*H*-pyrrolo[2,3-*b*]pyridine (3e)

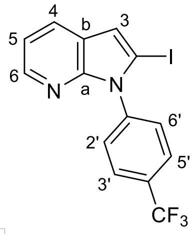

^1H NMR (300 MHz, $CDCl_3$)

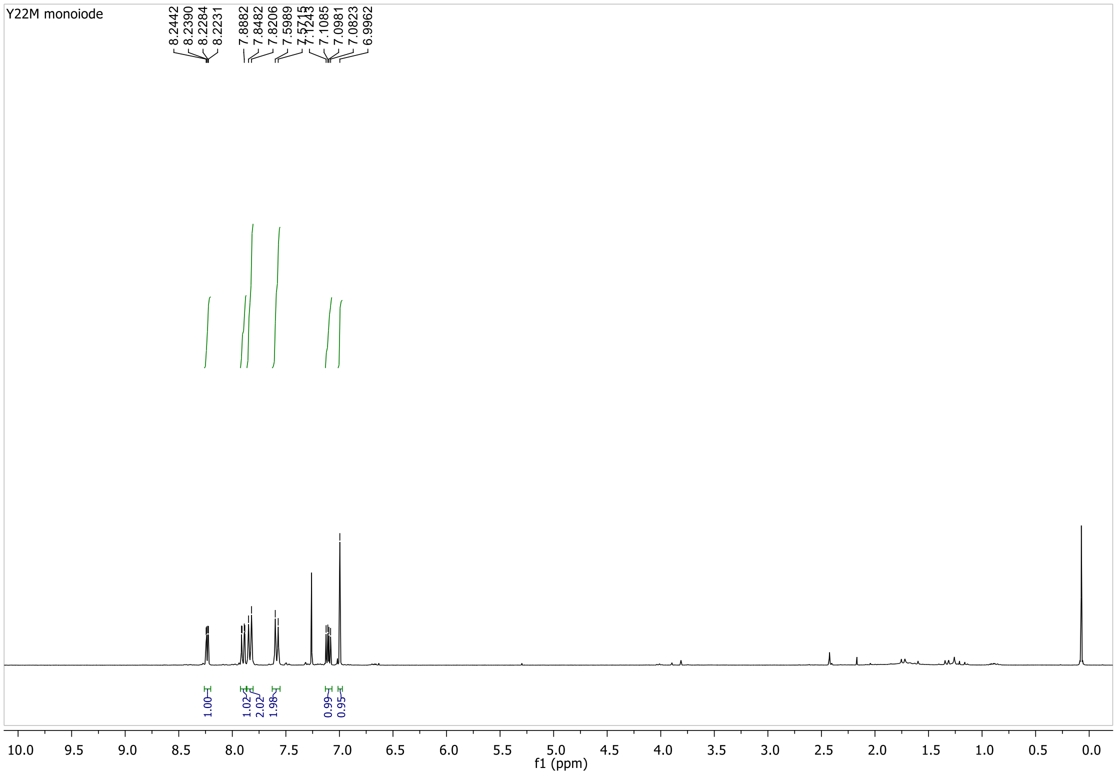

^{13}C NMR (75 MHz, $CDCl_3$)

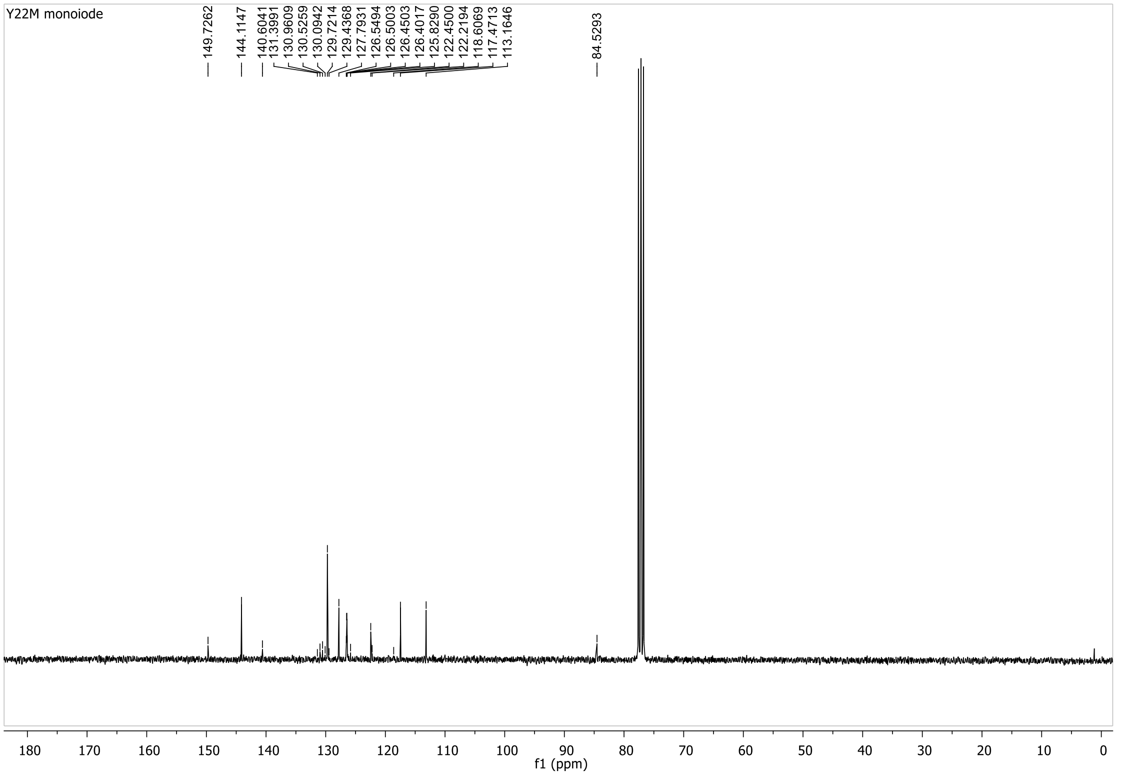

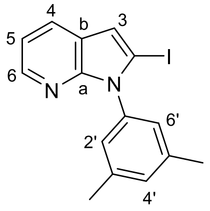

1-(3,5-Dimethylphenyl)-2-iodo-1*H*-pyrrolo[2,3-*b*]pyridine (3f)

^{1}H NMR (300 MHz, $CDCl_3$)

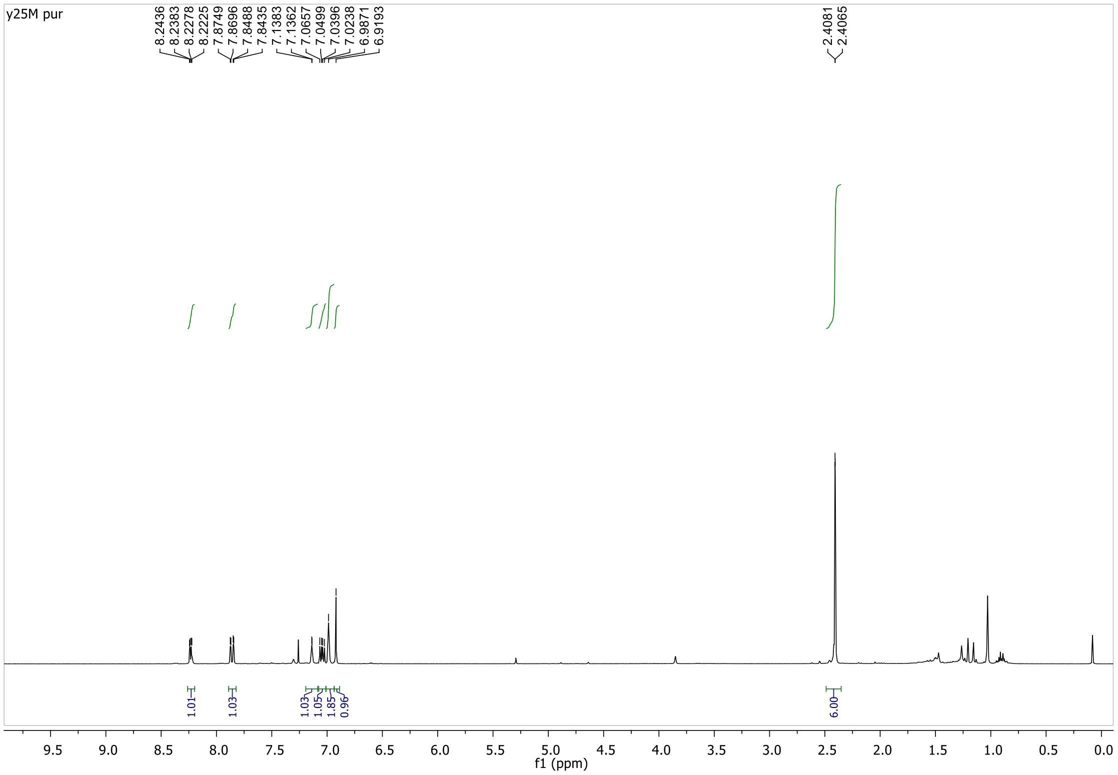

^{13}C NMR (75 MHz, $CDCl_3$)

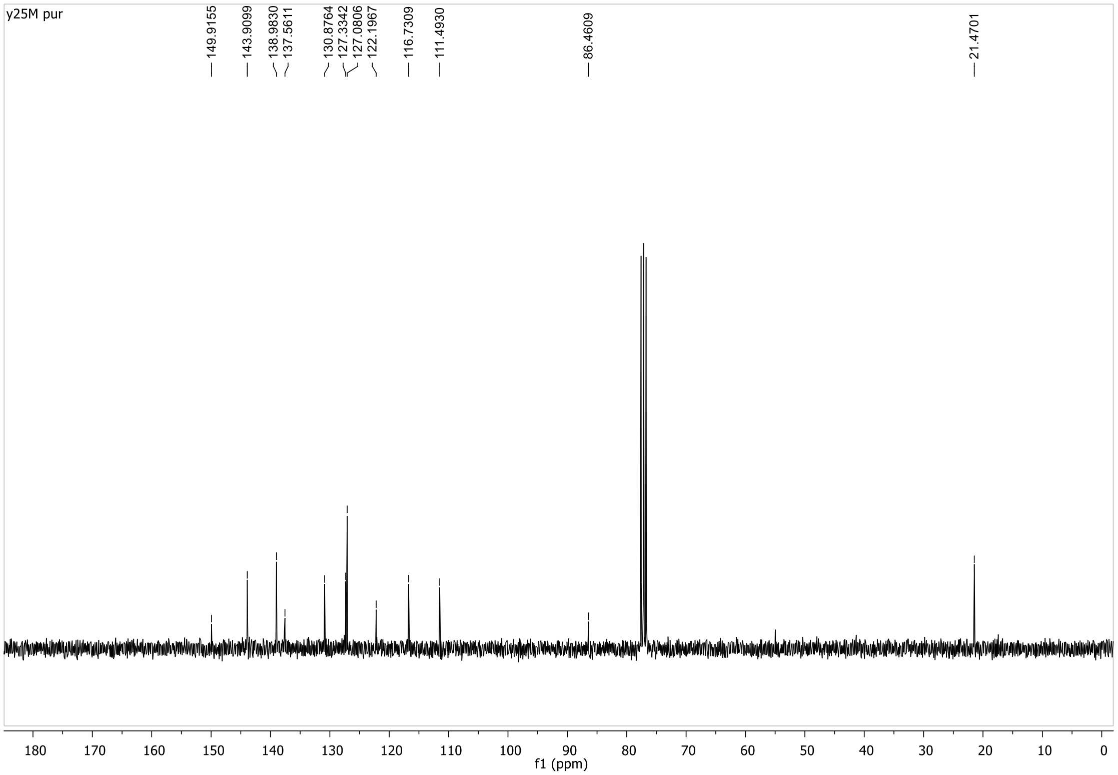

1-(5-Iodo-2-thienyl)-1*H*-pyrrolo[2,3-*b*]pyridine (3g)

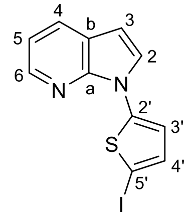

^{1}H NMR (300 MHz, $CDCl_3$)

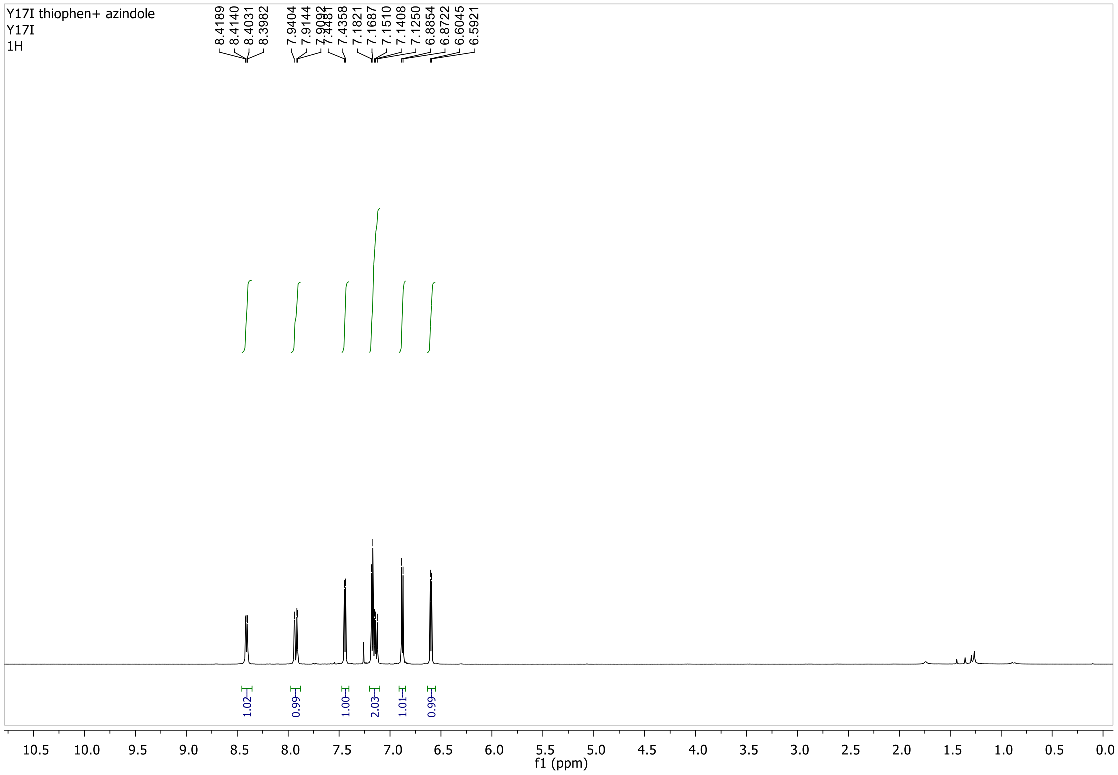

^{13}C NMR (75 MHz, $CDCl_3$)

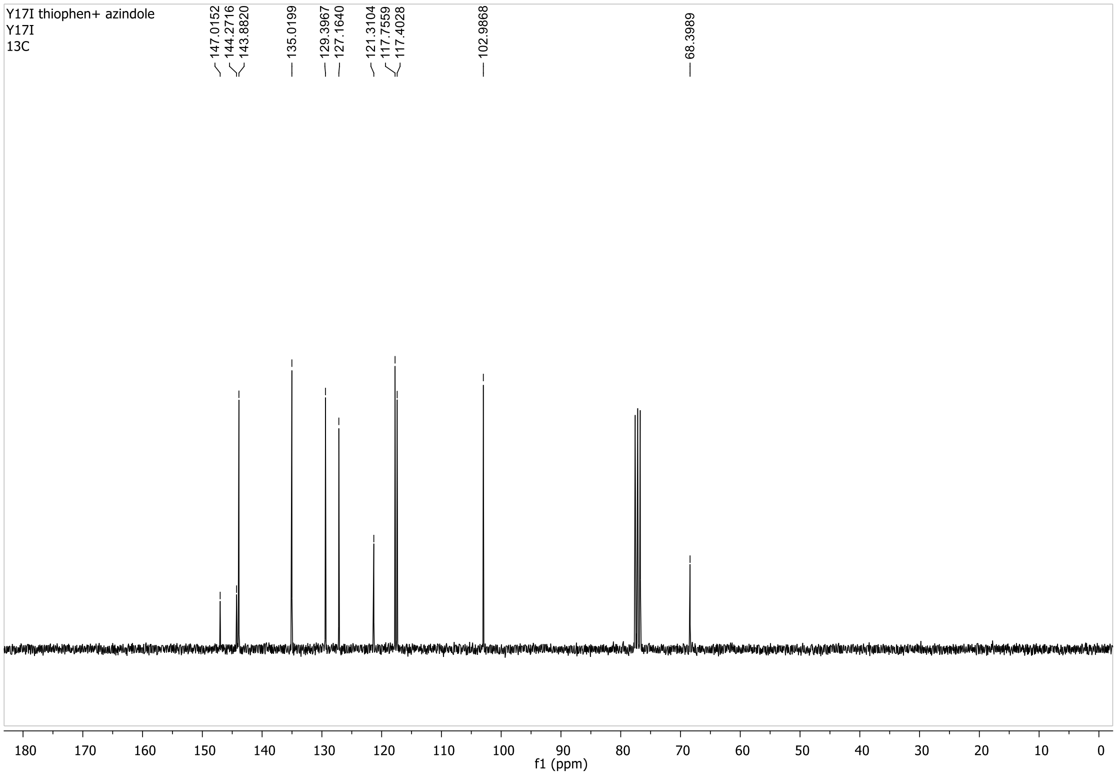

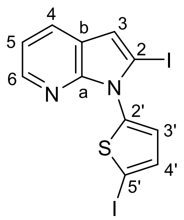

2-Iodo-1-(5-iodo-2-thienyl)-1*H*-pyrrolo[2,3-*b*]pyridine (3g')

^{1}H NMR (300 MHz, $CDCl_3$)

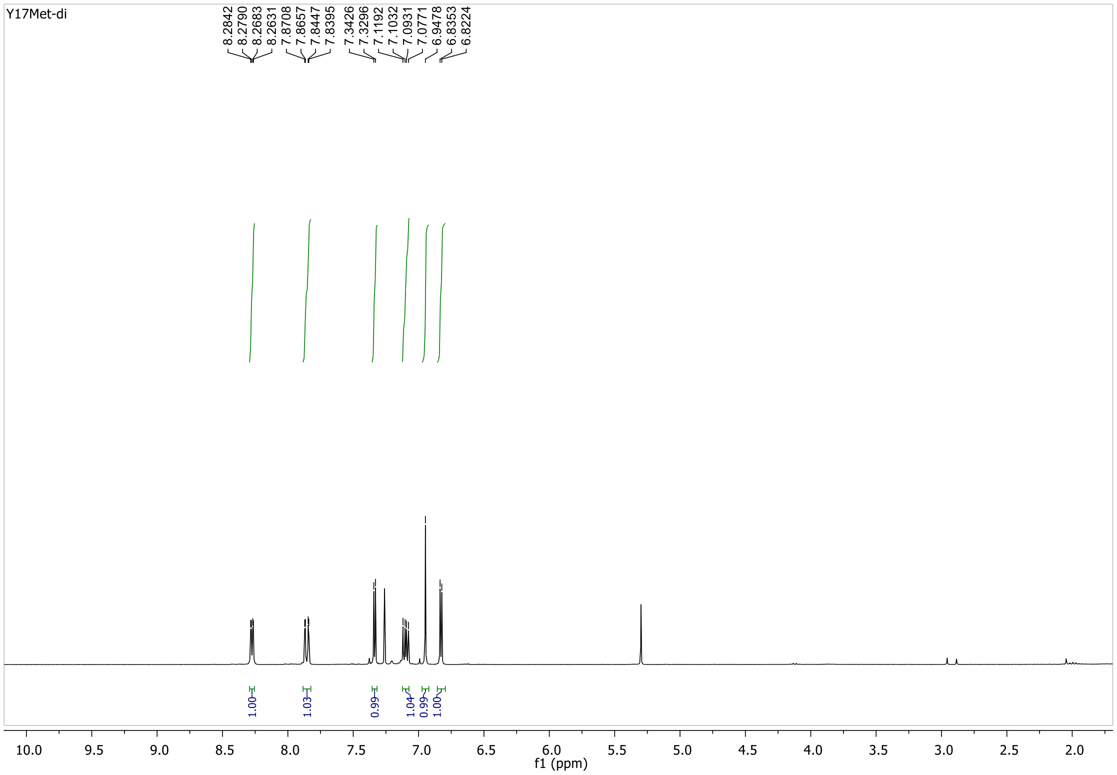

^{13}C NMR (75 MHz, $CDCl_3$)

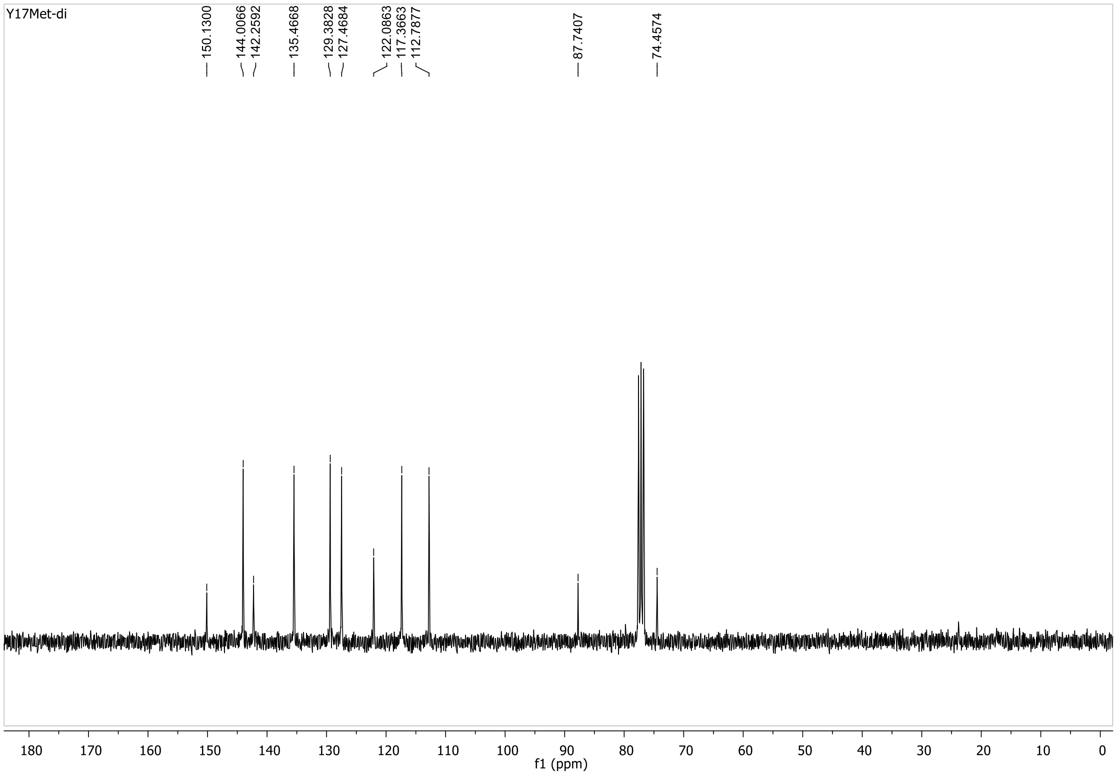

2-Iodo-1-(3-(7-azaindolyl)phenyl)-7-azaindole (3k)

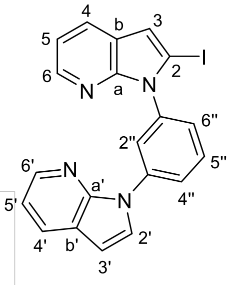

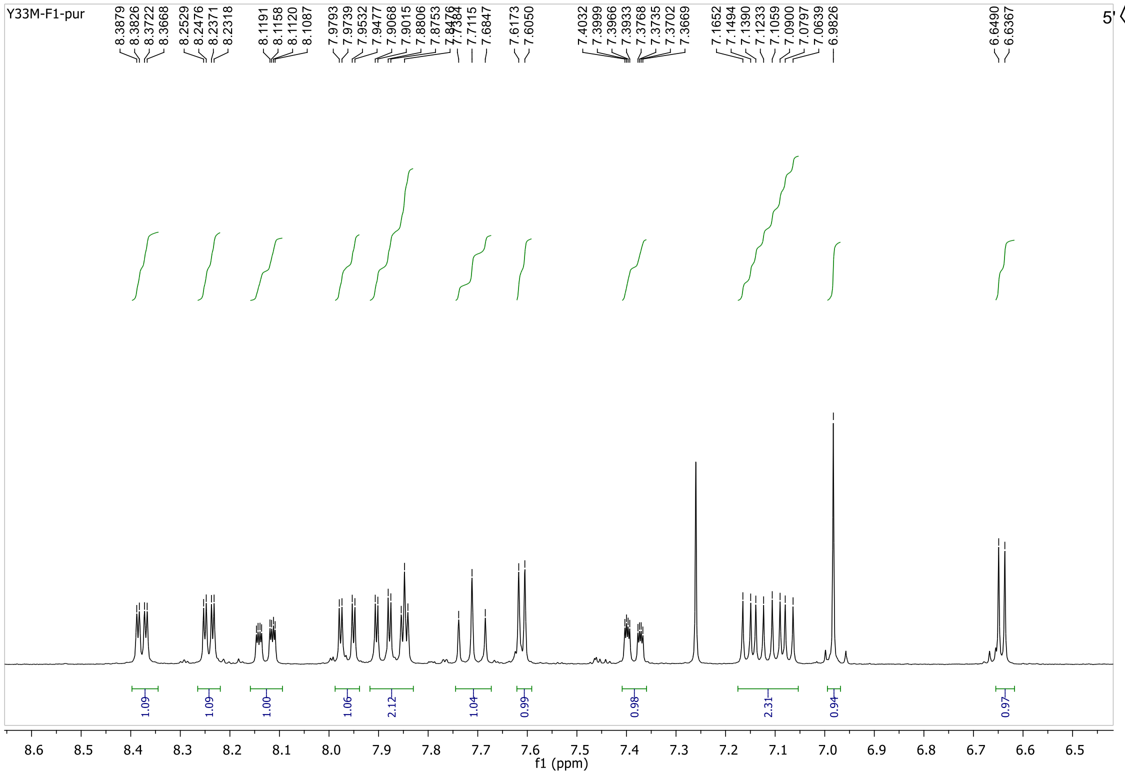

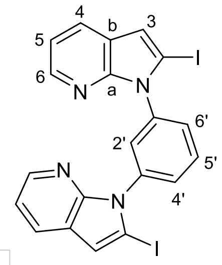

1,1'-(1,3-Phenylene)bis(2-iodo-1*H*-pyrrolo[2,3-*b*]pyridine) (3k')

^{1}H NMR (300 MHz, $CDCl_3$)

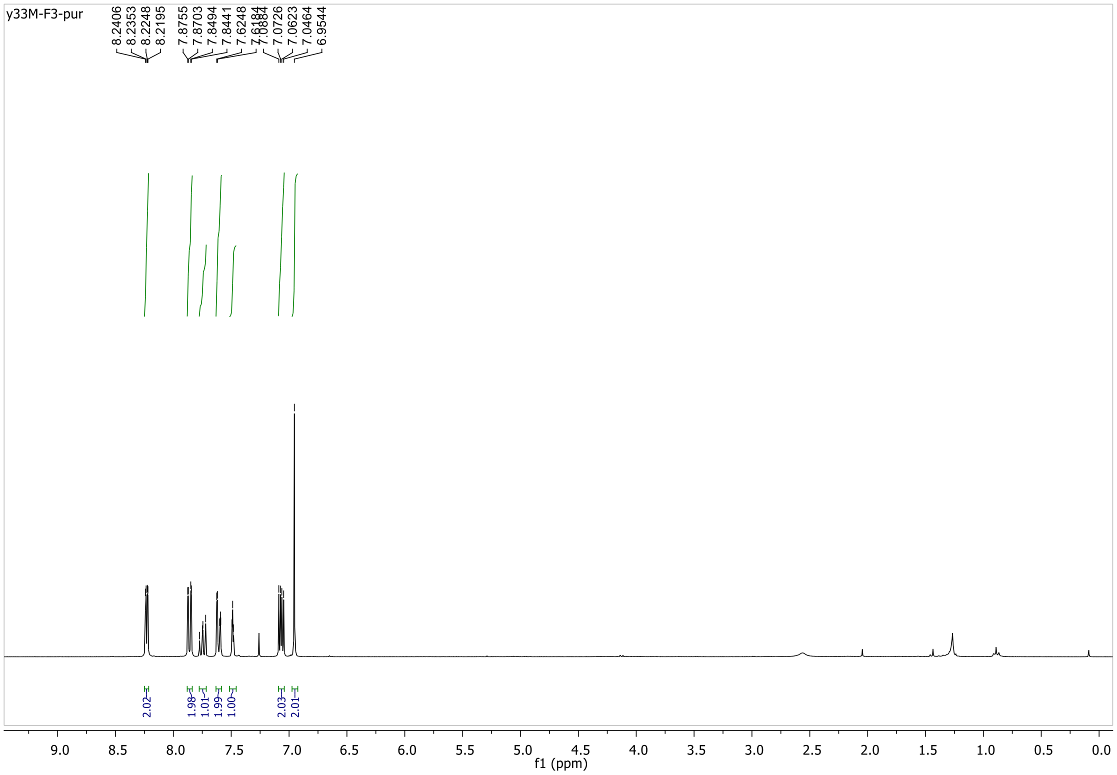

^{13}C NMR (75 MHz, $CDCl_3$)

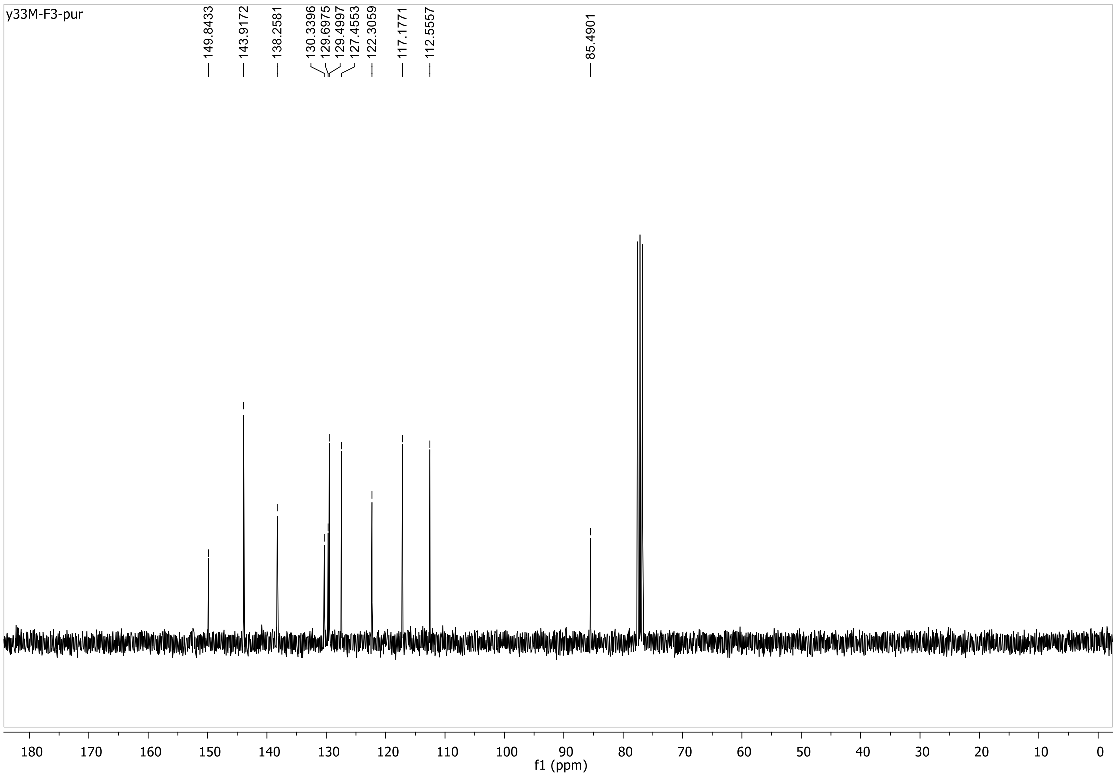

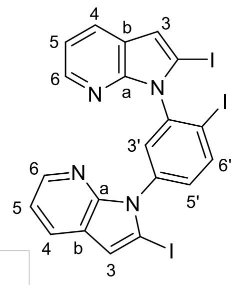

1,1'-(1-Iodo-2,4-phenylene)bis(2-iodo-1*H*-pyrrolo[2,3-*b*]pyridine (3k")

^{1}H NMR (300 MHz, $(CD_3)_2SO$)

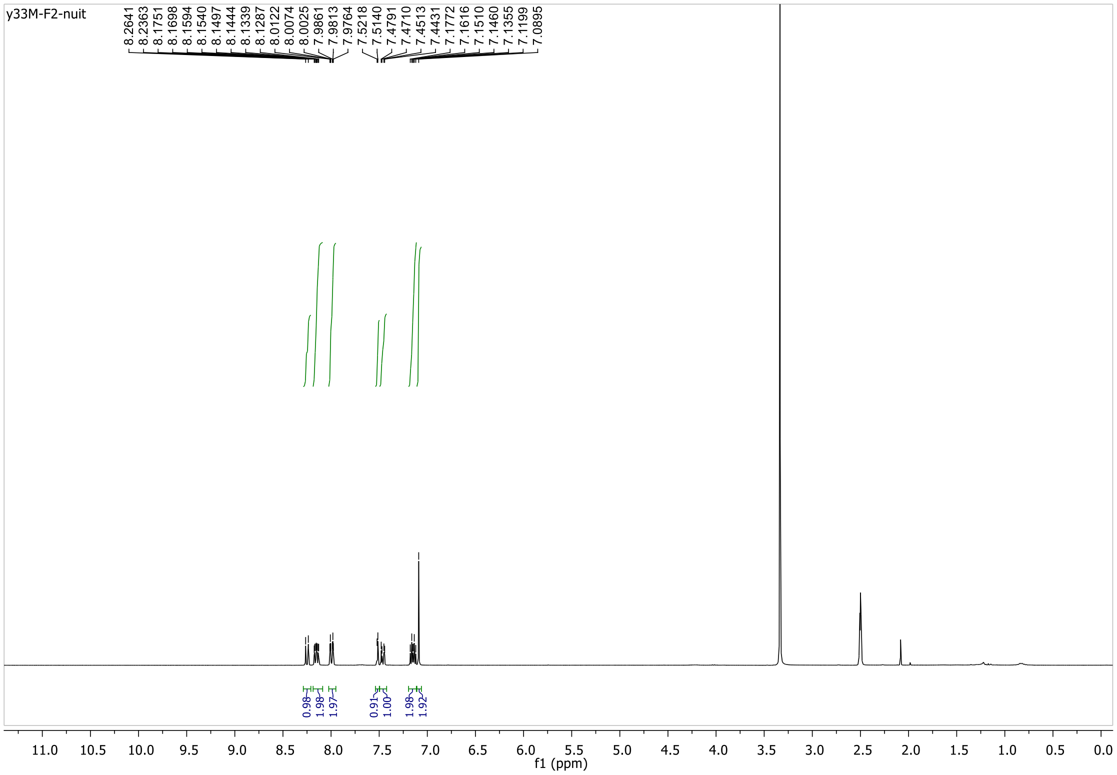

^{13}C NMR (75 MHz, $(CD_3)_2SO$)

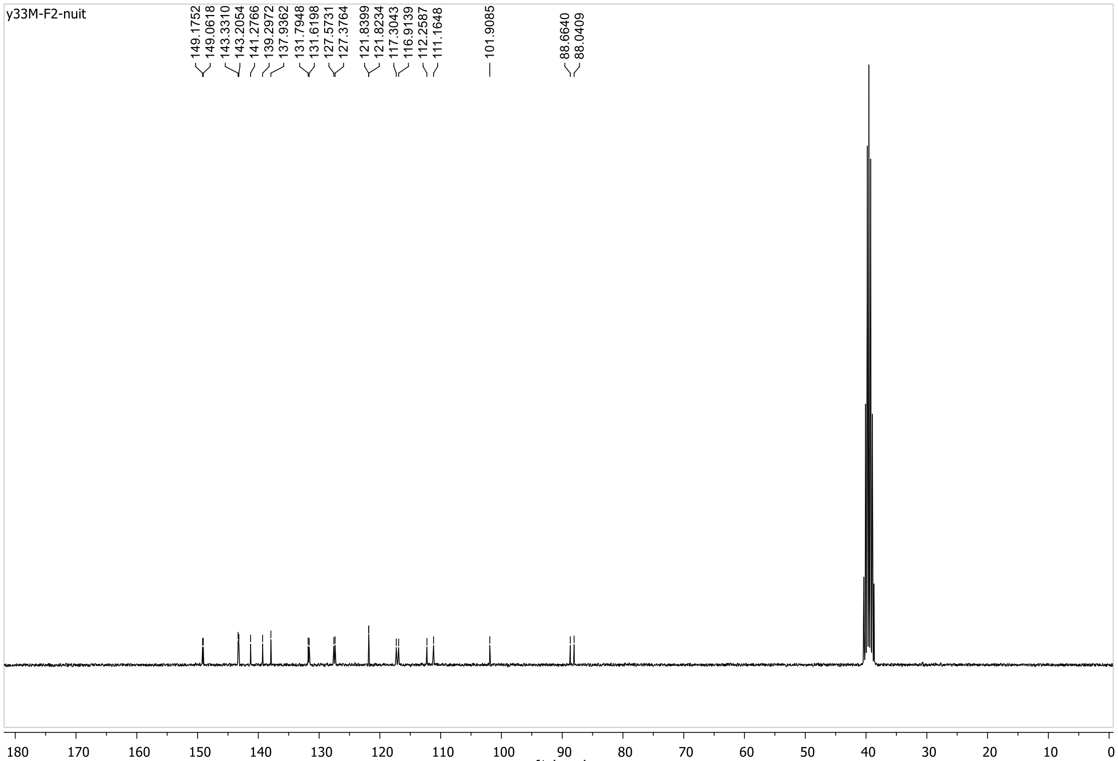

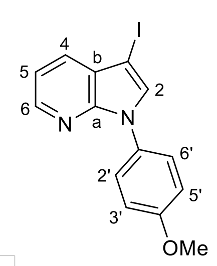

3-Iodo-1-(4-methoxyphenyl)-1*H*-pyrrolo[2,3-*b*]pyridine (4b)

^{1}H NMR (300 MHz, $CDCl_3$)

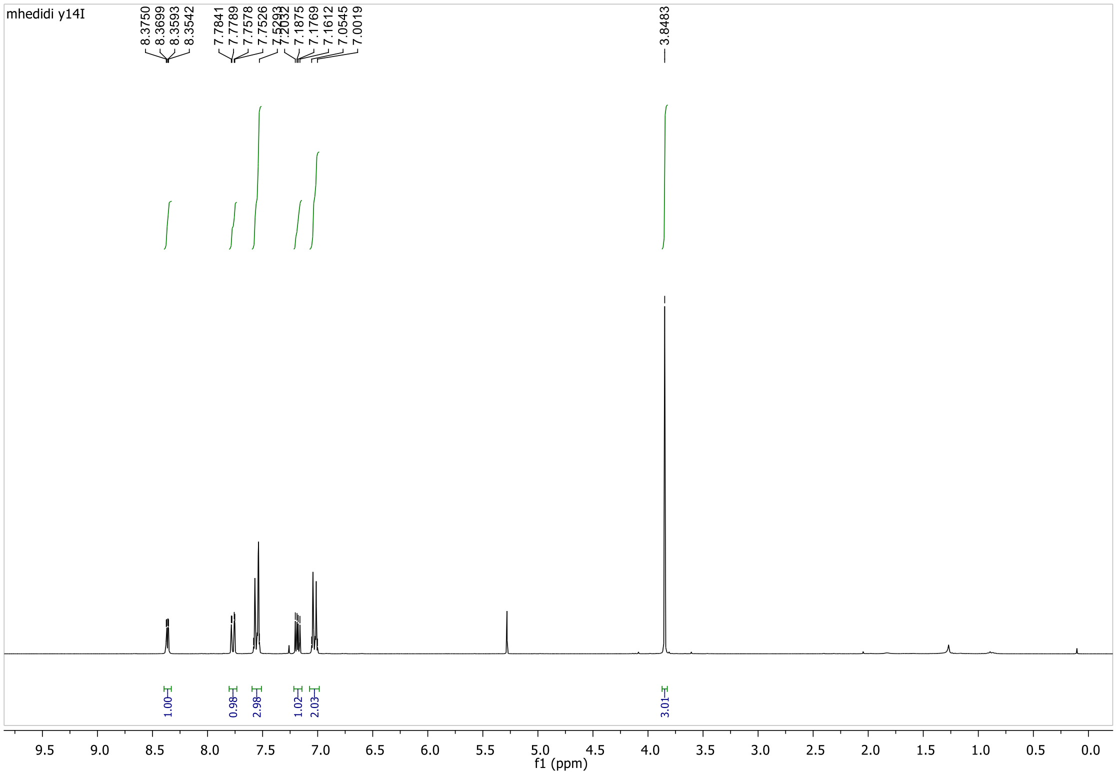

^{13}C NMR (75 MHz, $CDCl_3$)

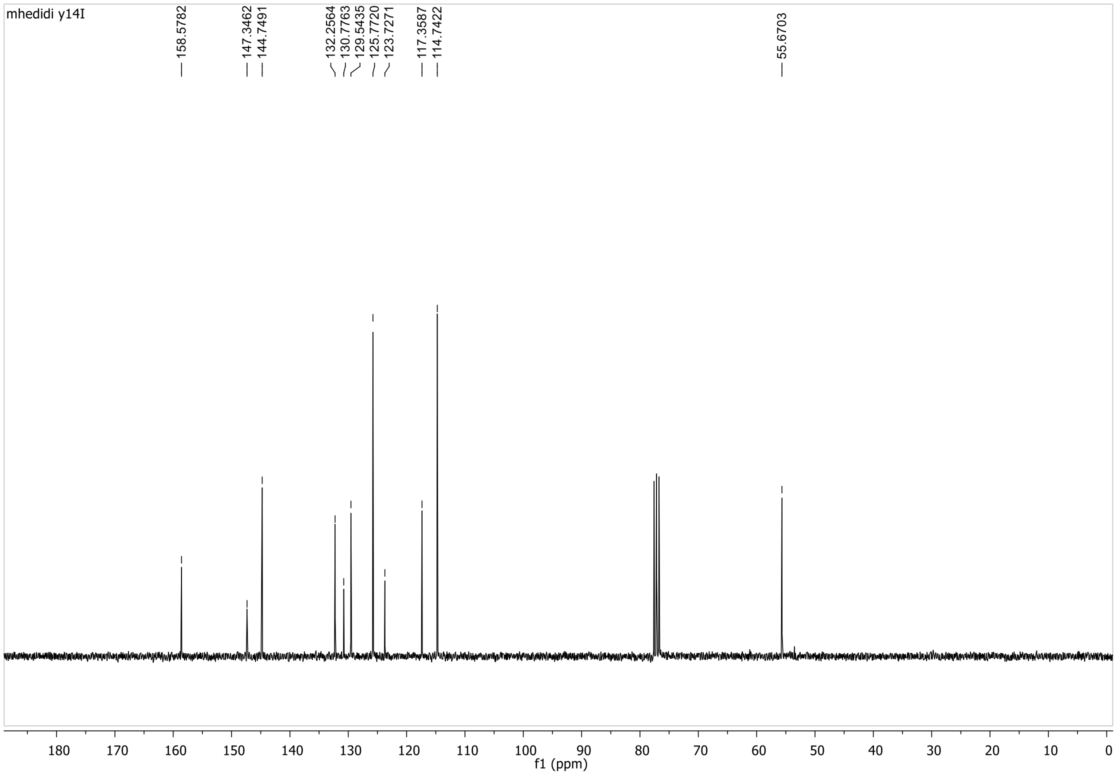

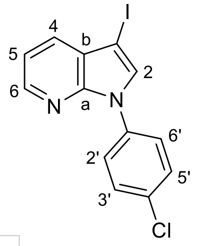

1-(4-Chlorophenyl)-3-iodo-1*H*-pyrrolo[2,3-*b*]pyridine (4c)

^{1}H NMR (300 MHz, $CDCl_3$)

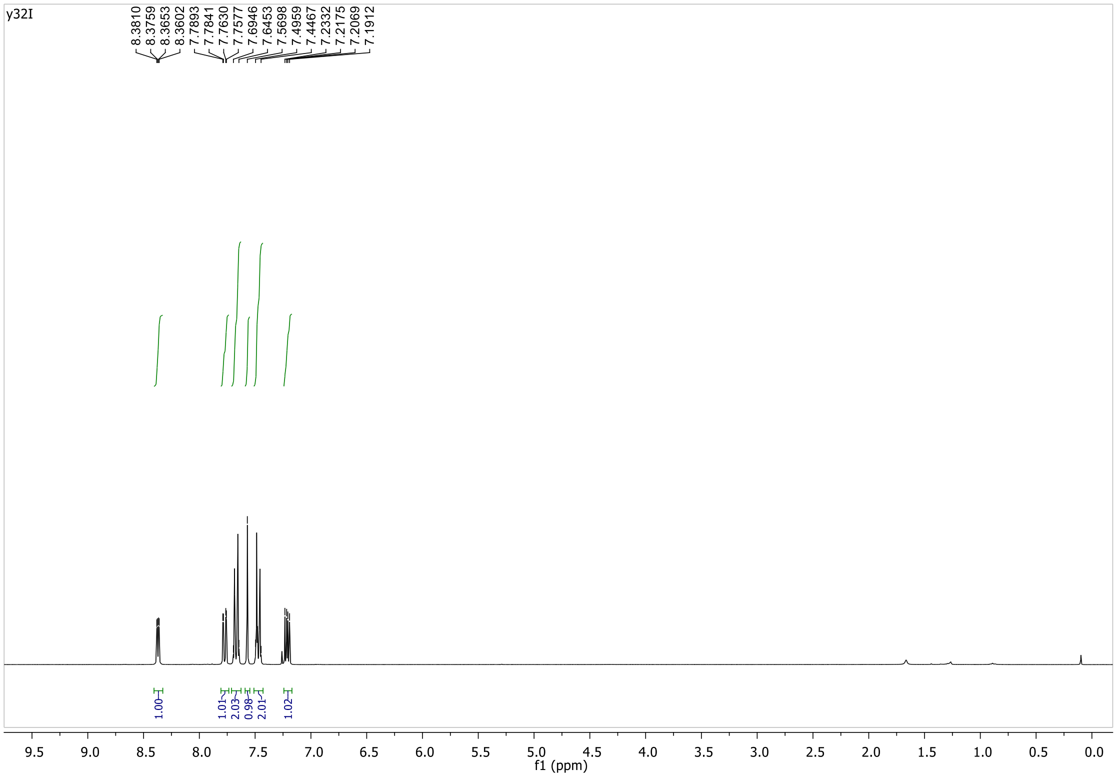

^{13}C NMR (75 MHz, $CDCl_3$)

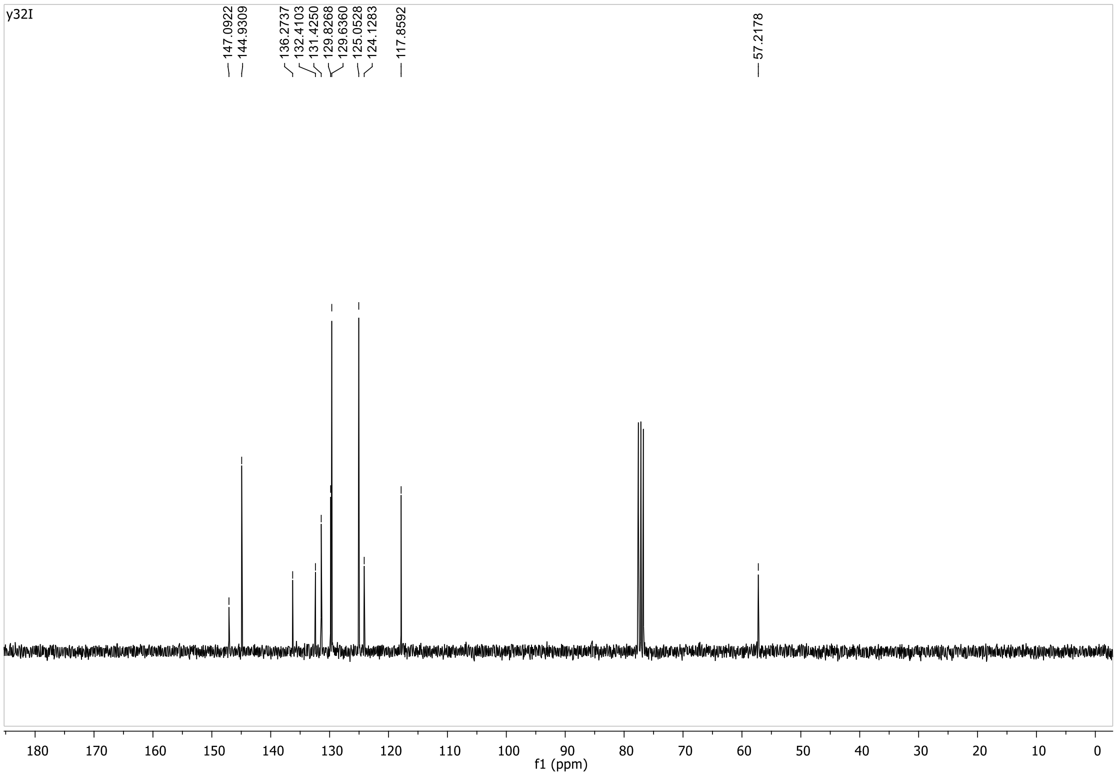

1-(4-Fluorophenyl)-3-iodo-1*H*-pyrrolo[2,3-*b*]pyridine (4d)

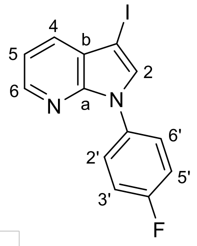

^{1}H NMR (300 MHz, $CDCl_3$)

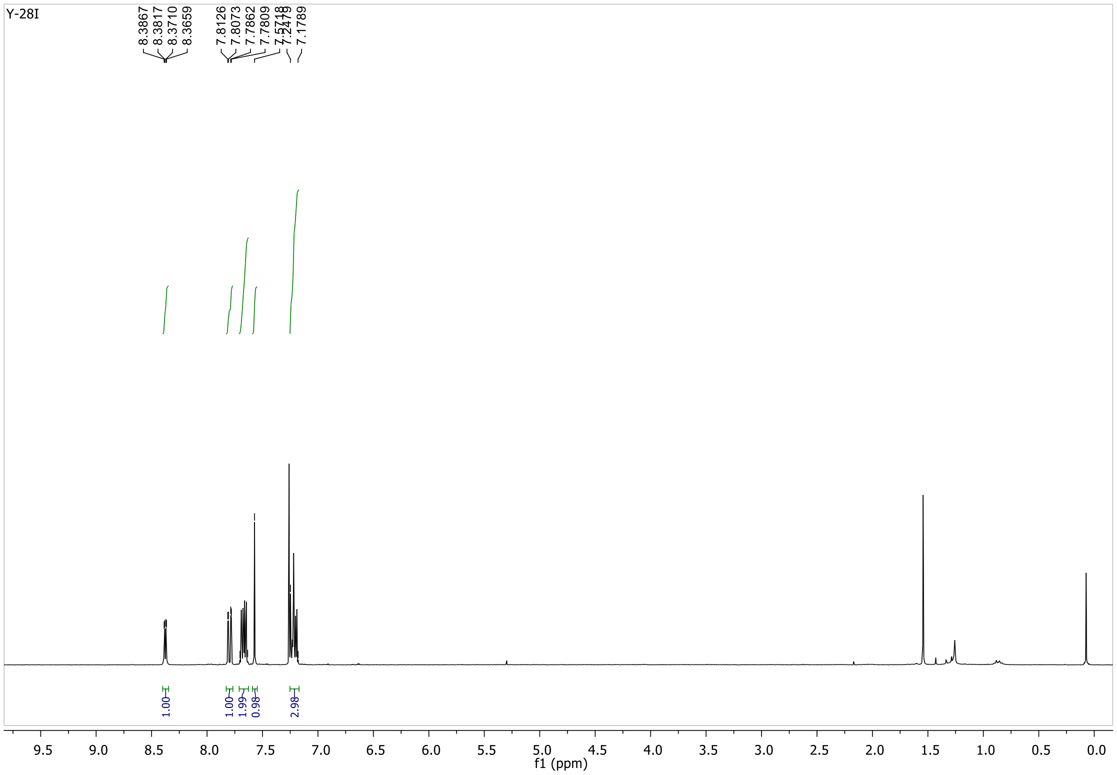

^{13}C NMR (75 MHz, $CDCl_3$)

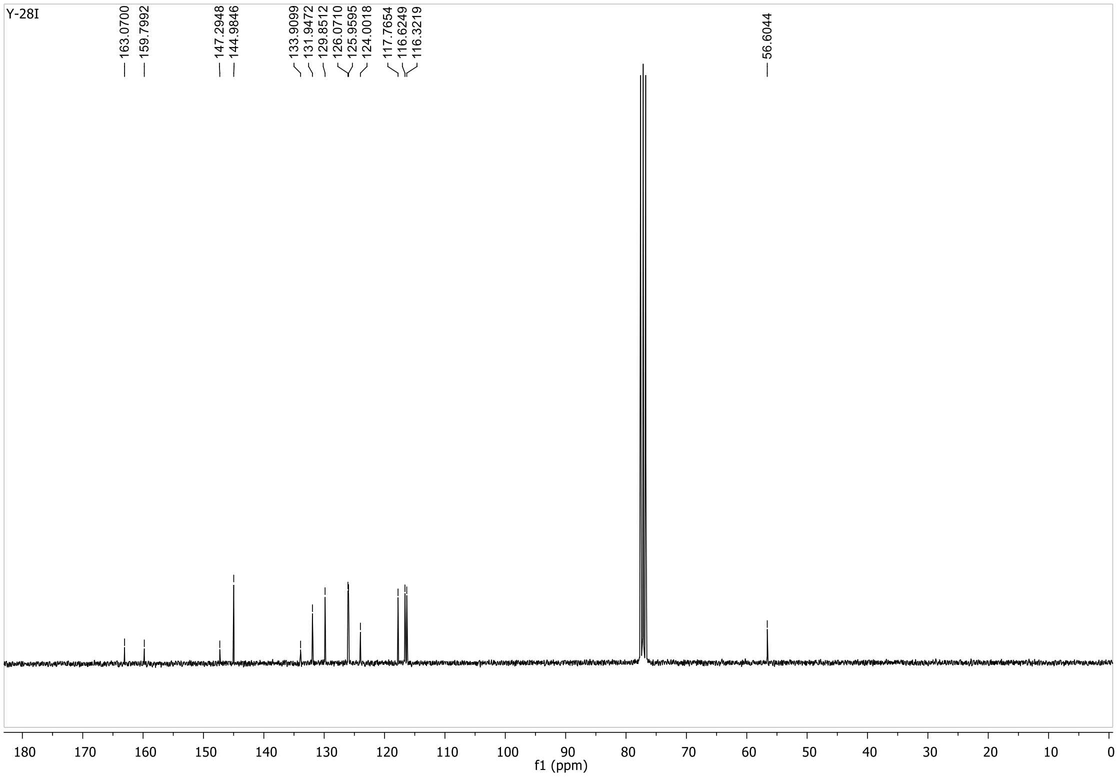

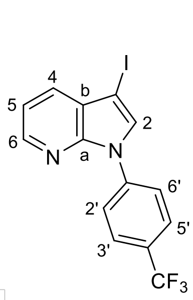

3-Iodo-1-(4-(trifluoromethyl)phenyl)-1*H*-pyrrolo[2,3-*b*]pyridine (4e)

^{1}H NMR (300 MHz, $CDCl_3$)

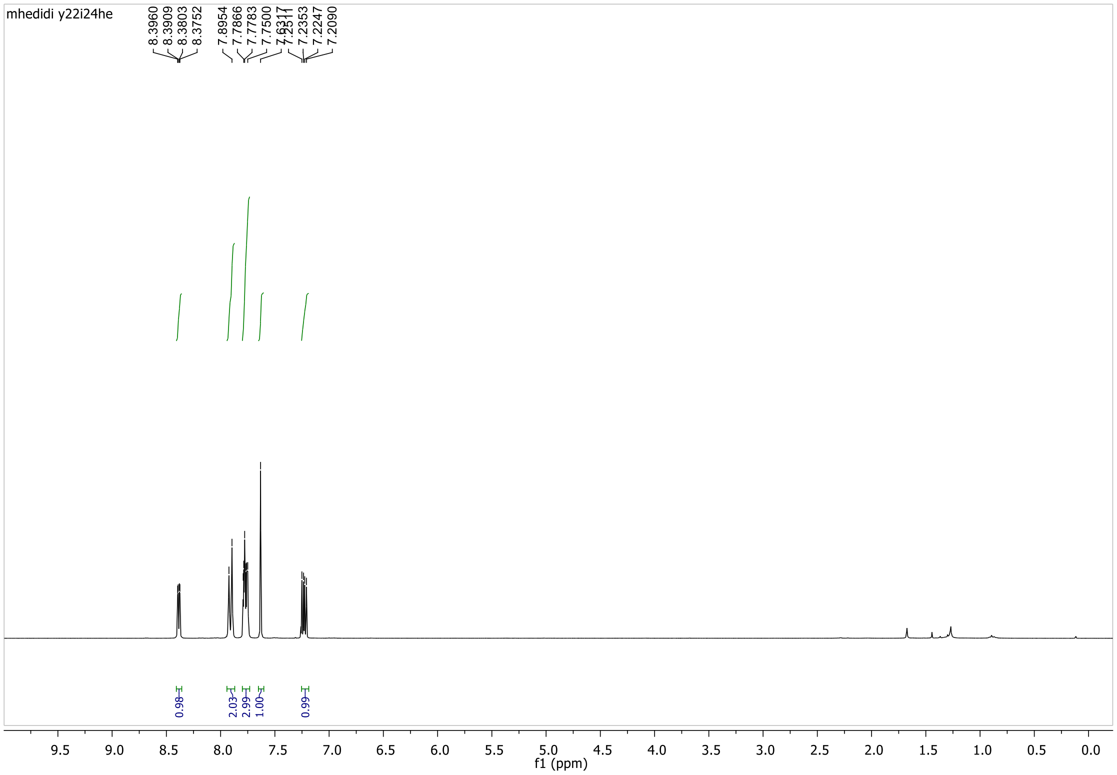

^{13}C NMR (75 MHz, $CDCl_3$)

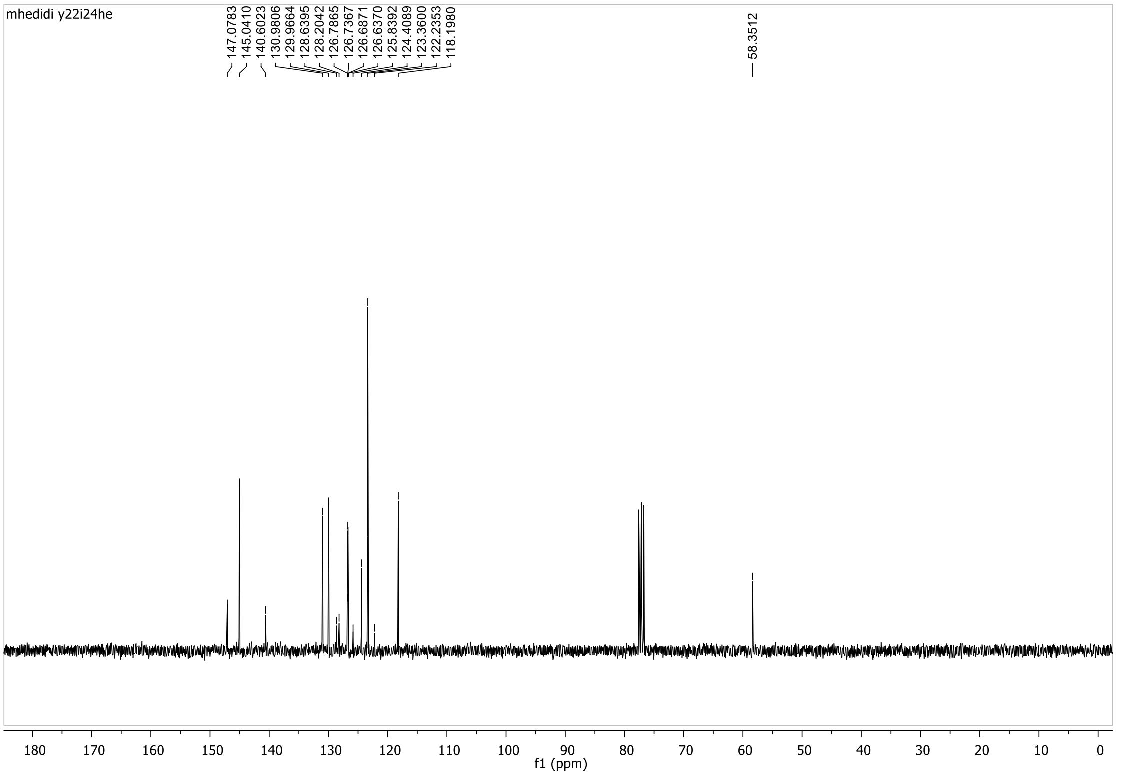

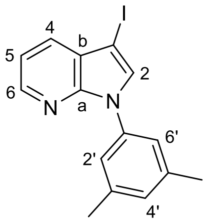

3-Iodo-1-(3,5-dimethylphenyl)-1*H*-pyrrolo[2,3-*b*]pyridine (4f)

^{1}H NMR (300 MHz, $CDCl_3$)

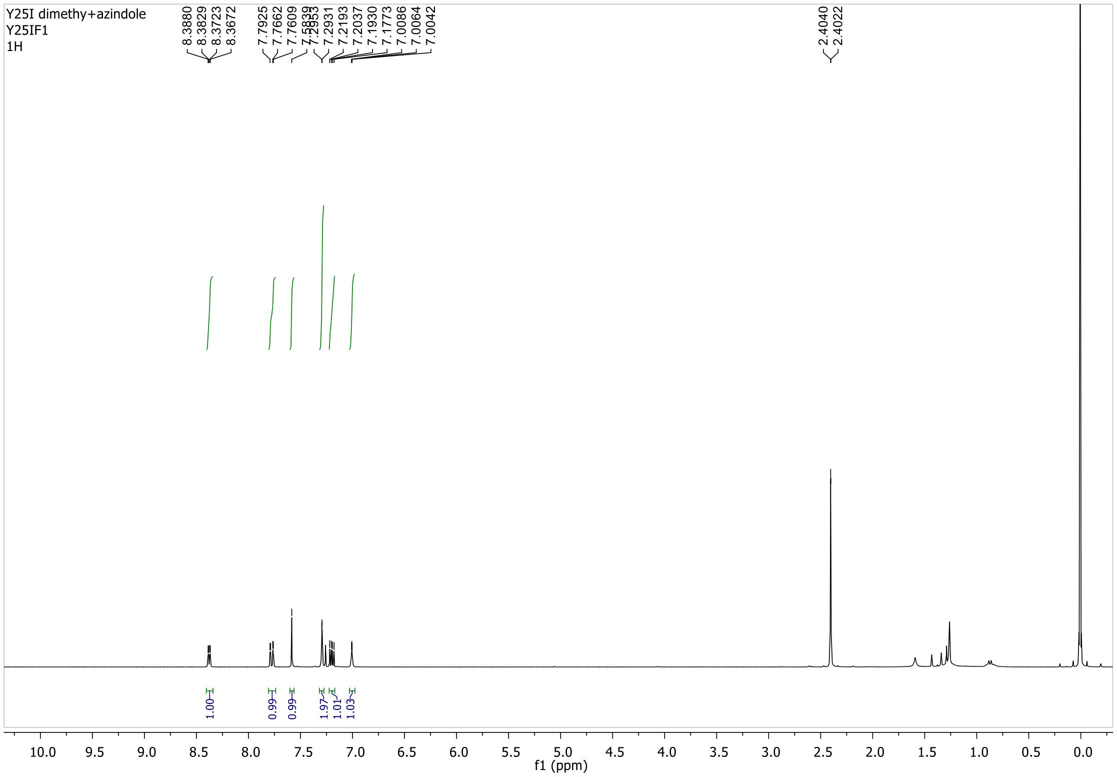

^{13}C NMR (75 MHz, $CDCl_3$)

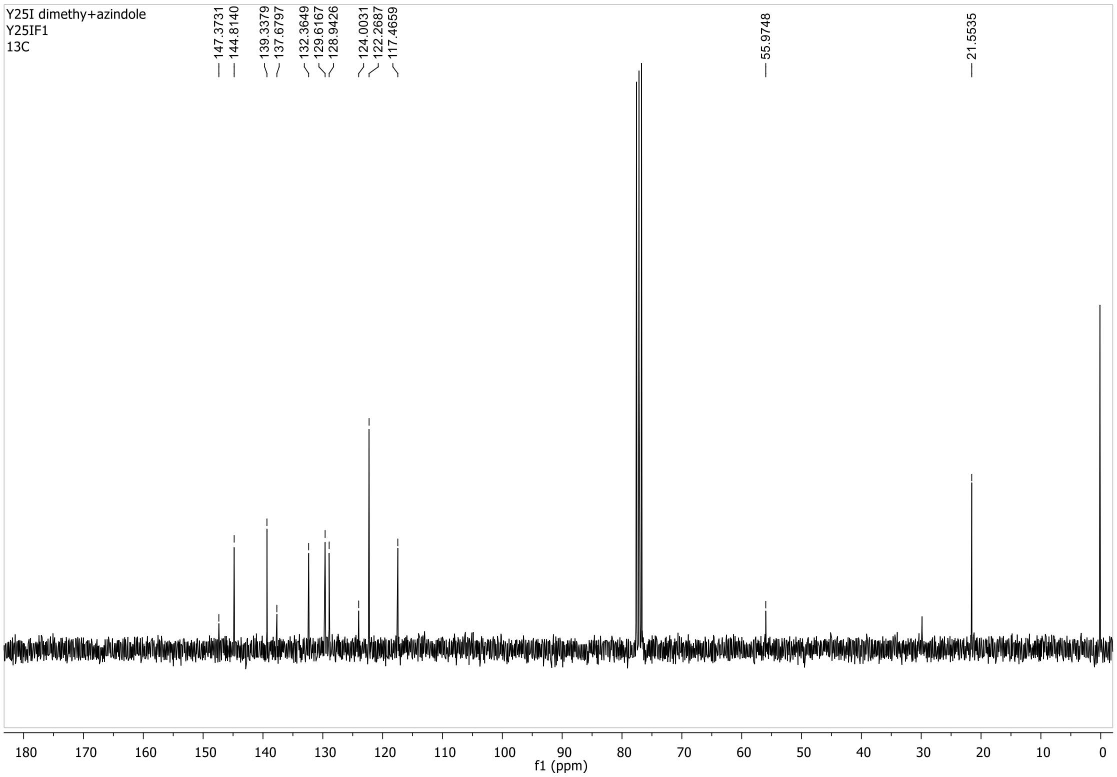

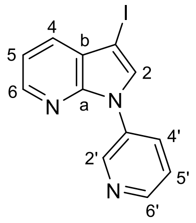

3-Iodo-1-(3-pyridyl)-1*H*-pyrrolo[2,3-*b*]pyridine (4h)

^{1}H NMR (300 MHz, $CDCl_3$)

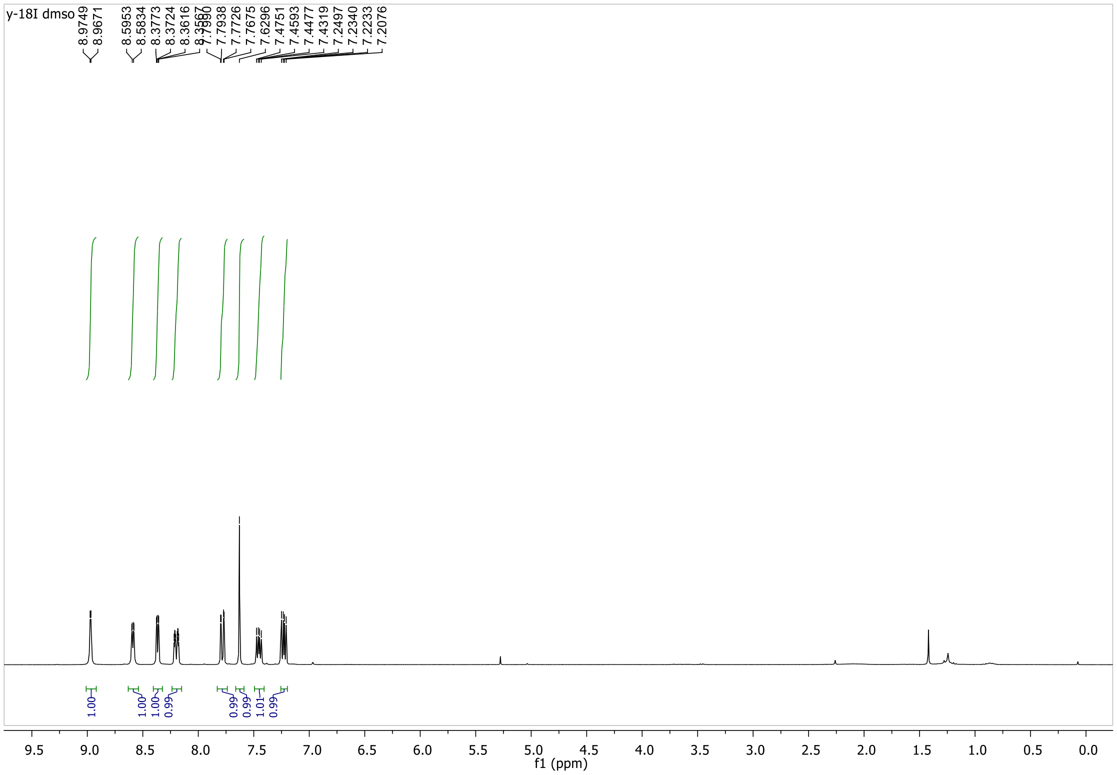

^{13}C NMR (75 MHz, $CDCl_3$)

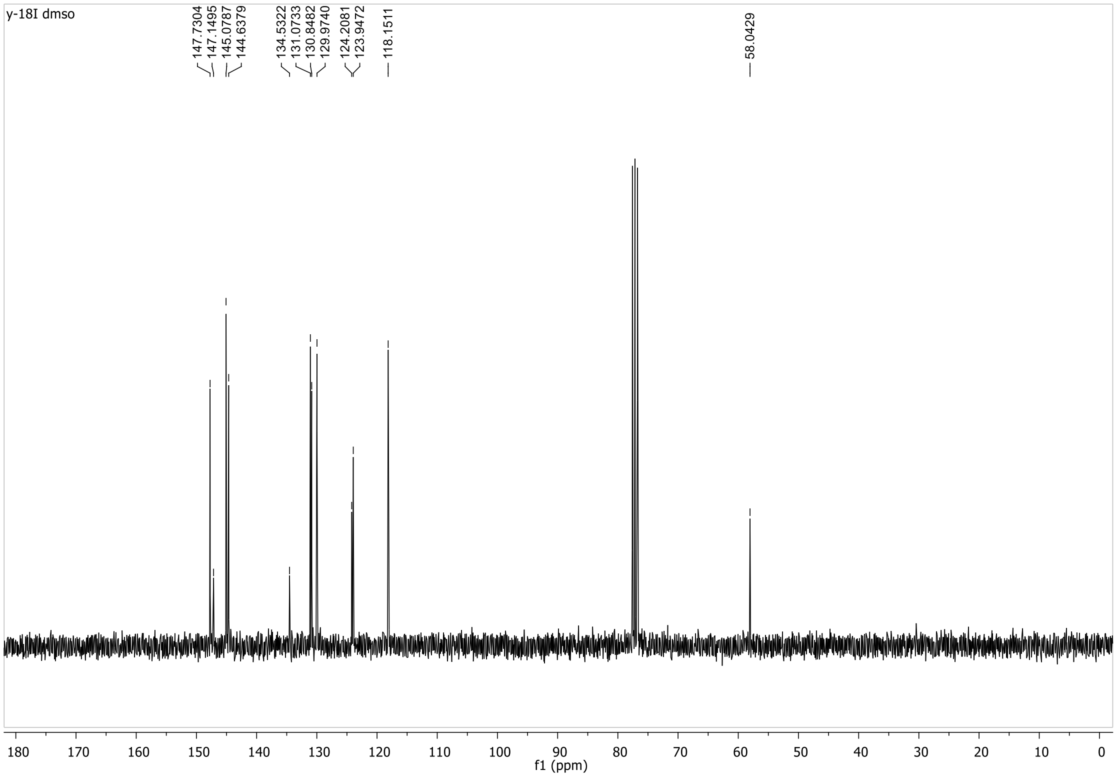

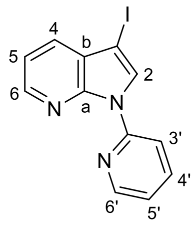

3-Iodo-1-(2-pyridyl)-1*H*-pyrrolo[2,3-*b*]pyridine (4i)

^{1}H NMR (300 MHz, $CDCl_3$)

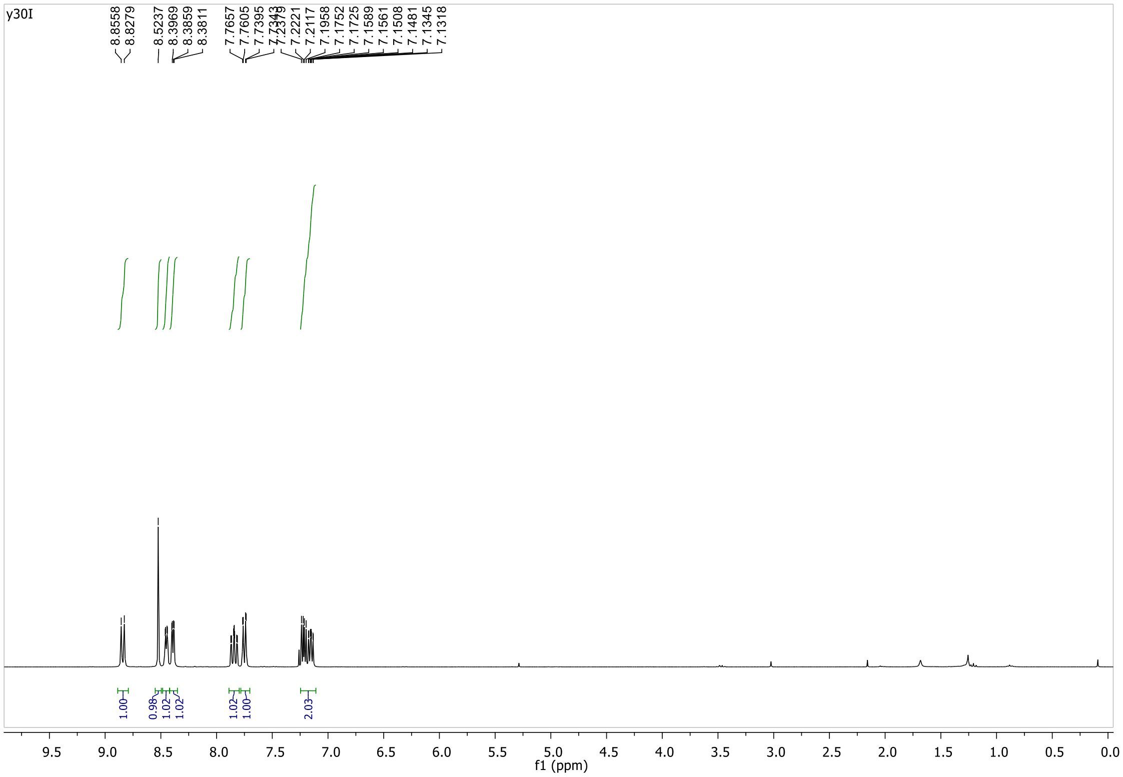

^{13}C NMR (75 MHz, $CDCl_3$)

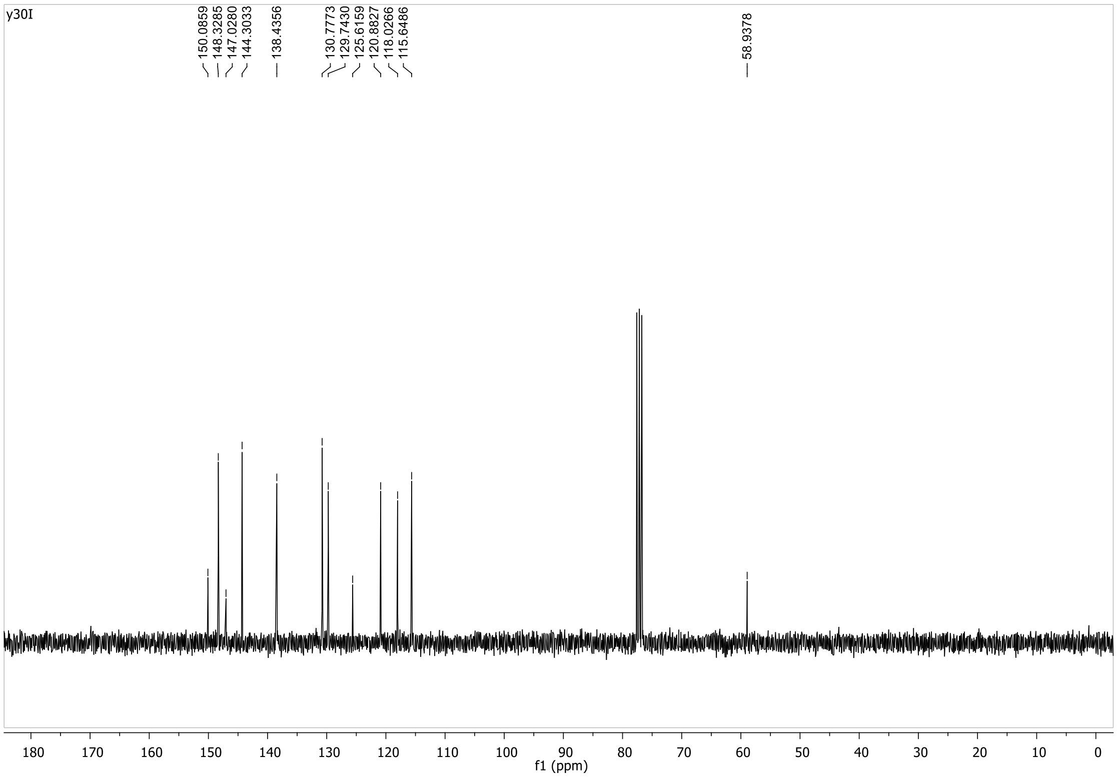

3-(1-Indolyl)-1-(4-(trifluoromethyl)phenyl)-1*H*-pyrrolo[2,3-*b*]pyridine (5e)

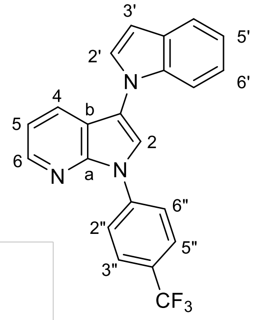

^{1}H NMR (300 MHz, $CDCl_3$)

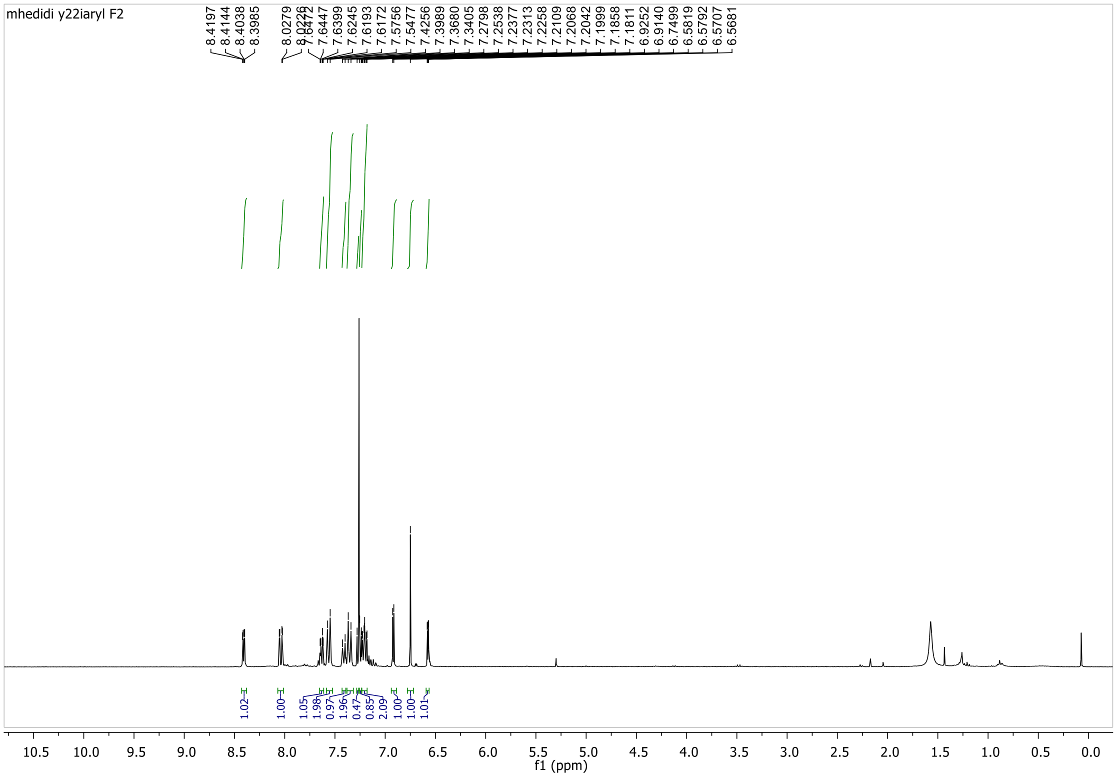

^{13}C NMR (75 MHz, $CDCl_3$)

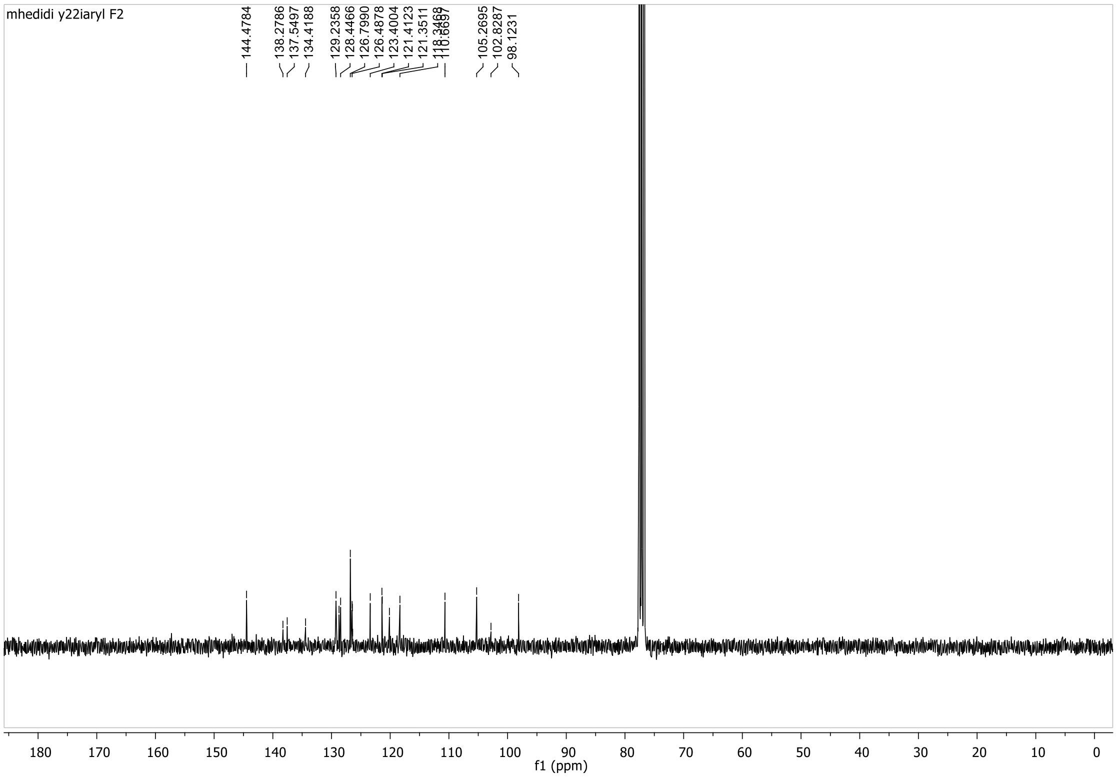

3-(1-Indolyl)-1-(2-pyridyl)-1*H*-pyrrolo[2,3-*b*]pyridine (5i)

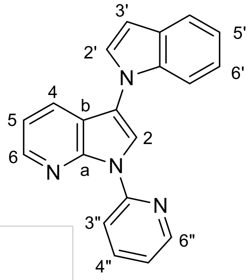

^{1}H NMR (300 MHz, $CDCl_3$)

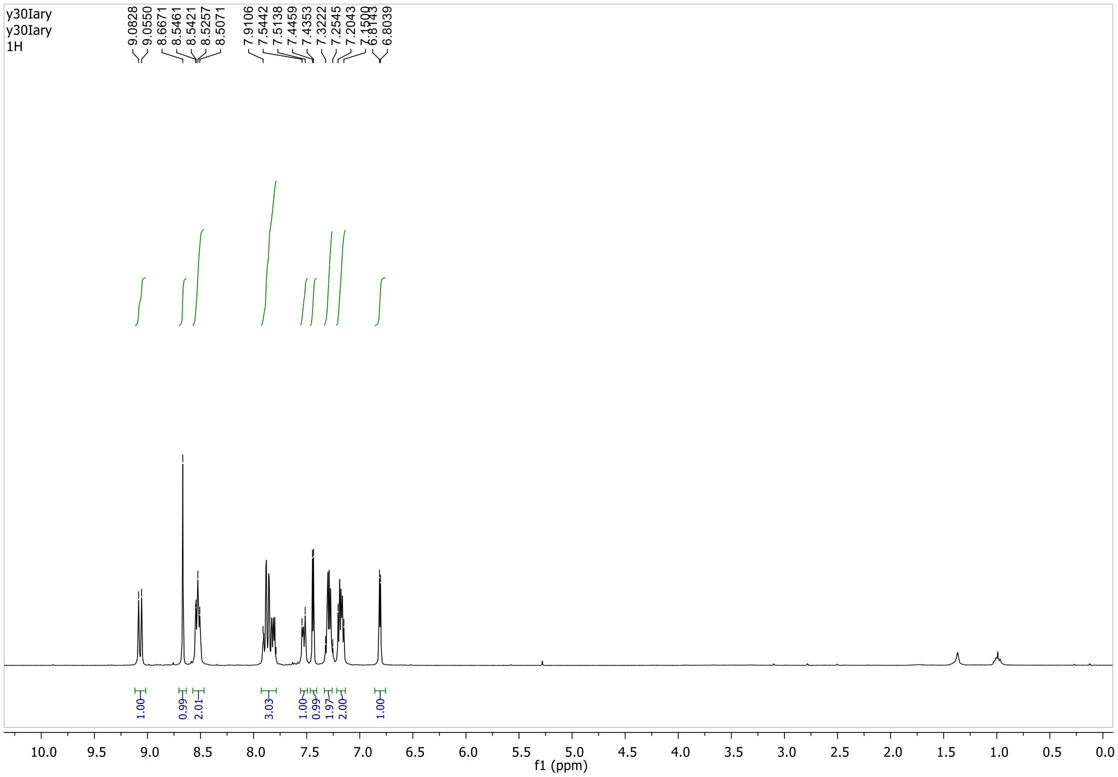

^{13}C NMR (75 MHz, $CDCl_3$)

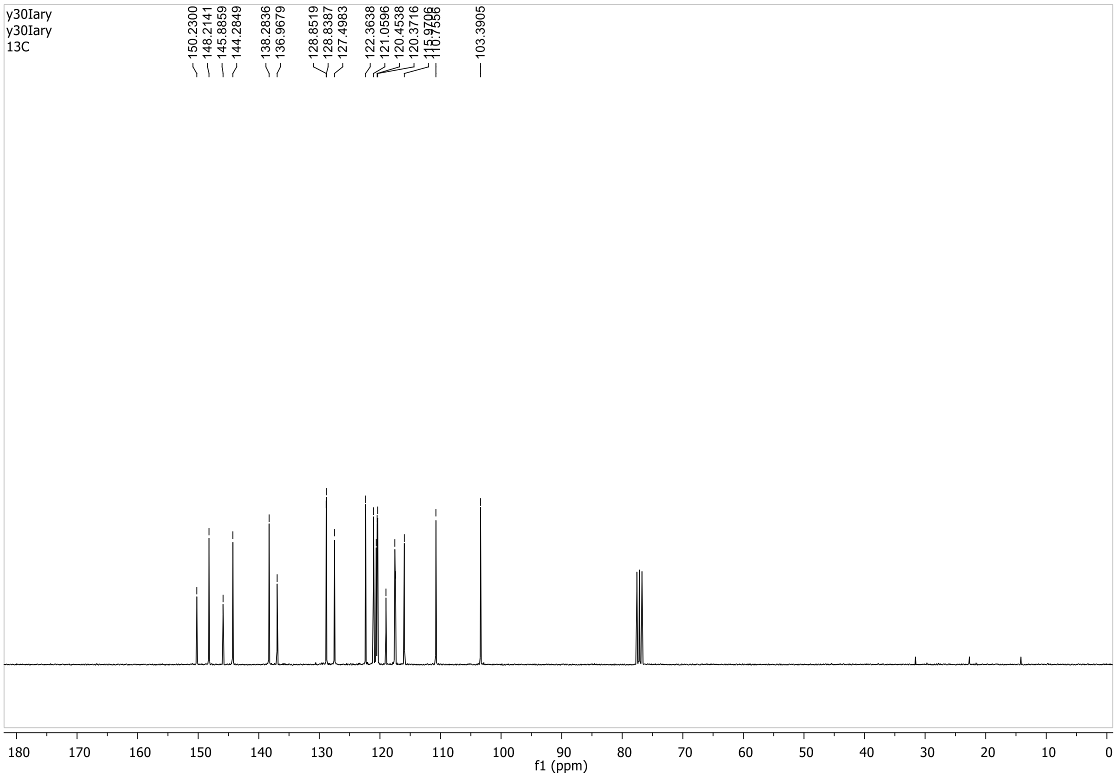

2-(1-Indolyl)-1-(5-(1-indolyl)-2-thienyl)-1*H*-pyrrolo[2,3-*b*]pyridine (6g')

^{1}H NMR (300 MHz, $CDCl_3$)

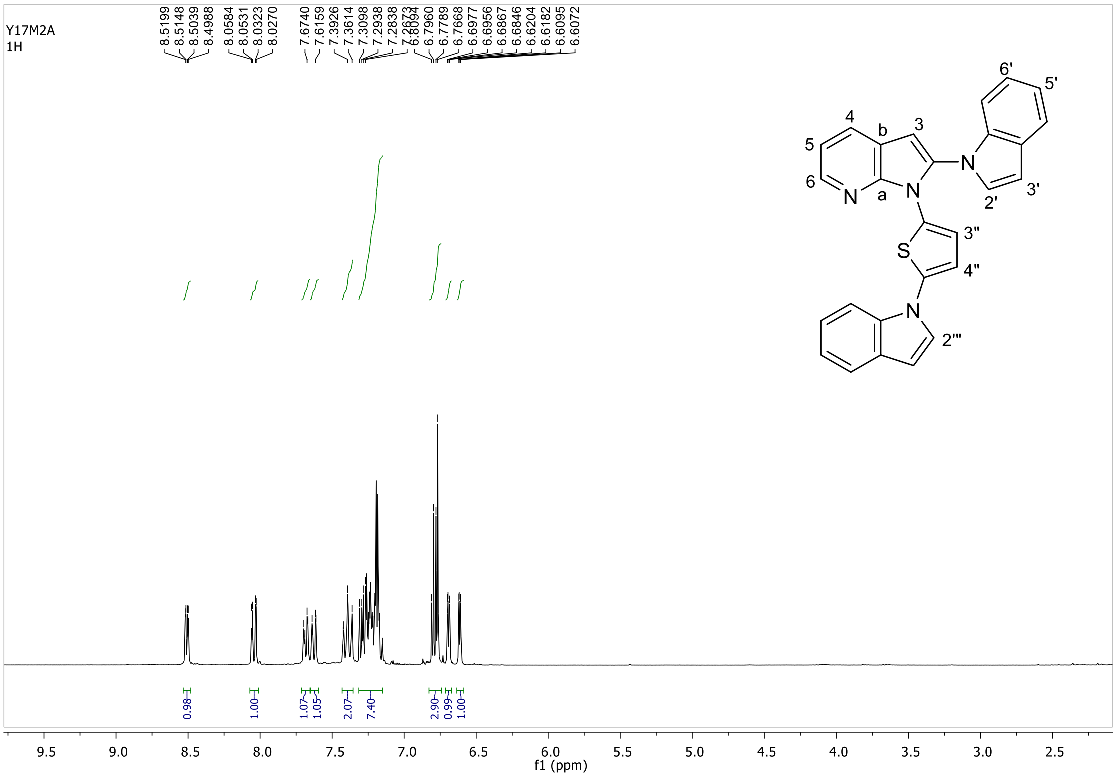

^{13}C NMR (75 MHz, $CDCl_3$)

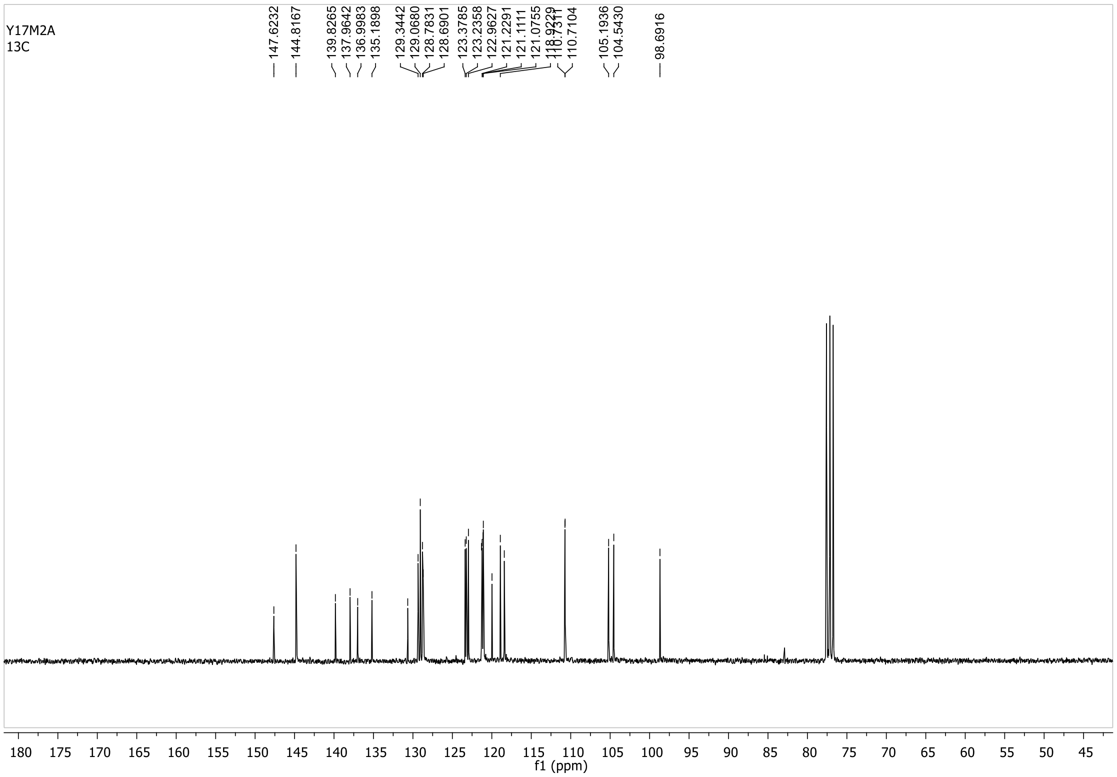

Calculated values of the Gibbs energies ΔG_{acid} [$kJ \cdot mo1^{-1}$] for deprotonation

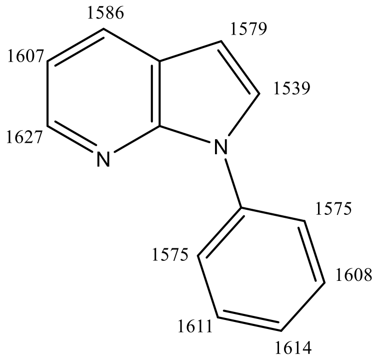

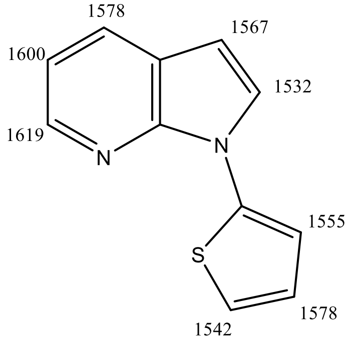

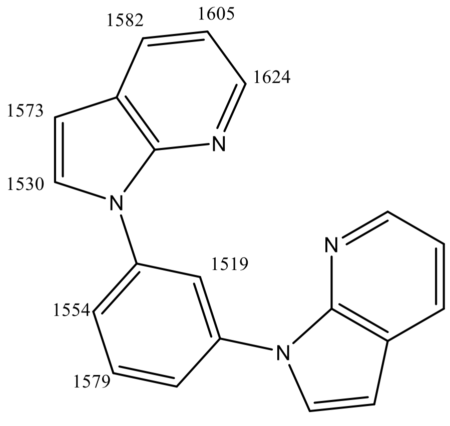

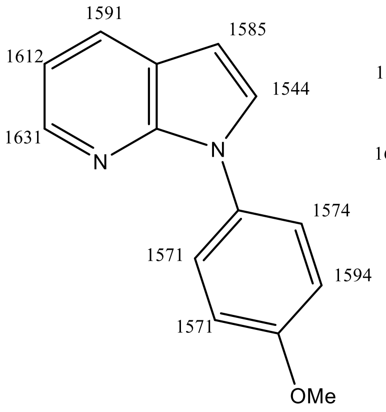

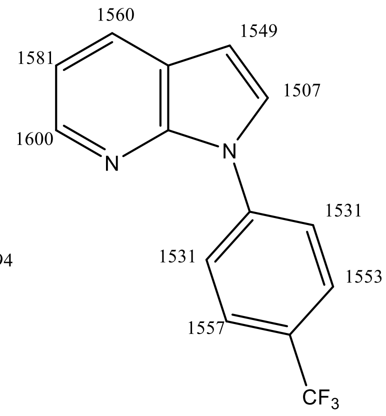

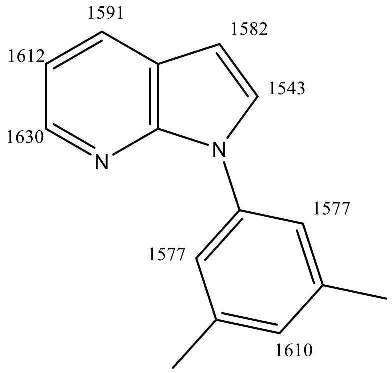

Supplement: Supplementary file 1 [file molecules-26-06314-s001.zip › molecules-1406059-supplementary.pdf]
